# Cross-Species Analyses Identify the BNIP-2 and Cdc42GAP Homology (BCH) Domain as a Distinct Functional Subclass of the CRAL_TRIO/Sec14 Superfamily

**DOI:** 10.1371/journal.pone.0033863

**Published:** 2012-03-27

**Authors:** Anjali Bansal Gupta, Liang En Wee, Yi Ting Zhou, Michael Hortsch, Boon Chuan Low

**Affiliations:** 1 Department of Biological Sciences, National University of Singapore, Singapore, Singapore; 2 Mechanobiology Institute, National University of Singapore, Singapore, Singapore; 3 Department of Cell and Developmental Biology, University of Michigan, Ann Arbor, Michigan, United States of America; University of South Florida College of Medicine, United States of America

## Abstract

The CRAL_TRIO protein domain, which is unique to the Sec14 protein superfamily, binds to a diverse set of small lipophilic ligands. Similar domains are found in a range of different proteins including neurofibromatosis type-1, a Ras GTPase-activating Protein (RasGAP) and Rho guanine nucleotide exchange factors (RhoGEFs). Proteins containing this structural protein domain exhibit a low sequence similarity and ligand specificity while maintaining an overall characteristic three-dimensional structure. We have previously demonstrated that the BNIP-2 and Cdc42GAP Homology (BCH) protein domain, which shares a low sequence homology with the CRAL_TRIO domain, can serve as a regulatory scaffold that binds to Rho, RhoGEFs and RhoGAPs to control various cell signalling processes. In this work, we investigate 175 BCH domain-containing proteins from a wide range of different organisms. A phylogenetic analysis with ∼100 CRAL_TRIO and similar domains from eight representative species indicates a clear distinction of BCH-containing proteins as a novel subclass within the CRAL_TRIO/Sec14 superfamily. BCH-containing proteins contain a hallmark sequence motif R(R/K)h(R/K)(R/K)NL(R/K)xhhhhHPs (‘h’ is large and hydrophobic residue and ‘s’ is small and weekly polar residue) and can be further subdivided into three unique subtypes associated with BNIP-2-N, macro- and RhoGAP-type protein domains. A previously unknown group of genes encoding ‘BCH-only’ domains is also identified in plants and arthropod species. Based on an analysis of their gene-structure and their protein domain context we hypothesize that BCH domain-containing genes evolved through gene duplication, intron insertions and domain swapping events. Furthermore, we explore the point of divergence between BCH and CRAL-TRIO proteins in relation to their ability to bind small GTPases, GAPs and GEFs and lipid ligands. Our study suggests a need for a more extensive analysis of previously uncharacterized BCH, ‘BCH-like’ and CRAL_TRIO-containing proteins and their significance in regulating signaling events involving small GTPases.

## Introduction

The functional complexity of living organisms is not only reflected by the number of genes or their protein products, but also by the cross-talk between them. This is signified by the fact that there are 1195 classes of known protein domain folds (based on latest release of Structural Classification of Proteins; SCOP database) belonging to 38221 Protein Data Bank entries of experimentally solved structures, indicating that multiple proteins tend to fold in a similar three dimensional structure. The ability of a protein module to interact with multiple proteinaceous binding partners potentially directs it to multiple cellular pathways and functions and thus makes it more versatile. A second level of complexity is added by the binding of non-protein molecules, which can modulate the three dimensional conformation of the protein domain and thus its cellular functions. The ‘Sec14 superfamily’ is one such large superfamily of protein modules [1], [2]. The members of this gene family have the ability to specifically bind multiple small hydrophobic molecules such as phosphatidylinositol (PI), tocopherol, retinaldehyde etc. [3]. The Sec14-protein (Sec14p) of yeast was the first identified member of this superfamily and is now known to be involved in exchanging PI and phosphatidylcholine (PC) between lipid membrane bilayers, making it essential for the transport of secretory proteins from the Golgi complex [4].

The lipophilic domain of Sec14p is also designated as a CRAL_TRIO domain (Pfam: PF00650, SMART: SM00516), which was first identified in cellular retinaldehyde binding protein (CRALBP) and Trio, a guanine nucleotide exchange factor (GEF). Other proteins such as tyrosine phosphatase (PTP) [5], α-tocopherol transfer protein (αTTP) [6], signaling regulator such as Ras GTPase activating protein (GAP) neurofibromatosis type-1 (NF1) and RhoGEFs (Trio, Dbl, Duo, Dbs, Kalirin) [7] also have similar three-dimensional structured protein domains and bind unique small hydrophobic ligands. High resolution x-ray crystal structures of CRAL_TRIO domains belonging to Sec14 superfamily have been determined from yeast and human representatives. These include Sec14p (PDB ID: 1AUA) [8] and Sfh1p (PDB ID: 3B74) [9] from yeast and α-TTP (PDB ID: 1R5L) [6], Sec14-PH domain of NF1 (PDB ID: 2D4Q) [10], CRALBP (PDB ID:3HY5) [11] and Sec14-L2/SPF (PDB ID:1OLM) [12] from human. Despite sharing only an average ∼30% sequence identity, these CRAL_TRIO domains exhibit highly similar three dimensional structures with an average root mean squared deviation of 2.6 Å. They include a shared α/β fold with alternating α-helices and β-strands, which usually defines a hydrophobic pocket for ligand binding. The CRAL_TRIO domain of human Sec14-L2 contains an additional C-terminal beta-sandwich domain [12]. At the N-terminus of many CRAL_TRIO lipid-binding domains, another conserved four helical domain has been identified, which is now referred as a CRAL_TRIO_N domain (Pfam accession: PF03765).

**Figure 1:**
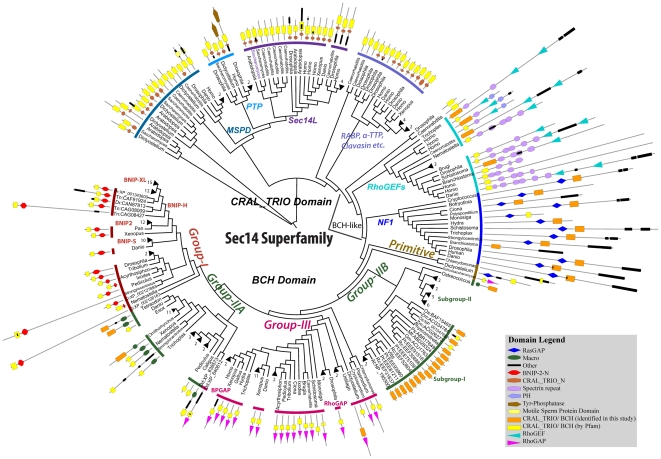
Phylogenetic tree of CRAL_TRIO and BCH domains from the Sec14 superfamily. This bootstrapped Neighbor Joining tree includes 175 BCH domains and 98 CRAL_TRIO/BCH-like domains from multiple organisms. The tree is displayed in a circular mode and different groups are marked by colored stripes. The clades with branch length <0.05 are collapsed and the number against each collapsed clade gives the number of collapsed branches. Branch lengths are ignored in order to maintain clarity. Against each branch the domain architectures of individual protein are shown as identified by the Pfam database (release 25) with a cut-off e-value ≤0.1. The Pfam database does not differentiate between CRAL_TRIO and BCH domains and thus both are indicated by yellow colored rectangles. However, if no such domain was identified by the Pfam database, we marked the annotations for BCH domains as determined by our analyses and they are indicated by an orange colored rectangle. The protein length is scaled. Eexcept when there is more than one protein from one genus (for these NCBI accessions are also given with name initials) only the generic names are given. The accession codes for remaining species/branches can be found in the Table S1. The abbreviations used are as follows; Dr: *Danio rerio*, Tn: *Tetraodon nigroviridis*, Ci: *Ciona intestinalis*, Dd: *Dictyostelium discoideum*, At: *Arabidopsis thaliana*, Rc: *Ricinus communis*, Pt: *Populus trichocarpa*, Gm: *Glycine max*, Mt: *Medicago truncatula*, Ps: *Picea sitchensis*, Zm: *Zea mays*, Os: *Oryza sativa*. This phylogenetic tree shows the distinct clustering of BCH domains from CRAL_TRIO domains. The three BCH subgroups are group I, group II and group III respectively and distinct groups within the CRAL_TRIO domain are also marked accordingly. Each cluster represents a distinct domain architecture. Pfam does not recognize the complete domain in BCH groups. The CRAL_TRIO_N domain, which is characteristically associated with CRAL_TRIO domains, is also missing in BCH and BCH-like (NF1 and RhoGEF) proteins. Similar to NF1 protein, *Dictyostelium discoideum* has an ancestral BCH sequence, which is associated with a RasGAP domain.

The BNIP-2 and Cdc42GAP Homology (BCH) domain was initially recognized as a region of high protein sequence homology between BNIP-2 (BCL2/adenovirus E1B 19kDa interacting protein-2) and Cdc42GAP/p50RhoGAP [13]. This structural protein domain is usually classified as ‘Sec14-like’ domain. However, it exhibits only 14% sequence identity with the CRAL_TRIO domain of the *Saccharomyces cerevisiae* Sec14p protein. It is approximately 150 amino-acid in size and is known to be involved in the control of diverse aspects of cell dynamics such as apoptosis [14], [15], cell migration [16], morphogenesis [17], [18], [19], endocytosis [20], intracellular trafficking [21], [22], cell transformation [23] and differentiation [24]. This diverse range of functions appears to be mediated by its unique ability to interact with small GTPases and their regulators, both GAPs and GEFs [15], [18], [19], [23]. Through these direct protein-protein interactions, BCH domains control the activation/inactivation of Rho GTPases particularly those that are involved in the organization of the actin cytoskeleton [25], [26]. For example, the BCH domain of human BNIP-2 promotes Cdc42 activation required for cell protrusions [18] and muscle cells differentiation [24]. In BNIP-Sα, it also maintains RhoA activity by displacing Cdc42GAP/p50RhoGAP leading to cell rounding and apoptosis [15], [19]. In contrast, the BCH domain in BNIP-XL binds Lbc RhoGEF and prevents RhoA activation [23]. Importantly, a mutation in the BCH domain of the caytaxin protein (also called BNIP-H) is associated with an intriguing neurological disorder, Cayman ataxia [27]. Adding to their biological significance, both BNIP-2 and BNIP-XL are cleaved by caspases, releasing their BCH domains that could lead to apoptosis [28] whereas BNIP-2 is also cleaved by granzyme B during the natural killer cell-mediated killing to tumor cells [29]. However, unlike CRAL_TRIO domains of the Sec14 superfamily, BCH domains are not known to interact with lipid molecules and their postulated non-protein ligands are currently unknown. Thus, based on distinct functional properties and their low sequence similarity, there is an ambiguity in classifying BCH together with conventional CRAL_TRIO domains. The BCH domains of many proteins have been included in CRAL_TRIO entry of domain databases such as Pfam (release: 25) (PF00650) and SMART (SM00516). However, the CRAL_TRIO entry in Pfam fails to recognize protein domains in a number of proteins, which we clearly identify as the BCH domains (e.g. XP_001512063, orange boxes in figure 1).

This article attempts to highlight the unique sequence and structural features of BCH domains and outlines, which distinguish them from the CRAL_TRIO domains of the Sec14 superfamily. We have identified a large number of BCH domains from multiple organisms and a large dataset has been used to describe the potential evolutionary relationship between the BCH and CRAL_TRIO domains. BCH domains can be assigned to three distinct subgroups and we further investigate the divergence of these subgroups from their ancestral precursor genes, leading to a wider functional specialization. A possible pathway of BCH domain evolution is being proposed and we identified the most likely point of divergence from CRAL_TRIO-like proteins. In addition, we present 3-dimensional structural models for all three subgroups of BCH domains. Based on the discussed features, it will now be possible to distinctly identify BCH and CRAL_TRIO domains within different proteins.

## Materials and Methods

### Identification of BCH and CRAL_TRIO domain containing proteins from the GenBank database

The protein Blast search was carried out in the GenBank database of NCBI to identify proteins containing putative BCH domains. The BCH domain of Human BNIP-2 protein (NCBI accession: NP_004321, amino-acid 167 to 314) as defined in our earlier published work [13] was used as a query. With the e-value cut-off of 1, our search picked up more than 400 proteins. These were screened to define a dataset for more detailed analysis. The sequences were grouped based on a 95% level of redundancy using CD-hit [30] for easier comparison. Each group was manually analyzed and the sequences were screened by iterative multiple sequence alignments. Very small sequences (<100 amino-acid), which are unlikely to fold into defined BCH domains, and sequences with long gaps in multiple sequence alignment comparisons were discarded. This stringent selection criteria including pair-wise sequence alignment with representative BCH domains of human BNIP-2 (NP_004321), p50RhoGAP (BAG60756) and GDAP (NP_060156) proteins ensured an effective elimination of false positives in our dataset. This resulted in a defined dataset of 175 proteins with putative BCH domains. Since our search was able to identify even the most distantly related BCH domains, we did not use PSI-BLAST. Moreover, it is also likely to introduce noise in the multiple sequence alignment, which we intend to use for characterizing BCH domains. The information about the number and the position of introns within these BCH domains was directly extracted from their corresponding entries in the NCBI database. Previously, the introns have been suggested to mark the boundary of functional domains [31]. Thus, we considered intron insertions only within the sequence defined by two introns as N and C terminal ends of the putative BCH domain.

Similar searches were carried out to identify CRAL_TRIO domain containing proteins. However, we restricted this search to the NCBI's RefSeq database [32] and to eight model organisms, *Dictyostelium discoideum*, *Drosophila melanogaster*, *Danio rerio*, *Xenopus laevis*, *Arabidopsis thaliana*, *Caenorhabditis elegans*, *Saccharomyces cerevisae* and *Homo sapiens*. The PSI-BLAST search using yeast sec14p (NCBI accession: NP_013796) query identified multiple members belonging to Sec14 superfamily. A large number of protein homologs were identified in *Arabidopsis thaliana* (>30) and *Drosophila melanogaster* (>20). In order to avoid a data bias, only few representative sequences were selected from these organisms. This was based on clustering in phylogenetic trees, which were produced separately for each species. Outliers (defined as sequences with no CRAL_TRIO domain identified by the Pfam database) and shorter hits of length <150 amino-acids were excluded from further analysis. Multiple isoforms belonging to the same protein were also discarded. Separate searches were carried out for identifying members of the NF1 and RhoGEF subfamilies using human NF1 (accession: AAB59558) and Trio (accession: NP_009049) as query. An additional set of 98 proteins from the Sec14 superfamily was generated for constructing phylogenetic trees and for further comparative studies.

### Multiple sequence alignment and phylogenetic analysis

The CRAL_TRIO and BCH domain sequences were aligned using the Clustal v2.0 multiple sequence alignment algorithm [33]. The pair-wise alignments were computed in slow and accurate mode. The N and C terminal ends of BCH domain are ambiguous and difficult to identify in individual proteins. The N-terminus of the yeast CRAL_TRIO domain forms a long loop, which connects it with the CRAL_TRIO_N domain at its N-terminus. Thus, the long poorly unaligned terminal ends were removed and an alignment of block of length 297 positions was retained for constructing phylogenetic trees. The multiple sequence alignment is referred to in the Table S2. The phylogenetic trees were created by the Neighbor Joining (NJ) method [34] as implemented in Clustal v2.0 while ignoring gapped columns. The NJ tree was bootstrapped by 1000 bootstrap trials to confirm the robustness of branches and was displayed by iTOL v1.8 (http://itol.embl.de/) [35]. All alternative splice forms were excluded from the analysis. The circular tree was displayed with the branches collapsed if the average distance to leaves was <0.05. This was done for keeping the presentation clear. The branch lengths were also ignored in the final display. The sequence logo was created by WebLogo (http://weblogo.berkeley.edu/). All sequence identities are calculated by MegAlign tool of Lasergene suit from DNASTAR Incorporation (http://www.dnastar.com/).

### Three-dimensional structure prediction

No clear template with significant sequence homology was identified for modeling BCH domains. Thus, structures were predicted using the I-TASSER (Iterative threading assembly refinement) [36] and the ROBETTA [37] servers. These programs are available as the web based tools for *De Novo* automated protein structure predictions. Both methods have performed well in CASP experiments [38], [39], [40], [41] and have resulted in structural models by combining methods of threading, *ab initio* modeling and further refinement. The multiple threading alignments in I-TASSER were created by LOMETS algorithm to identify the structure fragments from a library, which was assembled by replica exchange Monte Carlo simulation methods. The predicted models were simulated by TASSER iterations for optimization to remove steric clashes between atoms and refining side-chain rotamer conformations. The ROBETTA server is part of the Rosetta folding program. It uses a Ginzu protocol [42] to establish homology between experimentally known structures and regions on amino acid sequences, which might fold into putative domains. A fragment library was used for searching the conformational spaces for loop regions and also for those regions for which no structural homolog could be identified.

The structures of the BCH domains (length 173 amino-acid) from human BNIP-2 (NCBI accession: NP_004321), RhoGAP (NCBI accession: BAG60756), GDAP (NCBI accession: NP_060156) proteins were predicted using I-TASSER with no specified templates and restrains. Only the core BCH domain of BNIP-2 was predicted using the ROBETTA server. The I-TASSER found the highest sequence identity (17%) among all available structures with the domain of human neurofibromatosis type 1 protein (PDB ID: 2D4Q) [10]. The quality of the model was assessed with PROCHECK [43]. Out of five predicted models, the one with high C-score (confidence score) and low TM-score value as calculated by I-TASSER was selected for further analysis and comparisons. The C-score was calculated from the threading template alignments and convergence parameters of assembly simulations. A higher score signifies better alignment with the template and a faster convergence of structures. The TM score [44] is similar to root mean squared deviation measurement except that a weighting scheme is used for reducing the effect of local errors, which are caused by different orientations of mobile parts such as loops and termini. The resulting structures were further compared using the Dali structure alignment method [45].

## Results

### Identification and Phylogenetic clustering of BCH vs CRAL_TRIO domains

Our search of the entire non-redundant GenBank database identified 175 proteins containing a putative BCH domain. BCH domains were identified in a wide range of eukaryotic species, including slime molds, fungi, animals and plants. In contrast, no BCH domain-containing proteins were identified from bacterial species. To confirm the absence of BCH domains in prokaryotes, we carried out Blast searches of all completed Archaea and bacterial whole genome sequences using four BCH domain sequences of *Dictyostelium discoideum* (a slime mold) as query sequences and an e-value cut-off of >2. This analysis yielded no significant hit and therefore suggests the absence of BCH domains in the bacteria and the Archaea. This large dataset was taken as a resource for characterizing and classifying the BCH domain within the Sec14 superfamily. In addition, we also identified 98 proteins from eight representative organisms that belong to other sub-groups within the Sec14 superfamily. These were aligned with the dataset of 175 BCH domains, which based on their alignment with CRAL_TRIO domains were isolated from their full-length proteins. This set of BCH domains has longer amino-acid sequences as these sequences include N-terminal amino-acid extensions than previously reported [13]. We show in this article that this extra N-terminal extension is essential for forming the complete three-dimensional BCH domain structure. Hence, our analysis was carried with the extended BCH domain. The multiple sequence alignment of CRAL_TRIO/BCH domains can be found in the Table S2.

A phylogenetic tree including 175 BCH domains and 98 CRAL_TRIO domains was constructed as described in the methods section (Figure 1). The tree indicates several distinct clusters of BCH domain-containing proteins (lower half of tree), which are distinct from CRAL_TRIO domain proteins (upper half of tree). The previously defined groups of CRAL_TRIO domains are marked as CRALBP (cellular retinaldehyde binding protein), MSP (motile sperm protein domain containing protein), PTP (Phosphotyrosine phosphatases) and Sec14p-like, (see Figure 1). We calculated the sequence identity between all pairs of domain sequences and found that CRAL_TRIO sequences only share an average of 12% identity with BCH domain sequences (based on ∼3000 comparisons, Table 1). This analysis indicates that BCH domains are related, but clearly distinct from CRAL_TRIO domains. A comparison among BCH domain sequences indicates a much higher amino-acid sequence identity (average: 38%) across divergent organisms, Table 1. Each sub-group of CRAL_TRIO group is functionally distinct and interacts with unique ligands; e.g. the CRAL_TRIO domain of Sec14L group binds to phosphotidylinositol, αTTP has affinity for tocopherol, CRALBP binds with retinaldehyde [3]. The ligand specificities of the RhoGEFs and RasGAP (represented by NF1 proteins) groups is currently unknown. The position of these groups within the phylogenetic tree is particularly interesting as they cluster between the BCH and CRAL_TRIO domain groups (Figure 1). Also, the domains of the RhoGEF and RasGAP groups share low sequence identities with both BCH and CRAL_TRIO domains (Table 1). Importantly, no RasGAP or RhoGEF proteins were identified in Blast searches using either BCH or CRAL_TRIO domains as queries. Due to a higher sequence homology with BCH domains and missing CRAL_TRIO_N domain (characteristic of CRAL_TRIO groups), we classified these groups as ‘BCH-like’. As indicated by their presence in slime molds, these genes arose early in evolution. Further, more than ten CRAL_TRIO domain-containing proteins were identified in *Drosophila melanogaster* and *Caenorhabditis elegans*. In contrast, only a single BCH domain protein is encoded in the genome of the nematode *Caenorhabditis elegans*.

**Table 1:** Average sequence identity within and across the groups of Sec14 superfamily.

	Group-I	Group-IIA	Group-IIB	Group-III	NF1	RhoGEFs	CRAL_TRIO
**Group-I**	64.8 *(2278)*						
**Group-IIA**	24.8 *(2040)*	57.4 *(435)*					
**Group-IIB**	24 *(2244)*	28.1 *(990)*	43.3 *(528)*				
**Group-III**	33.4 *(2788)*	30.6 *(1230)*	24.2 *(1353)*	49.8 *(820)*			
**NF1**	15.1 *(884)*	18.6 *(390)*	16.9 *(429)*	16.9 *(533)*	36.4 *(78)*		
**RhoGEFs**	12.2 *(1088)*	14.2 *(480)*	12.1 *(528)*	14.5 *(656)*	12.9 *(208)*	41.5 *(120)*	
**CRAL_TRIO**	13.3 *(4692)*	12.2 *(2070)*	12.7 *(2277)*	11.2 *(2829)*	13.8 *(897)*	14.9 *(1104)*	21.4 *(2346)*

The average sequence identity is given as calculated between all pairs of sequences. The numbers in bracket refers to the number of comparisons made in each group. The values are higher when compared within the group. The domains of NF1 and RhoGEF groups share comparable sequence similarity with BCH and with CRAL_TRIO domains. Thus, the domains of these proteins were referred as ‘*BCH-like*’.

### BCH domains evolved to form three distinct functional groups

Similar to CRAL_TRIO, the BCH domain is completely absent in prokaryotes. The most primitive BCH domain was identified from slime mold (*Dictyostelium*), coanoflagellate (*Monosiga*), alveolates (*Plasmodium*, *Cryptosporidium*), green alga (*Chlamydomonas*) and yeast. Similarly, CRAL_TRIO domains were identified in many lower species of alveolates including *Babesia* (XP_001612272), *Tetrahymena* (XP_001018732), *Paramecium* (XP_001427613, XP_001454548) and the diatom *Phaeodactylum* (XP_002182927). This indicates that BCH domains evolved from their ancestors more than 1500 Mya ago with the appearance of Protists [46]. In the phylogenetic tree shown in Fig. 1, BCH domains are further sub-divided into three distinct subgroups, which based on their phylogenetic clustering and associated protein domains were classified as group-I, group-II and group-III BCH domains. Group-II BCH domain proteins form clusters distinct from plants and animals and therefore were designated as group-IIA (belonging to animal species) and group-IIB (belonging to plant species). Both groups have ‘BCH-only’ domains. In animals this is peculiar to insects, whereas in plants these sub-groups exist in all lineages, from lower algae to higher monocots.

**Group-I BCH:** Since it is found at the C-terminus of the BNIP-2 family of proteins, the BCH group-I is also referred as BNIP-2-BCH. The four BNIP-2 family proteins include BNIP-2, BNIP-S (BNIP-2-Similar), BNIP-XL (Extra Long proteins) and BNIP-H (these BNIP-2-Homologous proteins are also designated as Caytaxins). All these proteins are involved in Rho GTPase regulation. Their distinct clustering into four subgroups in vertebrates has been observed for many gene families [47] and appears to be the result of two genome-wide duplications before the diversification of the vertebrate phylogenetic tree. It suggests that these unique BNIP-2-type subgroups might have acquired different functional specializations. The group-I BCH domains show high average sequence identities between distantly related organisms (average: 65%). As evident from the zero branch lengths in the phylogenetic trees of all three groups (figure S2), BCH domains are under a strong selection pressure in mammals to be conserved. Mammals also encode a large number of BCH domain-encoding genes in their genomes (see Figure S1). Invertebrate genomes (Insects, *Nematostella*) appear to contain only one BNIP-2-type gene which does not cluster with any of the four sub-groups (see figure S2). Plants are devoid of group-I BCH domain genes (also group-III). The most primitive organism with a group-I BCH gene is a Cnidarian (*Nematostella*) and all four isoforms appeared first in teleosts (*Danio*). *Ciona*, a tunicate has two group-I BCH isoforms suggesting the divergence could have occurred from *Ciona-*like ancestors. Interestingly, no group-I genes were identified in more primitive invertebrate species, such as nematodes. However, such species have other BCH domain encoding genes, such as group-III BCH proteins. Group-II and III appears to be older than group-I as they were identified in more primitive forms. The sequence similarities within the members of group-I BCH domains is higher (64.8%) compared to group II (50.3%) and III (49.8%). Group-I BCH domain-containing proteins show unique associations with other protein domains also, among other examples, Spo7 (Pfam accession: PF03907) domains at the C-termini of BNIP-S proteins in a few mammalian species and DHH and DHHA2 domains at the N-termini in a few BNIP-H and BNIP-XL proteins.

**Group-II BCH:** Group-II BCH domains were identified in plants, animals and also in multiple lower organisms. The domains from plants and animals form two distinct clusters in the phylogenetic tree and were designated as group-IIA (animal's group-II BCH) and group-IIB (plant's group-II BCH) (see Figure 1 and Figure S2). Similar to group-I BCH domains, group-II protein domains are also usually located at the C-terminal end of proteins and are associated with macro domains at their N-termini, which in animals are called ganglioside-induced differentiation-associated proteins (GDAP). In plants, group-II BCH domains are found in the family of Appr1p processing enzymes (AEP). This is the first report of BCH domains found in plants. Both plants and animals have one distinct clade in the phylogenetic tree depicted in Figure 1, in which the BCH domain is not associated with any other protein domain. In animals, this ‘BCH-only’ group is only found in insect species. These insect BCH-only domain proteins appear to have arisen by the loss of other associated protein domains. In plants, most BCH-only proteins were identified in higher plants. However, as they also can be found in green algae, they must have arisen much earlier in evolution. These genes might have arisen either by the loss of the macro domain (as appears to be the case in insects) or from their slime-mold-like ancestors, which associated with a macro domain later in evolution. The BCH-only proteins are probably essential in plants. However, they disappeared in most animal species. Therefore, it will be interesting to analyze their role in insects and in plants. Plants lack other types of BCH domain proteins, specifically group-I and group-III representatives. Possibly due to the whole genome duplication, *Populus* encodes 6 BCH domain genes [48]. Group-IIB BCH domain proteins are found in lower organisms, like slime molds (*Dictyostelium*), moss (*Physcomitrella*) and green algae (*Ostreococcus*). Similar to group-I proteins, the mammalian group-II BCH domain proteins exhibit a high sequence similarity amongst themselves (>95%), indicating a more recent evolutionary split.

**Group-III BCH:** Group-III BCH domains are unique as they are located at the N-terminus of proteins. As they are associated with a RhoGAP domain at their C-terminus, they are referred as RhoGAP-type BCH domains. They are more divergent in mammalian species and other higher species and express two group-III BCH isoforms, which are associated with RhoGAP and BPGAP (BCH domain containing, Proline-rich and Cdc42GAP-like protein) [17] domain respectively. Nematodes (*Brugia*, *Caenorhabditis*) have only one group-III BCH domain genes. *Trichoplax*, a Placozoan, has group-II and group-III BCH proteins, but no group-I BCH domain protein. No BCH domain is associated with a RhoGAP domain in plants. It is interesting to point out that in plants, RhoGAPs of REN family contain a Pleckstrin homology (PH) domain [49]. NF1 proteins, the closest relatives of BCH domain proteins are also associated with a PH domain, which plays a crucial role in gating the lipid-binding cavity. The other plant RhoGAPs are commonly referred to as RopGAPs (Rho of plants) and are associated with a Cdc42/Rac interactive binding (CRIB) motif at their N-terminus [50]. This motif has not been observed in animal RhoGAPs. By targeting RopGAPs to small GTPases through direct interactions [51] and through interactions with other downstream effectors [52], this CRIB motif is postulated to contribute to the regulation of the GAP activity. Recently it has been shown to play a role in forming high affinity complexes with specific Rho proteins and GAP domains and acts as a lid for binding and releasing Rho of plants [53]. This is similar to BCH domains, which are proposed to modulate the GAP activity in p50RhoGAP through their direct interaction with Rho [19]. Previously, a part of BNIP-2 BCH domain has been found to share sequence similarity with the CRIB motif [18]. Therefore, it is tempting to speculate that in plants the function of group-III BCH proteins was taken over by CRIB proteins.

Based on overall protein domain architecture and phylogenetic clustering, we hypothesize that the co-evolution of associated domains with BCH domains resulted in an additional functional divergence and complexity of the gene family. As CRAL_TRIO proteins diverged to bind multiple different hydrophobic molecules, we speculate that BCH proteins also have evolved to bind multiple ligands, many of which still need to be identified. The BCH family is the most distantly related subgroup within the Sec14 superfamily and is most closely related to NF1. Interestingly, similar to NF1 the slime mold BCH domain is also associated with a RasGAP domain (XP_645456). None of the group-I BCH is found in *Dictyostelium discoideum*. We therefore speculate that BCH might have diverged from an NF1-like ancestor, which had a RasGAP domain. Later, they probably associated with RhoGAP and macro domains through chromosomal recombination.

### A Sequence logo that distinguishes CRAL_TRIO and BCH domains

**Figure 2:**
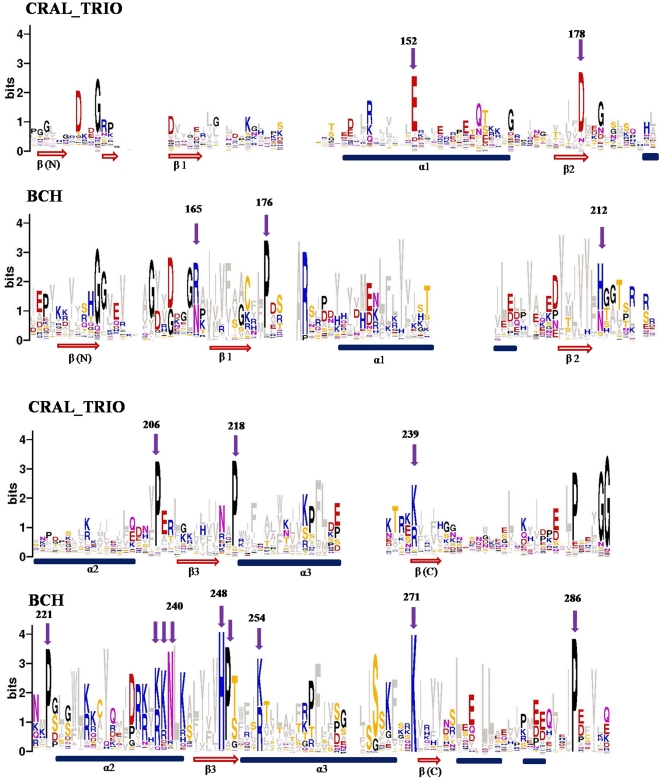
Sequence logos of CRAL_TRIO and BCH domains. The sequence logos derived from 175 BCH and 78 CRAL_TRIO domain sequences are shown in this figure. The conserved residues are marked with arrows and the numbering is given according to the yeast Sec14p protein (NCBI accession: NP_013796) for CRAL_TRIO domains and the human BNIP-2 protein (NCBI accession: NP_004321) for BCH domains. The approximate positions of α-helices and β-beta strands are indicated at the bottom by blue cylinders and red arrows. In order to avoid any biased data, the ‘BCH-like’ groups (NF1 and RhoGEFs) were excluded from the logo calculation. These logos reveal characteristic differences between BCH and CRAL_TRIO domains. Unique positions within the two groups are marked by arrows. BCH domains have a unique signature motif R(R/K)h(R/K)(R/K)NL(R/K)xhhhhHPs in which ‘h’ refers to any large and hydrophobic residue and ‘s’ is a small and weekly polar residue (A, T, G, S). This motif is missing in CRAL_TRIO domains. The motif contains a patch of positively charged residues referred to as an Arg/Lys patch. Similarly, as exemplified by the aromatic residue in the middle of three α-helices, many of the hydrophobic residues (shown in grey) are conserved at various positions. The conservation of long and hydrophobic residues in the β-strands provides a hydrophobic surface.

Multiple BCH domain sequences are included in the Pfam (release: 25) entry of CRAL_TRIO domains (Pfam accession: PF00650) and used for constructing a common domain profile. However, we postulate that CRAL_TRIO domains have distinct features from BCH domains. This hypothesis is supported by our finding that the HMM profile for CRAL_TRIO in the Pfam (release: 25) database fails to identify CRAL_TRIO domains in 4 RhoGEFs, 12 NF1 and 20 out of 175 BCH proteins (indicated by orange rectangles in Figure 1). However, all these proteins belong to the RhoGEF, NF1 and BCH groups and in many cases only a subset of sequence was recognized as a CRAL_TRIO domain (small yellow rectangles in Figure 1). Here, we have created separate sequence logos for BCH and CRAL_TRIO domains and show that the two domains are clearly distinguishable from each other. The sequence logos were created from 69 CRAL_TRIO (excluding NF1 and RhoGEFs) and 175 BCH domain sequences. NF1 and RhoGEF sequences were excluded as they exhibit homology with both BCH and CRAL_TRIO domains; Table 1. The conservation of unique residues in BCH domains is marked with arrows in Figure 2 and position values are given in Table 2.

**Table 2:** Conservation of residues characteristic of (a) CRAL_TRIO and (b) BCH domains of the Sec14 superfamily.

(a) Residues conserved within CRAL_TRIO domains			(b) Residues conserved within BCH domains		
	CRAL_TRIO	BCH		BCH	CRAL_TRIO
E152	**84.1**	-	(R/N)165	**90.1**	-
D178	**94.2**	*-*	P176	**87.3**	-
P206	**91.3**	-	(H/N)212	**89**	-
P218*	**89.9**	*90.6*	P221	**87.8**	**-**
(K/R)239	**85.5**	**99.5**	(K/R)238	**92.8**	-
P(261)	**81.2**	**97.8**	(K/R)239	**87.3**	-
G(265)	**89.8%**	**-**	(N)240	**87.9**	-
G(266)	**97%**	**-**	(H)248	**98.3**	-
			(K/R)271	**99.5**	-

The numbering of residues is given according to the positions of residues in yeast Sec14p and Human BNIP-2 proteins in Table columns (a) and (b) respectively. Only values above 50% are given in the table. *Proline residues of CRAL_TRIO and BCH domains are not in single column in multiple sequence alignment, they are conserved at one position apart (Figure 2).

The BCH sequence logo reveals a clear pattern of the characteristic residues that are conserved within the BCH domain and are missing in CRAL_TRIO domains. Examples are: P176, P221 and H248 (see Table 2). Additional characteristic positions are marked with arrows in Figure 2. On the other hand, CRAL_TRIO domains also have signature residues, which are absent or poorly conserved in BCH domains. For example, E152 (84%) and D178 (94%) are characteristic for CRAL_TRIO domains and are missing in BCH domains. However, the C-termini of both domains show similarities in their amino-acid sequences. We determined R(R/K)h(R/K)(R/K)NL(R/K)xhhhhHPs as a unique BCH domain sequence motif. ‘h’ refers to any large and hydrophobic residue and ‘s’ is small and weekly polar residue (A, T, G, S). The motif forms a patch of positively charged residues, named the ‘Arginine/Lysine patch’. It is conserved in BCH domains from slime mold to mammalian species. These observations suggest that BCH represents a distinct domain from CRAL_TRIO and that it diverged from CRAL_TRIO-like ancestors and acquired unique functional capabilities. The uniquely conserved residues within BCH domains can be used as hallmark signatures to identify BCH domains in unknown protein sequences. As an example; the domain of *Dictyostelium discoideum* RasGAP protein (XP_645456: 509–711) was previously classified as a CRAL_TRIO domain [10]. However, based on presence of characteristic sequence motifs, the present study clearly identifies the corresponding region as a BCH domain.

The conserved residues within CRAL_TRIO domains have structural and functional implications and the absence of these residues in BCH domains suggests their distinct nature. For example, position E152 (corresponding to E141K mutation in αTTP) is associated with the disease ‘Ataxia with Vitamin E deficiency’ (AVED) [54]. The large amino-acid side-chain at position G266 (>95% conserved in CRAL_TRIO) is known to cause steric hindrance and destabilization of the hydrophobic pocket [8]. This is also confirmed by an in-silico molecular dynamics simulation study of mutant G266D Sec14p [55]. This important Glycine residue is completely missing in BCH domains (also in the BCH-like groups). This indicates that functional differences governed the evolutionary divergence of BCH and CRAL_TRIO domains.

### The BCH domain diverged from the CRAL_TRIO domain as a distinct functional unit

Many new genes diverge from their preexisting ancestors by gene duplication events and often acquire unique functional capabilities. These changes are usually reflected in their amino-acid sequences. As discussed, BCH domains exhibit functional features, which are not found in other members of Sec14 superfamily, such as GTPases binding activity, homo and heterophillic interactions [56] etc. In addition, in the phylogenetic tree of Sec14 superfamily, BCH protein domains form a cluster distinct from CRAL_TRIO protein domains. No hit was obtained from the CRAL_TRIO group of Sec14 superfamily using BCH sequences as a query in PSI-BLAST searches and vice versa, indicating that they are distantly related groups. BCH domains are associated with BNIP-2-N (the N-terminal conserved domain within the BNIP-2 proteins), macro or RhoGAP domains at their N- or C-terminus. In contrast, CRAL_TRIO domains are characteristically associated with a four helical bundle domain at their N-termini, which is called CRAL_TRIO_N. This CRAL_TRIO_N domain is thought to be involved in the stabilization of the lipid binding cavity, which is situated within the CRAL_TRIO domain [8]. Together these sub-domains define the complete ‘Sec14-domain’ [57]. This functionally important CRAL_TRIO_N domain is absent in BCH domain-containing proteins and also in the RhoGEF and NF1 groups (BCH-like) of Sec14 superfamily. These observations, combined with the distinctions as identified by the sequence logo, establish the fact that the BCH domain descended as a distinct functional domain from CRAL_TRIO domain group.

### Evolutionary clues from the gene structure of BCH domain-encoding genes

**Figure 3:**
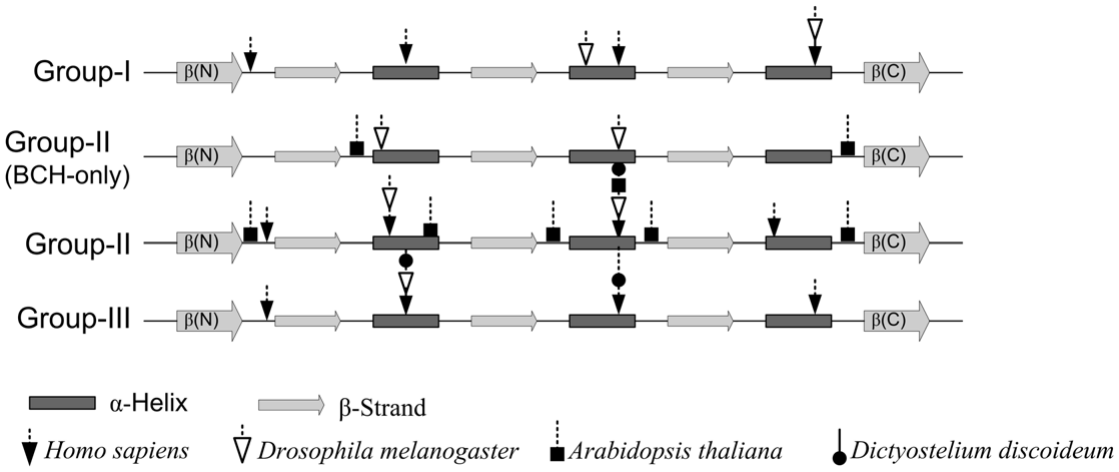
Gene structure of BCH domains. The gene structures of BCH domains are shown for four representative organisms, *Homo sapiens*, *Drosophila melanogaster*, *Arabidopsis thaliana*, *Dictyostelium discoideum*. Their accessions are NP_056040 (*Hs* group-I), CAQ06715 (*Hs* group-II), NP_060156 (*Hs* group-III), ABY20545 (*Dm* group-I), NP_724599 and NP_724597 (*Dm* group-II), NP_648552 (*Dm* group-III), NP_564960 and NP_195300 (*At* group-II), XP_638573 (*Dd* group-II), XP_645940 (*Dd* group-III). The positions of introns are marked by arrows on the secondary structure (not scaled) of the BCH domain. With few exceptions, BCH domains of other organisms within the same group exhibit similar intron insertion patterns. Plants and lower organisms have no group-I BCH representatives. Except the ‘BCH-only’ gene, which has two introns, the BCH domain genes of *Dictyostelium discoideum* (XP_645456: primitive-type, with RasGAP domain and XP_640612) are intronless. Similar insertion positions in plant group-II genes suggest that they might have evolved from ‘BCH-only’ genes through intron insertions and association with macro domain later. These introns were preferentially inserted in the three alpha-helices and in loops (in plants).

The number and position of introns revealed additional clues about the divergence of BCH domain-encoding genes (Figure 3). The absence of introns in more primitive species (*Dictyostelium*) indicates that the ancestral BCH sequence did not contain introns and different BCH domains evolved from their ancestors through intron insertion. This is similar to that observed by Qiu et al. in their study of 677 eukaryotic protein coding genes from 10 families [58]. Only fungal species have >5 introns. In general, the introns were inserted in the middle of protein alpha helices in animal species and in polypeptide loops in plant species. However, the insertion sites are not strictly conserved across all BCH sequences/groups and there are exceptions to this observation. No intron insertions were observed in β-strands. This might indicate that these strands form the conserved core of the BCH domain and are under stronger evolutionary pressure compared to helices and mobile loops. It is interesting to note that loop (connecting a strand with helix) in each of the exon in BCH domain has one highly conserved Proline residues (P176, P221, P249) followed by an Arginine/Lysine. This suggests the possibility that preexisting exons evolved by duplication. An interesting example in our dataset is group-IIA BCH domain of the mosquito *Culex quinquefasciatus* (accession: XP_001847511). It has two exons (amino acid 89 to 186), which are identical to two other exons (amino acid 187 to 284). A tandem duplication event has been reported in the NF1 gene, which results in Watson and Noonan's syndrome [59]. However, this is located in the linker region that joins the ‘BCH-like’ domain with the PH domain [60] and corresponds to the C-terminal α-helix of the BCH domain. A comparison of intron insertions in plants with animal BCH domain also indicates that they have diverged along separate line of evolution. In plants, the introns were preferably inserted in protein loops. Plant subgroup-II genes have four or more introns, while BCH-only genes in plants (subgroup-I) have only two introns. BCH-only genes in insects also have two introns and the insertion sites are different from the plant BCH-only group genes, suggesting that they diverged from separate ancestors. Similarities in the intron insertion patterns of group-I and group-III BCH genes support the hypothesis that they have evolved by domain swapping.

We also observed insertions at the C-termini of BCH domains through alternative splicing (figure S3). The conservation of insertion sequences in BCH protein isoforms might indicate a conservation of their functional plasticity across a wide range of species. Since it does not appear to contribute to any lipid binding activity, it might alternatively be involved in mediating important protein-protein interactions.

### Three-dimensional structure of BCH domain

So far, no molecular structure for any BCH domain has been experimentally determined and published. The three dimensional structures for NF1, CRALBP and Sec14p are highly similar for their core region, which excludes the N and C terminal regions. In the absence of clearly defined sequence homologies, the molecular structures for all three subgroups of BCH domains i.e. group-I (HsBNIP-2: NP_004321), group-II (HsGDAP: NP_060156), group-III (HsRhoGAP: BAG60756) were calculated and predicted using I-TASSER server [36] which uses a de-novo method for its predictions combining laws of comparative and ab-initio modeling. In this context, it will be interesting to have a method, which can predict the three dimensional structural models based on the evolutionary information within the sequence alone. One such method has been recently proposed by Marks et al. [61]. In our I-TASSER protocol, NF1 was identified as the most closely related structure and the predicted three dimensional structure showed a typical α/β fold with alternating α-helices and β-strands, which is reminiscent to CRAL_TRIO domains. In addition to N and C-terminal strands, designated as β(N) and β(C), we defined the structural core of the BCH domain as three pairs of alternating α-helices and parallel β-strands, which enclose a hydrophobic cavity. The β(C) is parallel to the core β-sheet. The helices are amphipathic with hydrophobic residue facing inside and lining the hydrophobic cavity. The secondary structures also aligns well with the Jpred predictions [62]. The structures were predicted with high quality as judged by I-TASSER C-score (average: 1.1) and TM-values (average: 0.86). The structure of the BNIP-2-BCH domain as predicted by the ROBETTA server showed a good agreement with the I-TASSER prediction. However, because of major difference in the orientation of helix 3, the root mean squared deviation between the two structures was 3.4 Å. The helix3 was displaced by ∼10 Å in the ROBETTA predicted structure and thus the cavity appeared to be more open for ligand entry. Similarly, a kinking of corresponding helices has also been shown to cause an increase of the cavity volume for the yeast Sec14p structure [8]. The predicted structures for all three types of BCH groups exhibited a high similarity with an average root mean squared deviation of 1 Å.

### Three-dimensional structure highlighted important residue interactions

**Figure 4:**
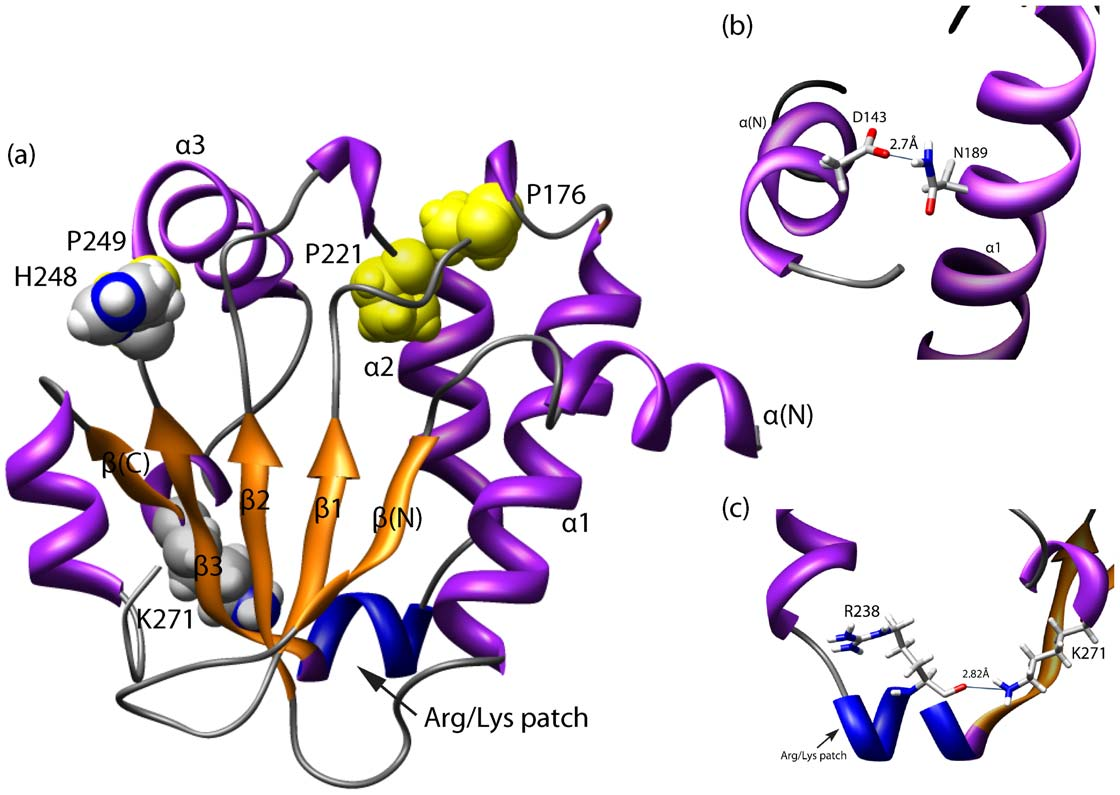
Predicted three dimensional structures of BCH domains. (a) A predicted three dimensional structure of the HsBNIP-2 BCH domain is displayed in this figure. The highly conserved proline residues are shown in yellow in a sphere representation. They are positioned in loops connecting the β-strands and α-helices. The patch of positively charged residues (called as Arg/Lys patch) is highlighted in blue color and the highly conserved residues H248, K271 are marked. (b) The side-chain of K271 comes in close contact with the backbone oxygen of R238 in the Arg/Lys patch (shown in zoomed box). This predicted interaction could provide added stability to the helical loop, which likely gates a lipid-binding cavity. (c) The side-chain of N189 from the Rho-binding region interacts with the side-chain of D143 of N-terminus α-helix (distance: 2.7 Å). This indicates that the N-terminus helix might be involved in Rho binding activity.

The predicted three-dimensional structures of BCH domains will help us to identify and better understand the functional importance of specific residues that are uniquely conserved within the BCH domain. It also revealed putative residue interactions, which might be involved in important functional aspects. The conserved HP-motif in BCH domains coincides with the NC-motif in NF1. The NC motif is proposed to cover the ligand entry site [10] or to be required for interactions with other proteins. Interestingly, it is located close to residue N277 (∼8 Å), which corresponds to the Cayman ataxia mutation S301R in human BNIP-H (Caytaxin) [27]. It is surprising to observe that all higher plant BCH domains have a conserved Arginine residue at this position. However, this residue does not appear to affect the lipid binding property of these proteins rather it might facilitate important protein-protein interactions. All the conserved Proline residues are found in loops that connect helices with strands and therefore might be critical for maintaining the overall domain conformation (e.g. sharp turns). Another conserved residue in BCH domains is Lys271 (HsBNIP-2 BCH numbering), which corresponds to K239 in yeast Sec14p and R221 in αTTP. A missense mutation at K239 in Sec14p has been reported to abolish PtIns transfer activity [55], [63] and a R221 mutation in αTTP is associated with a hereditary disorder known as AVED (ataxia with vitamin E deficiency) [6], [54], [64]. Being a part of a ‘hinge unit’, K239 might also contribute to controlling the movements of the helical gate [55]. In our modeled structure of BCH, this conserved Lysine (K271) forms a hydrogen bond with the backbone of the R238 residue (distance: ∼2.8 Å) which is part of the hallmark sequence motif of BCH domains (Figure 4), which forms a Arg/Lys patch at the base of hydrophobic cavity. Similarly, in Sec14p, K239 has the potential of forming a salt-bridge with E207. Although this interaction is not documented, it may allow this Lysine residue to contributing to a favorable conformation for binding a lipid ligand or to providing stability and rigidity to the conformation of the cavity. The patch of positively charged amino-acid residues might attract lipid head groups and thus BCH domains might interact with lipids containing an acidic head group.

BCH domains have also been shown to directly interact with small GTPase RhoA proteins and to be crucial for regulating the GAP activity in p50RhoGAP [19]. They also control RhoA activation through an interaction with regulator protein Lbc RhoGEF. This has been demonstrated for the BNIPXL protein [23]. In our predicted structure, the putative Rho binding motif extends from α1 to β2 (Figure 4a, 4b). The side-chain oxygen of Asn (N189) in this motif is located within the hydrogen bonding limit (distance: 2.74 Å) of Asp in the N-terminal helix (D143). Interestingly, this position is occupied by positively charged residues (N, K, R, H) in 85% of BCH sequences and in 80% in group-III BCH (RhoGAP group) sequences. This observation points to a possible role of the N-terminal helix in regulating Rho binding activity. No such conservation has been observed for the corresponding positions in CRAL_TRIO domains. However, their N-termini also contain conserved positively charged amino-acid residues (81%).

## Discussion

**Figure 5:**
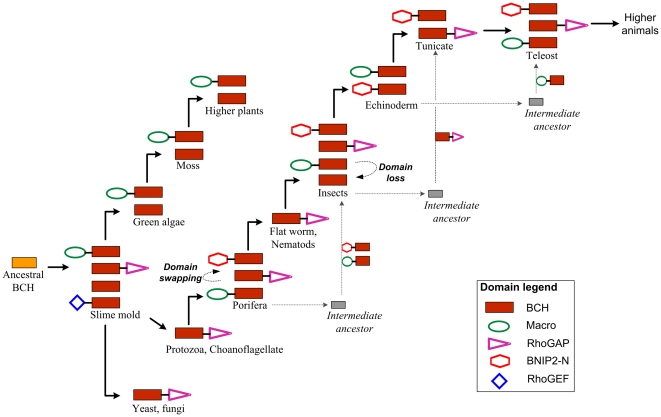
Diversification of BCH domains. BCH domains evolved from a CRAL_TRIO like ancestor and diverged into three subgroups with distinct protein domain architectures. This figure displays the predicted path of divergence for each of the three BCH subgroups. Plant and animal BCH subgroups diverged independently. The ‘BCH-only’ subgroups of plants and insects also descended from different ancestors. This is evident from their phylogenetic clustering and their gene-structure. Group-I BCH proteins/genes might have arisen after domain swapping events. Nematodes have only group-III BCH domain proteins, which are associated with a RhoGAP domain. The divergence into three distinct subgroups in the following lineages is therefore the result of either another domain swapping event or an unknown intermediate ancestor.

Similar structural features within proteins often indicate a common evolutionary origin. Subsequently, changes and a divergence of the primary sequence lead to functional specializations. Unlike conventional CRAL_TRIO domains, BCH domains are usually implicated in controlling cell dynamics by modulating the activity of small GTPases and their regulator proteins. These activities appear to be independent of interactions with hydrophobic ligands. Here we show that BCH domain-containing proteins diverged from the CRAL_TRIO gene family and acquired unique sequence features, which might contribute to their ability of binding ligands other than lipids. This divergence occurred during evolution as early as the appearance of protists. Among the three BCH subgroups, group-III (RhoGAP-type) appeared first and all three groups further diverged by intron insertion, domain swapping and gene duplication events. A possible evolutionary path for all three subgroups of BCH domain genes is depicted in Figure 5. This figure highlights the important events, which we postulate occurred during the divergence of BCH domains from their ancestor in various groups of organisms. Understanding the point of divergence and unique features of BCH and CRAL_TRIO domains is particularly interesting in the light of observation that BCH domains directly bind small GTPases and their regulators GAPs and GEFs, to modulate signaling cascades. The hydrophobic nature of these domains may also contribute to these interactions through homophilic or heterophilic associations as shown in BNIP-2 protein [56]. In addition, various long chain hydrophobic residues, which form a potential binding cavity within the BCH domains, have been conserved. This raises the possibility of interactions with hydrophobic ligands. Further structural and functional studies need to be carried in order to understand the potential implications for small GTPase signalling mediated by BCH domains. This will also lead to a better understanding of the functional roles involving other, previously uncharacterized ‘BCH-like’ domain-containing proteins.

## Supporting Information

Figure S1 **Taxonomic distribution of BCH domains across species.** The numbers shown in this figure do not include alternative splice protein isoforms. Grey bars indicate the number of genomes represented in that group, while blue bars indicate the number of BCH domain-containing proteins, which were identified by database searches. Overall, mammalian genomes encode the highest number of BCH domain proteins, while lower organisms have only one or two BCH genes.
(TIFF)

Figure S2 **Neighbor-Joining trees of BCH groups.** The group-I, IIA, IIB and III BCH subgroups have 68, 30, 33 and 41 members respectively. In order to maintain clarity, the percent bootstrap values are shown for all branches except smaller branches. Accession numbers are displayed only if more than one BCH domain sequence was identified from one organism. The protein accession numbers for others can be found in the Table S1. The abbreviations used are as follows; Dr: *Danio rerio*, Tn: *Tetraodon nigroviridis*, Ci: *Ciona intestinalis*, Dd: *Dictyostelium discoideum*, At: *Arabidopsis thaliana*, Rc: *Ricinus communis*, Pt: *Populus trichocarpa*, Gm: *Glycine max*, Mt: *Medicago truncatula*, Ps: *Picea sitchensis*, Zm: *Zea mays*, Os: *Oryza sativa*.
(DOC)

Figure S3 **Alternative splicing within BCH domains.** We investigated the alternative splicing at the C-termini of all three major groups of BCH domain proteins. Most of these isoforms were identified in mammalian species and only one from *Xenopus* and *Danio* suggesting functional complexity in higher organisms. A number of isoforms were identified in group-II BCH domains of *Pan troglodytes* (Chimpanzee). The functional implication of splice isoforms has been demonstrated for the BCH domain of the *Hs*BNIP-S protein. The isoform BNIP-Sα containing a complete BCH domain mediates the pro-apoptotic effect, whereas the alternatively spliced isoform BNIP-Sβ is lacking such a domain (having only half of sequence of BCH domain) and functionality [14].
(DOC)

Table S1 **The table contains NCBI Accessions of 175 BCH domain-containing proteins and 98 other proteins representing the BCH-like and CRAL_TRIO groups of the Sec14 superfamily.**
(XLS)

Table S2 **The file contains multiple sequence alignments of 175 BCH domains and 98 BCH-like and CRAL_TRIO domains.**
(DOC)
