# Supplementary figures and images for: Cross-Species Analyses Identify the BNIP-2 and Cdc42GAP Homology (BCH) Domain as a Distinct Functional Subclass of the CRAL_TRIO/Sec14 Superfamily
Source: PLoS One. 2012 Mar 27;7(3):e33863. doi: 10.1371/journal.pone.0033863 (PMC3313917; doi:10.1371/journal.pone.0033863)

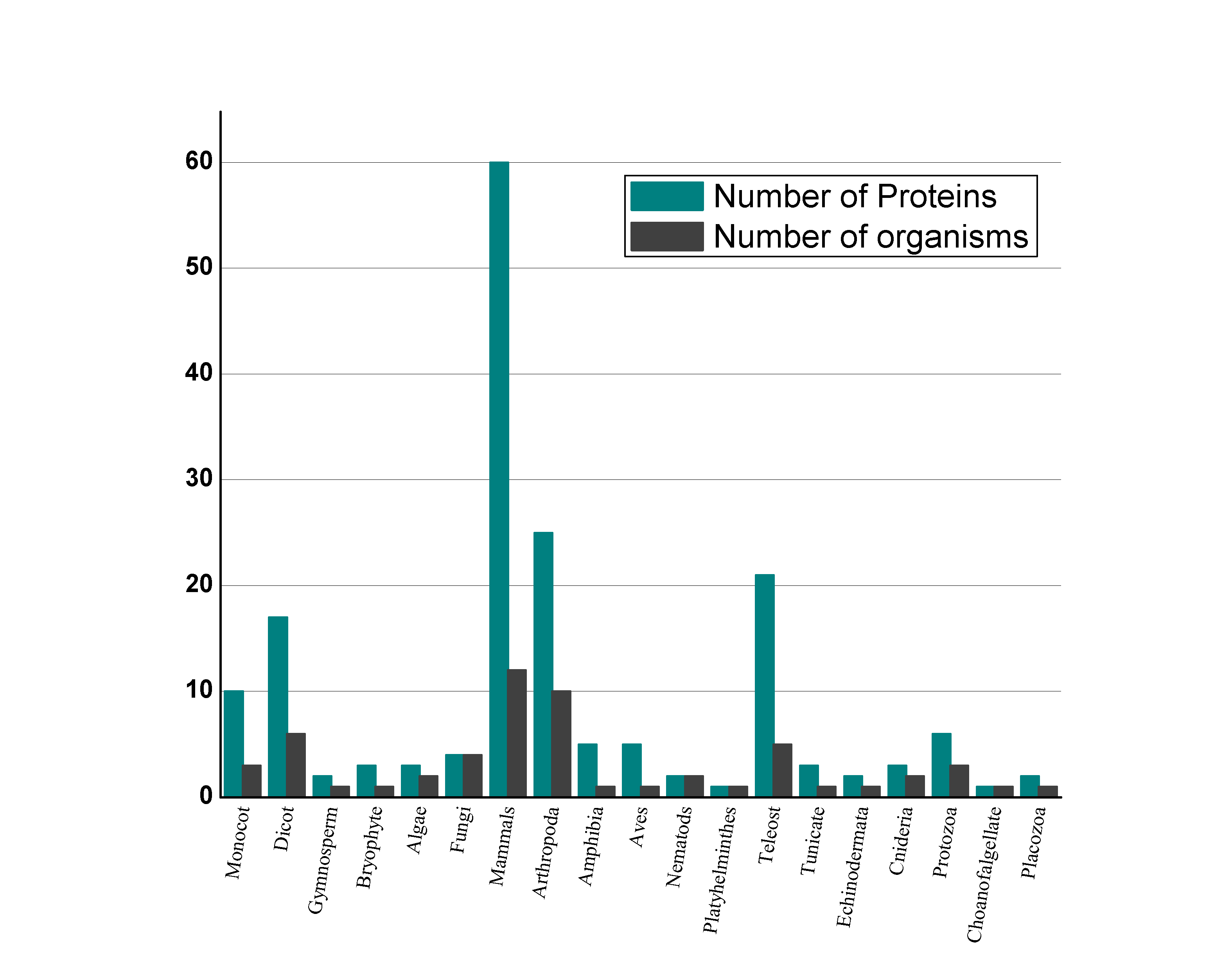

Supplement: Figure S1 — Taxonomic distribution of BCH domains across species. The numbers shown in this figure do not include alternative splice protein isoforms. Grey bars indicate the number of genomes represented in that group, while blue bars indicate the number of BCH domain-containing proteins, which were identified by database searches. Overall, mammalian genomes encode the highest number of BCH domain proteins, while lower organisms have only one or two BCH genes. (TIFF) [file pone.0033863.s001.tiff]
