# Supplementary material for: Cross-Species Analyses Identify the BNIP-2 and Cdc42GAP Homology (BCH) Domain as a Distinct Functional Subclass of the CRAL_TRIO/Sec14 Superfamily
Source: PLoS One. 2012 Mar 27;7(3):e33863. doi: 10.1371/journal.pone.0033863 (PMC3313917; doi:10.1371/journal.pone.0033863)
Supplement: Table S2 — The file contains multiple sequence alignments of 175 BCH domains and 98 BCH-like and CRAL_TRIO domains. (DOC) [file pone.0033863.s005.doc]

CLUSTAL 2.0.8 multiple sequence alignment

XP_001173154_Pan_BCH IEPYKKVISHGG-----------YYGDGLNAIVVFAVCFMPES-------

XP_544705_Canis_BCH IEPYKKVISHGG-----------YYGDGLNAIVVFAVCFMPES-------

XP_001374074_Monodelphis_BCH IEPYKKVISHGG-----------YYGDGLNAIVVFAVCFMPES-------

NP_001008239_Mus_BCH IEPYKKVISHGG-----------YYGDGLNAIVVFAVCFMPES-------

EDL84204_Rattus_BCH IEPYKKVISHGG-----------YYGDGLNAIVVFAVCFMPES-------

XP_001100508_Macaca_BCH IEPYKKVISHGG-----------YYGDGLNAIVVFAVCFMPES-------

NP_004321_Homo_BCH IEPYKKVISHGG-----------YYGDGLNAIVVFAVCFMPES-------

XP_001510218_Ornithorhynchus_B IEPYKKVISHGG-----------YYGDGLNAIVVFAVCFMPES-------

XP_413765_Gallus_BCH IEPYKKVISHGG-----------YYGDGLNAIVVFAVCFMPES-------

XP_002195589_Taeniopygia_BCH IEPYKKVISHGG-----------YYGDGLNAIVVFAVCFMPES-------

NP_001088131_Xenopus_BCH IEPYKKVISHGG-----------YYGDGLNAIIVFAVCFMPDS-------

CAF98314_Tetraodon_BCH IEPYKRVISHGG-----------YYGDGLNAIIVFAVCFMPES-------

NP_957512_Danio_BCH IEPYKKVISHGG-----------YYGDGLNAIIVFAVCFMPES-------

XP_001366186_Monodelphis_BCH IEPYKRVISHGG-----------YYGDGLNAIIVFAACFLPDS-------

XP_001916938_Equus_BCH IEPYRRVISHGG-----------YYGDGLNAIIVFAACFLPDS-------

NP_056040_Homo_BCH IEPYRRVISHGG-----------YYGDGLNAIIVFAACFLPDS-------

XP_001146885_Pan_BCH IEPYRRVISHGG-----------YYGDGLNAIIVFAACFLPDS-------

NP_001126916_Pongo_abe_BCH IEPYRRVISHGG-----------YYGDGLNAIIVFAACFLPDS-------

NP_001098852_Bos_BCH IEPYRRVISHGG-----------YYGDGLNAIIVFAACFLPDS-------

NP_851993_Mus_BCH IEPYRRVISHGG-----------YYGDGLNAIIVFAACFLPDS-------

Q5BJR4_Rattus_BCH IEPYRRVISHGG-----------YYGDGLNAIIVFAACFLPDS-------

XP_002187337_Taeniopygia_BCH IEPYKKVISHGG-----------YYGDGLNAIIVFAACFLPDS-------

XP_001231886_Gallus_BCH IEPYKKVISHGG-----------YYGDGLNAIIVFAACFLPDS-------

XP_850604_Canis_BCH IEPYRRVISHGGDS--------GYYGDGLNAIIVFAACFLPDS-------

XP_001515884_Ornithorhynchus_B IEPYKRVIAHGG-----------YYGDGLNAIIVFAACFLPDS-------

CAG08427_Tetraodon_BCH IEPYKRVISHGG-----------YYTER-NAIIVFSACFLPDS-------

NP_001104624_Danio_BCH IEPYQKVISHGG-----------YYSNGANAIIVFAACFLPDS-------

CAF97194_Tetraodon_BCH IEPYMRVISHGG-----------YYGNEVNAIIVFAACFLPDS-------

NP_001133314_Salmo_BCH IEPYQRVISHGG-----------YYGNGVNAILVFAACFLPDS-------

XP_001374042_Monodelphis_BCH IKPYMKVVTHG-----------GYYGEGLNAIIVFAACYLPDS-------

XP_001511480_Ornithorhynchus_B IKPYMKVVTHG-----------GYYGEGLNAIIVFAACYLPDS-------

XP_542168_Canis_BCH IRPYMRVVTHG-----------GYYGEGLNAIIVFAACFLPDS-------

NP_001125042_Pongo_abe_BCH IRPYMKVVTHG-----------GYYGEGLNAIIVFAACFLPDS-------

NP_001075898_Bos_BCH IRPYMRVVTHG-----------GYYGEGLNAIIVFAACFLPDS-------

NP_848777_Mus_BCH IRPYMKVVTHG-----------GYYGEGLNAIIVFAACFLPDS-------

NP_001035280_Rattus_BCH IRPYMKVVTHG-----------GYYGEGLNAIIVFAACFLPDS-------

XP_524457_Pan_BCH IRPYMKVVTHG-----------GYYGEGLNAIIVFAACFLPDS-------

BAC03859_Homo_BCH IRPYMKVVTHG-----------GYYGEGLNAIIVFAACFLPDS-------

XP_001493463_Equus_BCH IRPYMRVVTHG-----------GYYGEGLNAIIVFAACFLPDS-------

XP_418214_Gallus_BCH IKPYMRVVTHG-----------GYYGEGLNAIIVFAACYLPDS-------

XP_002195707_Taeniopygia_BCH IKPYMRVVTHG-----------GYYGEGLNAIIVFAACYLPDS-------

CAF91924_Tetraodon_BCH IRPYLRVVTHG-----------GYYGEGLNAIIVFAACHLPDS-------

XP_001343600_Danio_BCH IRPYLRVVTHGGAAPHFHLLQTGYYGEGLNAIIVFAACYLPDS-------

CAG08009_Tetraodon_BCH SAPHCNLLPSG------------YYGEGLNAIIVFSACYLPDS-------

CAN87813_Danio_BCH IRPYLRVISHGG-----------YYGEGLNAIIVFTACYLPDS-------

XP_001170701_Pan_BCH IEPYKKVLSHGG-----------YHGDGLNAVILFASCYLPRS-------

NP_612122_Homo_BCH IEPYKKVLSHGG-----------YHGDGLNAVILFASCYLPRS-------

XP_001106978_Macaca_BCH IEPYKKVLSHGG-----------YHGDGLNAVILFASCYLPRS-------

XP_001491310_Equus_BCH IEPYKKVLSHGG-----------YHGDGLNAVILFASCYLPRS-------

XP_001928046_Sus_BCH IEPYKKVLSHGG-----------YHGDGLNAVILFASCYLPRS-------

NP_001073089_Bos_BCH IEPYKKVLSHGG-----------YHGDGLNAVILFASCYLPRS-------

EDL38792_Mus_BCH IEPYKKVLSHGG-----------YHGDGLNAVILFASCYLPRS-------

NP_001121659_Rattus_BCH IEPYRKVLSHGG-----------YHGDGLNAVILFASCYLPRS-------

XP_540308_Canis_BCH IEHYKKVLSHGG-----------YHGEGLNAVIVFASCYLPSS-------

XP_001371249_Monodelphis_BCH IEPYKKVLSHGG-----------YHGDGLNAVILFASCYLPRS-------

NP_001086594_Xenopus_BCH IEPYKQVISHGG-----------YYGEGLNAVIVFASCYLPED-------

NP_001121866_Danio_BCH LEPYLQVLSHGG-----------YYGDGSTAIIMFTSCYLPEN-------

XP_002127893_Ciona_BCH VTPYRQILSHGG-----------YYGEGLNAIVVFSACYLPDT-------

EEC09901_Ixodes_BCH IEPYKKVLSHGG----------YFGEERH-AIIVFSACYLPDR-------

XP_001947520_Acyrthosiphon_BCH IEPYKCVLSHGG----------YMTTGSHNAIIVFSACYLPHR-------

EEB18677_Pediculus_BCH LEPYKRVISHGG----------YLSKESSHAIVIFSACFLPDR-------

XP_315572_Anopheles_BCH IEPYKCVLSHGG----------YLQSGGHNAIVVFSACHLPDR-------

XP_001648558_Aedes_BCH IEPFKRVLSHGG----------YLQSGGHNAIVVFSACHLPDR-------

ABY20545_Drosophila_mel_BCH IEPYKRVLSHGG----------YLKSGGQNAIVIFCACHLPDR-------

XP_974968_Tribolium_BCH IEPYKRVLSHGG----------YLRAGGHTAIVIFSACYLPDR-------

XP_783795_Strongylocentrotus_B INPYKKVLSHGG----------YYG-EGLNAIIVFASCYLPEK-------

XP_001626140_Nematostella_BCH LEPYMKVITHGG-----------FHCPERAVIVVIAACYLPSK-------

XP_002126187_Ciona_BCH IQPYKRAISHGG------------FYKDKSTIVEIIGTYLPEN-------

XP_508398_Pan_BCH DDPYYDIARHQIVEV------AGD-DKYGRKIIVFSACRMP---------

CAM14521_Mus_BCH DDPYYDIARHQIVEV------AGD-DKYGRKIIVFSACRMP---------

NP_001124902_Pongo_abe_BCH DDPYYDIARHQIVEV------AGD-DKYGRKIIVFSACRMP---------

XP_001101907_Macaca_BCH DDPYYDIARHQIVEV------AGD-DKYGRKIIVFSACRMP---------

BAG60756_Homo_BCH DDPYYDIARHQIVEV------AGD-DKYGRKIIVFSACRMP---------

NP_001101217_Rattus_BCH DDPYYDIARHQIVEV------AGD-DKYGRKIIVFSACRMP---------

XP_001490021_Equus_BCH DDPYYDIARHQIVEV------AGD-DKYGRKIIVFSACRMP---------

XP_871999_Bos_BCH DDPYYDIARHQIVEV------AGD-DKYGRKIIVFSACRMP---------

XP_001369707_Monodelphis_BCH DDPYYDIARHQIVEV------AGD-DKFGRKIIVFSACRMP---------

XP_426422_Gallus_BCH DDPYYDIARHHIVEV------AGD-DKYGRKVILFSACRMP---------

XP_002198819_Taeniopygia_BCH DDPYYDIARHHIVEV------AGD-DKYGRKVILFSACRMP---------

NP_001086509_Xenopus_BCH DDPYYDIARHQIVEV------AGD-DKYGRKIVVFSACRLP---------

NP_001017781_Danio_BCH DDPFYDIARHQIVEV------AGD-DNFGRKVIVFNACRMP---------

EDL04450_Mus_BCH SHPFYDVARHGILQV------AGD-DRQGRRIFTFSCCRLP---------

NP_001004242_Rattus_BCH SHPFYDVARHGILQV------AGD-DRQGRRIFTFSCCRLP---------

CAQ06715_Homo_BCH SHPFYDVARHGILQV------AGD-DRFGRRVVTFSCCRMP---------

XP_416463_Gallus_BCH NHPYYDVARHGIIQL------AGD-DNSGRKVITFSCCRMP---------

NP_001085153_Xenopus_BCH GHPYYDIARHGIIHV------VGD-DNSGRKVITFSCCRLP---------

EEB11750_Pediculus_BCH DEDFSDIEKYGIVEV------AGD-DPYGRKVIVVSACKLP---------

XP_001950301_Acyrthosiphon_BCH DEDFSDVAECGVVDV------VGD-DAVGRKIIVVSACKLP---------

XP_001604462_Nasonia_BCH EEDYHDIARLGIVEV------VGD-DSAGRKVIVVSACKLPPV-------

XP_624226_Apis_BCH EEDYLDISRYGIVEV------VGD-DNAGRKVIVVSACKLPPV-------

XP_002121397_Ciona_BCH KEEFADIAKYGIIEV------SGV-DTSGRPVIVVSASKLP---------

XP_970580_Tribolium_BCH TRKFSEIDKHKIVDV------KGD-DSAGRKIIVVYAHRLP---------

XP_002160831_Hydra_BCH QDKYADIAKLKIFHV------AGD-DLTGRPVIAFSACRLP---------

NP_001022390_Caenorhabditis_el EDLFNDISAHEIIQV------IADGDRVGRPIVVVYAYRLP---------

XP_001893352_Brugia_BCH AEDFSNISQHEIVNV------IADGDWVGRPIVVIYAYRLP---------

XP_002117739_Trichoplax_BCH CGGYAEVTVTNFLHLFYLH--VGD-DRNSRKLILFYSCRLP---------

XP_002572184_Schistosoma_BCH DSDYPDISRLGVLQG------AGD-DKLGRKIIVFSACRLP---------

EEC10256_Ixodes_BCH REDFDDIHRYGIVEI------AGD-DAYGRNVIVISACRLP---------

XP_001746114_Monosiga_BCH PLDYSEIEKYKILDT------AGT-DRLGRHVFVFYAAHMP---------

XP_001835505_Coprinopsis_BCH ---MGRP--------------------ESR--VVLNASALP---------

XP_001876797_Laccaria_BCH NGGYGESEMVQEVLSKMIF--QAGVDFETRPMVVLNASVLP---------

XP_760803_Ustilago_BCH TEAIARIEKLDSYVRRIVF--QAGLDYETRPMVVLAACCLP---------

XP_001843366_Culex_BCH VEPDENAIRKEFKRRKF--IEFIGTDKHGQPIIAIYACSLP---------

XP_001659702_Aedes_BCH MEPDENAIRKEFKRRKF--IEFIGTDKHGQPIIAIYACSLP---------

NP_648552_Drosophila_mel_BCH -EQSENFQTPRNK------CDFLGTDKQGRHIFGIYASRFP---------

XP_001182784_Strongylocentrotu IAAHRCIYATG-------------HDIHGRPIIAFVARNFPAH-------

XP_001622394_Nematostella_BCH FSRQKILYQSG-------------VDFLGRPVVVFVARHFTAQ-------

XP_396878_Apis_BCH VSGIGCLYQSG-------------VDRQGRPVVVFVGKWFPAT-------

XP_001815509_Tribolium_BCH VSGIGCLYQSG-------------VDRLGRPVVVFIGKWFPFN-------

NP_724599_Drosophila_mel_BCH VSGIGCLYQSG-------------VDRLGRPVIVFCGKWFPAQ-------

NP_724597_Drosophila_mel_BCH VSGIGCLYQSG-------------VDRLGRPVIVFCGKWFPAQ-------

XP_001867337_Culex_BCH VSGIGCLYQSG-------------VDRLGRPVIVFCGKWFPAH-------

XP_001688494_Anopheles_BCH VSGIGCLYQSG-------------VDRLGRPVVVFCGKWFPAH-------

XP_001660566_Aedes_BCH VSGIGCLYQSG-------------VDRLGRPVVVFCGKWFPAQ-------

EEB16863_Pediculus_BCH VSGIGCLYQCG-------------VDIYGRPVIIFVGKWFKFK-------

ACO15636_Caligus_BCH IDYSGYIYRSG-------------TDKDGRPIIVFIGKWFRAN-------

XP_002112464_Trichoplax_BCH IIKLKALYKSG-------------VDQYGRSVIVFIGNNFPAH-------

XP_423602_Gallus_BCH IASLKALYQTG-------------VDNCGRTVMVVVGRNIPVT-------

XP_002197809_Taeniopygia_BCH IASLKALYQTG-------------VDNCGRTVMVVVGRNIPVT-------

XP_001144527_Pan_BCH IASLKALYQTG-------------VDNCGRTVMVVVGRNIPVT-------

NP_060156_Homo_BCH IASLKALYQTG-------------VDNCGRTVMVVVGRNIPVT-------

XP_001500874_Equus_BCH IASLKALYQTG-------------VDNCGRTVMVVVGRNIPVT-------

NP_001039478_Bos_BCH IASLKALYQTG-------------VDNCGRTVMVVVGRNIPVT-------

XP_533021_Canis_BCH IASLKALYQTG-------------VDNCGRTVMVVVGRNIPVT-------

XP_001365992_Monodelphis_BCH IASLKALYQTG-------------VDNCGRTVMVVVGRNIPVT-------

NP_001013219_Rattus_BCH IASLKALYQTG-------------VDNCGRTVMVVVGRNIPVT-------

NP_034399_Mus_BCH IASLKALYQTG-------------VDNCGRTVMVVVGRNIPVT-------

XP_001104476_Macaca_BCH IASLKALYQTG-------------VDNCGRTVMVVVGRNIPVT-------

NP_001004563_Danio_BCH IAALKALYQTG-------------VDLCGRTVMVVVGRNIPVM-------

CAF93717_Tetraodon_BCH IAALKALYQTG-------------VDMCGRTVMVVVGRNIPVT-------

ACO13754_Esox_BCH IAALKALYQTG-------------VDIYGRTVMVLVGRNVPVN-------

NP_001088345_Xenopus_BCH IAALKALYQSG-------------VDNCGRSVMVVVGRNIPVL-------

XP_001512063_Ornithorhynchus_B IASLKALYQTG-------------VDNCGRTVMVVVGRNIPVT-------

XP_001776374_Physcomitrella_BC VAEMKILYRGG-------------VDVDGRPIMVVVGAHFLLR-------

XP_001759372_physcomitrella_BC VAEMKILYRGG-------------VDVDGRPIMVVVGAHFLLR-------

EEF01185_Populus_BCH IAEMKIVYRGG-------------VDSEGHPVMVVVGAHFLLR-------

EEE89642_Populus_BCH IAEMKIVYRGG-------------VDSEGRPVMVVVGAHFLLR-------

EEF38953_Ricinus_BCH IAEMKIVYRGG-------------VDSEGRPVMVVVGAHFLLR-------

NP_564960_Arabidopsis_BCH IAEMKIVYRGG-------------VDTEGHPVMVVVGAHFLLR-------

XP_002467159_Sorghum_BCH IAEMKIIYRGG-------------VDIEGRPVMVVVGAHFLLR-------

ACG47767_Zea_BCH IAEMKIIYRGG-------------VDIEGRPVMVVVGAHFLLR-------

NP_001064706_Oryza_BCH IAEMKIIYRGG-------------VDSEGRPVMVVVGAHFLLR-------

XP_002465844_Sorghum_BCH VAEMKIIYRGG-------------VDSEGHPVMVVVGAHFLLR-------

ACG45534_Zea_BCH VAEMKIIYRGG-------------VDSEGRPVMVIVGAHFLLR-------

ABK24296_Picea_BCH IAEMKIVYRGG-------------VDNEGRPVMVVVGAHFLLR-------

EEE58332_Oryza_BCH SGLTRSSYRGG-------------VDSEGRPVMVVVGAHFLLR-------

EEE91516_Populus_BCH LDLLQFFTLQG-------------SDKSGNRVFRIVGKYFPAQ-------

EEF30886_Ricinus_BCH LDLLQFFRLQG-------------SDKSGNRIFRVIGKYFPAQ-------

ACU17631_Glycine_BCH LDQLQFLRLQG-------------SDKNSNRILRIVGKYFPAT-------

ABD28324_Medicago_BCH LEFLQFFTLQG-------------TDKNGTRILRIIGKHYPAT-------

ABR17649_Picea_BCH LDQLQILELQG-------------KDRAGRRILRIVGKFFPAP-------

XP_001754720_Physcomitrella_BC LEPLQILDLQG-------------VDVLGRQVVRIVGKHLPARGLELSLM

NP_001047687_Oryza_BCH LERSRAITLNG-------------RDKRGRALVRIVGKYFP---------

ACG26273_Zea_BCH LERSRAITVQG-------------GDKRGRAVVRIVGKYFP---------

ACG34784_Zea_BCH LERSRAITLQG-------------RDRKGRAVVRIVGNYFP---------

BAF15465_Oryza_BCH LEKKRVITVQG-------------RDKAGRPIVRIVGKNFP---------

EEF31637_Ricinus_BCH IEKLEIFKILG-------------RDKHGHNILRIIGKFFP---------

NP_195300_Arabidopsis_BCH IEKLEIFKIHG-------------RDKRGRKILRIIGKFFP---------

ACJ85167_Medicago_BCH LEKLEVFKIKG-------------RDKHGRKILRIIGKFFP---------

ACU14441_Glycine_BCH LEKLEVFKIKG-------------RDKHGRKILRIIAKFFP---------

EEE75109_Populus_BCH VEKLEIFKFQG-------------RDKNGHKVLRIIGKFLS---------

EEE93960_Populus_BCH LEELGIFKIQG-------------RDKGGRKVLLITGKHFPGKDNMQFTL

EEE81211_Populus_BCH LEKLGVFKIQG-------------RDKHGRKVLLIIGKLFP---------

EEF52804_Ricinus_BCH IDKLEVFKIQG-------------RDKRGRKVLRIVGKLFP---------

CAN69115_Vitis_BCH MEKLQIFKIQG-------------TDRHGRKVLVIIGKYFP---------

XP_001699293_Chlamydomonas_BCH LDQLGFLSVPGG------------RDKEGRQVVMVAAKNYP---------

XP_002168194_Cnidarian_NF1 IKSNQLFYGDGK-------------SKAGRHVFYYIARRYMNDM------

XP_001744997_Coano_NF1 IEARNLFYRRGT-------------TEDHRPVFYYIARRYMTGE------

AAB59558_human_NF1 LKTLSIFYQAGT-------------SKAGNPIFYYVARRFKTGQ------

XP_692937.3_Danio_NF1 LKTLHIFYQAGT-------------SKNGNPVFYYVTRRFKTGQ------

XP_002587546_lancelet_NF1 IKSLNIFYQAGN-------------SKAGNPVFYYIARRYKSGQ------

AAB58976_Drosophila_NF1 LKSMNIFYQAGT-------------SKSGYPVFYYIARRYKIGE------

XP_001196417_Echinoderm_NF1 LKSLNIFYQDGI-------------SRAGNPVFYYVARKYKCSE------

XP_002576216_tremtoda_NF1 LKNVNAFYQAGT-------------SRLGNPVFYYIARRYKSREY-----

XP_002115170_placozoa_NF1 IKDANFFYLEGK-------------SRKKYPVFYYICNNLCCEQ------

XP_002121778_ciona_NF1 VKGLNIFYQFGR-------------SKMGYPVFYYVARRYVLHN------

NP_013796_Saccharomyces_CRALTR PQYYHKTDKDGRP-------------VYFEELGAVNLHEMNK--------

NP_012832_Saccharomyces_CRALTR PQYYHHVDKDGRP-------------LYFEELGGINLKKMYK--------

NP_195629_Arabidopsis_CRALTRIO PQGYHGVDKEGRP-------------IYIERLGQVDATKLMK--------

NP_036561_Homo_CRALTRIO SGGMCGYDLDGCP-------------VWYDIIGPLDAKGLLF--------

NP_777635_Homo_CRALTRIO PGGLCGYDRDGCP-------------VWYDIIGPLDPKGLLF--------

NP_001091144_Xenopus_CRALTRIO SGGLCGHDRENSP-------------IWYDVVGPLDPKGLLF--------

NP_001093463_Danio_CRALTRIO SGGMCGHDREGSP-------------VWYDVIGPLDPKGLMH--------

NP_777637_Homo_CRALTRIO SGGLCGYDYEGCP-------------VYFNIIGSLDPKGLLL--------

NP_612042.3_Drosophila_CRALTRI PYGLMGYDNEGSP-------------VLVCPFANFDMWGMMH--------

NP_001087870_Xenopus_CRALTRIO AGGWHHHDKDGRP-------------LYVLRLGQMDTKGLVR--------

NP_001085706_Xenopus_CRALTRIO AGGWHHHDRDGRP-------------LYLLRLGQMDTKGLVR--------

NP_001137470_Homo_CRALTRIO AGGWHHHDKDGRP-------------LYVLRLGQMDTKGLVR--------

NP_957392_Danio_CRALTRIO TGGWHHHDKDGRP-------------LYILRLGQMDTKGLVR--------

NP_055507_Homo_CRALTRIO AGGWHYQDIDGRP-------------LYILRLGQMDTKGLMK--------

NP_001040875_Caenorhabditis_CR PGCWHNSDKAGRP-------------MYILRFGQLDTKGMLR--------

NP_609028_Drosophila_CRALTRIO PGGWHHLDKDGRP-------------VYILRLGHMDVKGLLK--------

NP_506408_Caenorhabditis_CRALT KTGLTGESGIIPN-TI----------VNIEQTGSNDYWGMLH--------

NP_510554_Caenorhabditis_CRALT KYGITGPSGHMDN-VI----------VNIEQCGKTDYTGMME--------

NP_495168_Caenorhabditis_CRALT PLGLVGETGKDN--QL----------LVIECAGRIDLMGILK--------

NP_505746_Caenorhabditis_CRALT PIDIIGPQRKEDGDRL----------VVVDRAGRIDVSGLMK--------

CAB78040_Arabidopsis_CRALTRIO VVFMQGQDKENHP-------------VCYNVYGEFQNKDLYQK-------

NP_177360_Arabidopsis_CRALTRIO MVFAHGVDKEGHV-------------VIYSSYGEFQNKEL----------

NP_013484_Saccharomyces_CRALTR IQGY---DNDMRP------------------VILVRPRLHHSS-------

XP_643391_Dict_CRALTRIO FHGV---DKGGRP------------------VCIVKTSRHDSY-------

NP_728794_Drosophila_CRALTRIO LRHR---DCIGRP------------------VIYIPAKNHSS--------

NP_014168_Saccharomyces_CRALTR ILGY---ENDARP------------------ILYLKPGRQNT--------

NP_014135_Saccharomyces_CRALTR ILGF---DNAKRP------------------LYYMKNGRQNT--------

XP_629043_dict_CRALTRIO VFG----KSHGRS------------------VIYLRPVRENT--------

NP_195382_Arabidopsis_CRALTRIO RASFH--DRQGRV------------------VLIMRPAMQNS--------

BAH19871_Arabidopsis_CRALTRIO RANCT--DKYGRT------------------VLVMRPSCQNT--------

NP_001088147_Xenopus_CRALTRIO LHGY---DKEGNK------------------LLWLKVKLHVRD-------

NP_001088926_Xenopus_CRALTRIO LHGY---DKEGNK------------------LLWLRVKLHVRD-------

NP_689794_Homo_CRALTRIO LHGY---DKEGNK------------------LFWIRVKYHVKD-------

NP_001007294_Danio_CRALTRIO LHGY---DKEGNK------------------LFWFKVKLHIKD-------

NP_729113_Drosophila_CRALTRIO VHNK---DRDGKP------------------LLILTIKKHSKS-------

NP_000317_Homo_CRALTRIO PGVLSSRDKYGRV-----------------VMLFNIE-NWQSQ-------

NP_001080386_Xenopus_CRALTRIO PGILTSRDKNGRV-----------------ILLFNIE-NWDYE-------

NP_956999_Danio_CRALTRIO PRILSTRDKNGRV-----------------VLLFNID-NWDLE-------

NP_775790_Homo_CRALTRIO PGVLENRDHYGRK-----------------ILLLFAA-NWDQS-------

NP_001088883_Xenopus_CRALTRIO PGVLENRDHYGRK-----------------ILLLFAA-NWDQS-------

NP_001010852_Homo_CRALTRIO PGGLANLDHYGRK-----------------ILVLFAA-NWDQS-------

NP_001020716_Danio_CRALTRIO PGVLSNLDRYGRK-----------------ILVLFAA-NWDQS-------

NP_001034288_Homo_CRALTRIO LTVLPHTDPRGCH-----------------VVCIRPD-RWIPS-------

NP_997755_Danio_CRALTRIO LTVLPRPDPQGRY-----------------ILCLRPG-KWKPN-------

NP_000361_Homo_CRALTRIO HGVLRSRDPTGSK-----------------VLIYRIA-HWDPK-------

NP_724195_Drosophila_CRALTRIO LTVTPYRDQHGHR-----------------ILIYRFG-LWRPN-------

NP_609120_Drosophila_CRALTRIO LNLLPQRDQHGRR-----------------LLVLEAGKKWKPS-------

NP_001079459_Xenopus_CRALTRIO FTVLPGRDENGAA-----------------LALFTAR-LHRPD-------

NP_001081203_Xenopus_CRALTRIO FTVLPGRDANGAA-----------------VALFTAR-LHRPD-------

NP_572576_Drosophila_CRALTRIO FTILPARTSSGAA-----------------IALFTAN-RHSPL-------

NP_002824_Homo_CRALTRIO FTILNVRDPTGAS-----------------IALFTAR-LHHPH-------

NP_724813_Drosophila_CRALTRIO VRLPKPWGTDGPR-----------------LQLTNYE-KFDPK-------

NP_610507_Drosophila_CRALTRIO ATLPKPIGPGGPR-----------------IHYTRMG-HIEPS-------

NP_001021496_caenorhabditis_Rh VLRDGIAVLPGGR------------CRAGQAVIVCP-SRE--QP------

XP_001891671_brugia_RhoGEF VLRDRVALLPGAR------------DRNGRAVIIFP-ARENTTP------

NP_009049_Homo_RhoGEF ILKEKVAYLSGGR------------DKRGGPILTFPARSNHDR-------

NP_001097996_danio_RhoGEF ILKEKVAFLSGGR------------DKRGGPVLTFPSRTNHDR-------

NP_001019831_Homo_RhoGEF ILKEKVAFVSGGR------------DKRGGPILTFPARSNHDR-------

XP_002598613_branchiostoma_Rho ILREKIAFLSGGR------------DMRGGPVLTFPQRNNPEKA------

NP_651960_drosophila_RhoGEF LLQERVVFLTGGR------------DRRGGPLLCFPATPRRDR-------

XP_002578747_schistosoma_RhoGE LLKKKIALLPGGR------------SRRGGPLLCFPSNSRADE-------

XP_001635815_nematostella_RhoG ------------I------------DRRGGRIITLPAHTFQGEQ------

NP_835224.3_Homo_RhoGEF ILKKKLAFLSGGK------------DRRSGLILTIPLCLEQTN-------

NP_001085886_xenopus_RhoGEF ILKKKLAFLSGGK------------DRRSGLILTIPLCSDQTS-------

NP_878301_danio_RhoGEF VLKKKLAFLSGGK------------DRRSGLILTIPLCTEQTS-------

NP_001106203_Homo_RhoGEF QLKKRFAYLSGGR------------GQDGSPVITFPDYPAFSE-------

NP_055893_Homo_RhoGEF QLHRQFAILSGGR------------GEDGAPIITFPEFSGFKH-------

NP_001093325_Homo_RhoGEF --MQDIAFLSGGR------------GKDNAWIITFPENCNFRC-------

NP_001033984_Drosophila_CRALTR LGTIVILPRPLN-------------DNGPRLALLRMACYDPSK-------

NP_651174_Drosophila_CRALTRIO QRGIHVIPMRPVS-------------PEGPRVIISQFRNIDPK-------

NP_496771_Caenorhabditis_CRALT QDDNKVLMFERTG----------KIDISGLVDNVLMHKFMQIK-------

XP_002110939_trichoplax_RhoGEF ILKEKIAFLSGGR------------DRSGRAIITFLARVTFVD-------

XP_640612_Dictyostelium_BCH IEALNFIYPAGK-------------DNLGRTIIVIIASHLPVR-------

XP_001420825_Ostreococcus_luc_ VSDAKAIYLEKSR------------DYIGRRVVVVIAAFLERL-------

NP_491993_Caenorhabditis_CRALT PECLEKYCGYGLLG-----------DTEGRPILMSLLGNVDVEG------

EFA82125_poly_NF1 LKQKKIFYKQGVT-------------KDKLPVFYYIARRYEAS-------

XP_638573_Dictyostelium_BCH VSRLNFTLQTTD--------------DQNRPIVVIIGSQLNSR-------

NP_871646_Caenorhabditis_CRALT LLDNQLMYLHGK-------------DLQNRHMLWIMMNKYKNG-------

XP_645940_Dictyostelium_BCH IADNNIFVQVG-------------CDMEGVPVFLLNASNLPPT-------

NP_171669_Arabidopsis_CRALTRIO DLSHNKMCMQG-------------HDKMGRPIAVAIGNRHNPS-------

XP_646383_dict_CRALTRIO GGDIREIGSAGCVYVN-------KRDKKGRPIIFAVPRNDTLKN------

XP_001347356_Plasmodium_fal_BC TENFDDLYKTDLLKVIG-------KDGYGSHIVLLIPCFIVAAG------

XP_002140404_Cryptosporidium_B NEVFNSYHGTRFLYY-------LCEDNLGRPVLVLVACFLPTD-------

NP_569990_Drosophila_CRALTRIO LIFFRMADLDPRTRN---------SVEETKIFVMMSDARFTKP-------

NP_199584_Arabidopsis_CRALTRIO LIGMSGYTKEGLP--------VFAIGVGLSTFDKASVHYYVQS-------

NP_508618_Caenorhabditis_CRALT MSAVAEYFPGGIMG-----------KSKRGDVVYMQAMAKAHP-------

XP_001702344_Chlamydomonas_BCH PSPFPGLLYTEGR------------DALGRAVVVLNTAMLPPK-------

XP_646478_dict_CRALTRIO IGTIFKTDKEGRP------------VMINHYHAINPDVIFKDG-------

NP_012390_Saccharomyces_CRALTR LQNVGILTFDANG------------DANKKAVTWNLYGQLVKK-------

XP_642079_dict_CRALTRIO AMKSSSFNIIGYR------------DNNGCSISYLHPSKAKPK-------

XP_645456_Dictyostelium_BCH MLERSRFLFQGPN------------DKNDRSVFYLIVNRVKP--------

XP_001548197_yeast_NF1 SEAVVSNRADG------------------LSMICIILRNIDTDG------

XP_571310_yeast_NF1 APPDATLLPASN----------------GRRIFYFIVSRVALID------

NP_593281_Schizosaccharomyces_ EHDLERLCFIGG------------YDNDNDKVIVVVTKNLELFK------

NP_001024391_Caenorhabditis_CR QNRLLWYIEYATIT----------VESIAHSIRSSEACKFQFL-------

NP_872173_Caenorhabditis_CRALT TSKNLHVFVQKMEG-----------TDIKEILKVMPLSYVLHS-------

NP_497717_Caenorhabditis_CRALT LQDRQWSDEHNAFLFVER-----AWSQPKEFIKTFKTSDYLLH-------

XP_001173154_Pan_BCH -----------------------SQPNYRYLMDNLFKYVIGT--------

XP_544705_Canis_BCH -----------------------GQPNYRYLMDNLFKYVIGT--------

XP_001374074_Monodelphis_BCH -----------------------GQPNYRYLMDNLFKYVIGT--------

NP_001008239_Mus_BCH -----------------------GQPNYRYLMDNLFKYVIGT--------

EDL84204_Rattus_BCH -----------------------GQPNYRYLMDNLFKYVIGT--------

XP_001100508_Macaca_BCH -----------------------SQPNYRYLMDNLFKYVIGT--------

NP_004321_Homo_BCH -----------------------SQPNYRYLMDNLFKYVIGT--------

XP_001510218_Ornithorhynchus_B -----------------------GQPNYRYLMDNLFKYVIGT--------

XP_413765_Gallus_BCH -----------------------SQPNYRYLMDNLFKYVIGT--------

XP_002195589_Taeniopygia_BCH -----------------------SQPNYRYLMDNLFKYVIGT--------

NP_001088131_Xenopus_BCH -----------------------SQPNYRYLMDNLFKYVIGT--------

CAF98314_Tetraodon_BCH -----------------------NQPNYRYIMDNLFKYVIGT--------

NP_957512_Danio_BCH -----------------------NQPNYRYIMDNLFKYVIGT--------

XP_001366186_Monodelphis_BCH -----------------------SRADYHYVMENLFLYVIST--------

XP_001916938_Equus_BCH -----------------------SRADYHYVMENLFLYVIST--------

NP_056040_Homo_BCH -----------------------SRADYHYVMENLFLYVIST--------

XP_001146885_Pan_BCH -----------------------SRADYHYVMENLFLYVIST--------

NP_001126916_Pongo_abe_BCH -----------------------SRADYHYVMENLFLYVIST--------

NP_001098852_Bos_BCH -----------------------SRADYHYVMENLFLYVIST--------

NP_851993_Mus_BCH -----------------------SRADYHYVMENLFLYVIST--------

Q5BJR4_Rattus_BCH -----------------------SRADYHYVMENLFLYVIST--------

XP_002187337_Taeniopygia_BCH -----------------------SRTDYNYVMENLFLYVIST--------

XP_001231886_Gallus_BCH -----------------------SRTDYNYVMENLFLYVIST--------

XP_850604_Canis_BCH -----------------------SRADYHYVMENLFLYVIST--------

XP_001515884_Ornithorhynchus_B -----------------------SRADYNYVMENLFLYVIST--------

CAG08427_Tetraodon_BCH -----------------------NCDNYSYVMENLFLYVINT--------

NP_001104624_Danio_BCH -----------------------DREDYHEIMENLFLYVIST--------

CAF97194_Tetraodon_BCH -----------------------DREDYHEIMENLFLYVIST--------

NP_001133314_Salmo_BCH -----------------------DSEDYHEVMEHLFLYVIST--------

XP_001374042_Monodelphis_BCH -----------------------NSPDYHYIMENLFLYVISS--------

XP_001511480_Ornithorhynchus_B -----------------------NSADYHYIMENLFLYVISS--------

XP_542168_Canis_BCH -----------------------SSPDYHYIMENLFLYVISS--------

NP_001125042_Pongo_abe_BCH -----------------------SLPDYHYIMENLFLYVISS--------

NP_001075898_Bos_BCH -----------------------SSPDYHYIMENLFLYVISS--------

NP_848777_Mus_BCH -----------------------SSPDYHYIMENLFLYVISS--------

NP_001035280_Rattus_BCH -----------------------SSPDYHYIMENLFLYVISS--------

XP_524457_Pan_BCH -----------------------SLADYHYIMENLFLYVISS--------

BAC03859_Homo_BCH -----------------------SLPDYHYIMENLFLYVISS--------

XP_001493463_Equus_BCH -----------------------SSPDYHYIMENLFLYVISS--------

XP_418214_Gallus_BCH -----------------------NLADYHYIMENLFLYVISS--------

XP_002195707_Taeniopygia_BCH -----------------------NLADYHYIMENLFLYVISS--------

CAF91924_Tetraodon_BCH -----------------------SCGDYAYIMENLFLYVVSS--------

XP_001343600_Danio_BCH -----------------------GCADYNYIMENLFLYVISS--------

CAG08009_Tetraodon_BCH -----------------------SCPDYHYIMENLFLYMVSS--------

CAN87813_Danio_BCH -----------------------SCPDYHYLMENLFLYVVSS--------

XP_001170701_Pan_BCH -----------------------SIPNYTYVMEHLFRYMVGT--------

NP_612122_Homo_BCH -----------------------SIPNYTYVMEHLFRYMVGT--------

XP_001106978_Macaca_BCH -----------------------SIPNYTYVMEHLFRYMVGT--------

XP_001491310_Equus_BCH -----------------------SIPNYTYVMEHLFRYMVGT--------

XP_001928046_Sus_BCH -----------------------SIPNYTYVMEHLFRYMVGT--------

NP_001073089_Bos_BCH -----------------------SIPNYTYVMEHLFRYIVGT--------

EDL38792_Mus_BCH -----------------------SIPNYTYIMEHLFRYMVGT--------

NP_001121659_Rattus_BCH -----------------------SIPNYTYVMEHLFRYMVGT--------

XP_540308_Canis_BCH -----------------------SIPNYTYVMEHLFRYMVGT--------

XP_001371249_Monodelphis_BCH -----------------------NVPDYSYIMEHLFRYMVGT--------

NP_001086594_Xenopus_BCH -----------------------SIPDYQYVLNNLFRYIIGT--------

NP_001121866_Danio_BCH -----------------------TTEHYEYVMDNLFRYIIGT--------

XP_002127893_Ciona_BCH -----------------------QQTDYRYIMDNLFLYIVST--------

EEC09901_Ixodes_BCH -----------------------GRRDYDYVMDNLFLYVLST--------

XP_001947520_Acyrthosiphon_BCH -----------------------DRVDYNYVMNNLFAYVVST--------

EEB18677_Pediculus_BCH -----------------------SRADYDYVMNNLFMYVIST--------

XP_315572_Anopheles_BCH -----------------------SRADYHYVMNNLFLYVVKT--------

XP_001648558_Aedes_BCH -----------------------SRADYHYVMNNLFLYVVKT--------

ABY20545_Drosophila_mel_BCH -----------------------SRADYSYVMDNLFLYVVKT--------

XP_974968_Tribolium_BCH -----------------------SRVDYVYVMDNLFLYILWT--------

XP_783795_Strongylocentrotus_B -----------------------SRKDYTYLMNNLFLYVVST--------

XP_001626140_Nematostella_BCH -----------------------AEKNYDFLMEQVFFYLIST--------

XP_002126187_Ciona_BCH -----------------------TVANYAWVIDNLFLYFLST--------

XP_508398_Pan_BCH -------------------------PSHQLDHSKLLGYLKHT--------

CAM14521_Mus_BCH -------------------------PSHQLDHSKLLGYLKHT--------

NP_001124902_Pongo_abe_BCH -------------------------PSHQLDHSKLLGYLKHT--------

XP_001101907_Macaca_BCH -------------------------PSHQLDHSKLLGYLKHT--------

BAG60756_Homo_BCH -------------------------PSHQLDHSKLLGYLKHT--------

NP_001101217_Rattus_BCH -------------------------PSHQLDHSKLLGYLKHT--------

XP_001490021_Equus_BCH -------------------------PSHQLDHSKLLGYLKHT--------

XP_871999_Bos_BCH -------------------------PSHQLDHSKLLGYLKHT--------

XP_001369707_Monodelphis_BCH -------------------------PSHQLDHIKLLEYLKYT--------

XP_426422_Gallus_BCH -------------------------PSHQLDHVKLLGYLKFT--------

XP_002198819_Taeniopygia_BCH -------------------------PSHQLDHVKLLGYLKFT--------

NP_001086509_Xenopus_BCH -------------------------ACHEIDHVKLLQYLKHT--------

NP_001017781_Danio_BCH -------------------------PQHQLDHHKLLMYLKQT--------

EDL04450_Mus_BCH -------------------------PLHQLNHQRLLEYLKYT--------

NP_001004242_Rattus_BCH -------------------------PLHQLNHQRLLEYLKYT--------

CAQ06715_Homo_BCH -------------------------PSHELDHQRLLEYLKYT--------

XP_416463_Gallus_BCH -------------------------PSYQLNHTRLLEYLKYT--------

NP_001085153_Xenopus_BCH -------------------------PCHEIDHVRLLEYMKHT--------

EEB11750_Pediculus_BCH -------------------------SNKELNHQRLLKYLMFT--------

XP_001950301_Acyrthosiphon_BCH -------------------------SNKEVDHPRLLRYLMYT--------

XP_001604462_Nasonia_BCH -------------------------GKEAFNHAKLLRYLTHT--------

XP_624226_Apis_BCH -------------------------GKETFNYAKLLRYLTHT--------

XP_002121397_Ciona_BCH -------------------------SNKELDHKKLLRYLKFS--------

XP_970580_Tribolium_BCH -------------------------PIAEINHSLFLNYLTHT--------

XP_002160831_Hydra_BCH -------------------------NRKDIDHQQLLCFLKEV--------

NP_001022390_Caenorhabditis_el -------------------------SSKEIDHARLLQYLVQI--------

XP_001893352_Brugia_BCH -------------------------SNKTFDHAKFLRFLQFT--------

XP_002117739_Trichoplax_BCH -------------------------PVAEIEHERLLEYLKKT--------

XP_002572184_Schistosoma_BCH -------------------------AADLIDHQHLLMYITKT--------

EEC10256_Ixodes_BCH -------------------------SNKELDHAKFLRYLMHT--------

XP_001746114_Monosiga_BCH -------------------------PRSELSHDDLLQYMQHT--------

XP_001835505_Coprinopsis_BCH -------------------------DPQAVSYDLLLSRILAY--------

XP_001876797_Laccaria_BCH -------------------------DPQAVSYDLLLSRILSY--------

XP_760803_Ustilago_BCH -------------------------DPSEVDYDALLDRIMDT--------

XP_001843366_Culex_BCH -------------------------ERKDLNTNIFIDFIIKS--------

XP_001659702_Aedes_BCH -------------------------ERKDLNTNIFIDFIIKS--------

NP_648552_Drosophila_mel_BCH -------------------------EKSQLEG--FVREIIKE--------

XP_001182784_Strongylocentrotu -----------------------SIDL-----NKVLLYVIHL--------

XP_001622394_Nematostella_BCH -----------------------NTDL-----GKAVAYFISV--------

XP_396878_Apis_BCH -----------------------KINL-----DKALLYLIQL--------

XP_001815509_Tribolium_BCH -----------------------KINL-----DKALLYLITL--------

NP_724599_Drosophila_mel_BCH -----------------------NIDL-----EKALLYLIKL--------

NP_724597_Drosophila_mel_BCH -----------------------NIDL-----EKALLYLIKL--------

XP_001867337_Culex_BCH -----------------------NIDL-----EKALLYLIYL--------

XP_001688494_Anopheles_BCH -----------------------NIDL-----EKALLYLIYL--------

XP_001660566_Aedes_BCH -----------------------NIDL-----EKALLYLIYL--------

EEB16863_Pediculus_BCH -----------------------EINL-----DKALLYLIYL--------

ACO15636_Caligus_BCH -----------------------EVDL-----ERALLFLLKV--------

XP_002112464_Trichoplax_BCH -----------------------LTDL-----NKAISYYAYL--------

XP_423602_Gallus_BCH -----------------------LIDM-----EKALLYFIHV--------

XP_002197809_Taeniopygia_BCH -----------------------LIDM-----EKALLYFIHV--------

XP_001144527_Pan_BCH -----------------------LIDM-----DKALLYFIHV--------

NP_060156_Homo_BCH -----------------------LIDM-----DKALLYFIHV--------

XP_001500874_Equus_BCH -----------------------LIDM-----DKALLYFIHV--------

NP_001039478_Bos_BCH -----------------------LIDM-----DKALLYFIHV--------

XP_533021_Canis_BCH -----------------------LIDM-----DKALLYFIHV--------

XP_001365992_Monodelphis_BCH -----------------------LIDM-----DKALLYFIHV--------

NP_001013219_Rattus_BCH -----------------------LIDM-----DKALLYFIHV--------

NP_034399_Mus_BCH -----------------------LIDM-----DKALLYFIHV--------

XP_001104476_Macaca_BCH -----------------------LIDM-----DKALLYFIHV--------

NP_001004563_Danio_BCH -----------------------LIDM-----EKALLYFIHV--------

CAF93717_Tetraodon_BCH -----------------------LLDP-----EKALLYFIHV--------

ACO13754_Esox_BCH -----------------------LIDM-----EKALLYFIHV--------

NP_001088345_Xenopus_BCH -----------------------LIDM-----EKALLYFIHM--------

XP_001512063_Ornithorhynchus_B -----------------------LIDM-----DKALLYFIHV--------

XP_001776374_Physcomitrella_BC -----------------------CLDL-----ERFVLYVVK---------

XP_001759372_physcomitrella_BC -----------------------CLDL-----ERFVLYVVK---------

EEF01185_Populus_BCH -----------------------CLDL-----ERFVLHVIK---------

EEE89642_Populus_BCH -----------------------CLDL-----ERFVLHVVK---------

EEF38953_Ricinus_BCH -----------------------CLDL-----ERFVLYVVK---------

NP_564960_Arabidopsis_BCH -----------------------CLDL-----ERFVLYVIK---------

XP_002467159_Sorghum_BCH -----------------------CLDL-----ERFVLHVVK---------

ACG47767_Zea_BCH -----------------------CLDL-----ERFVLHVVK---------

NP_001064706_Oryza_BCH -----------------------CLDL-----ERFVLHVVK---------

XP_002465844_Sorghum_BCH -----------------------CLDL-----ERFVLYVIK---------

ACG45534_Zea_BCH -----------------------CLDL-----ERFVLYVVK---------

ABK24296_Picea_BCH -----------------------CLDL-----ERFVLYVVK---------

EEE58332_Oryza_BCH -----------------------CLDL-----ERFILYVVKLILTGLNLP

EEE91516_Populus_BCH -----------------------VVSG-----ERLKKYIFHK--------

EEF30886_Ricinus_BCH -----------------------VISA-----ERLKKYIFHK--------

ACU17631_Glycine_BCH -----------------------VVSA-----ERLKRYVFHK--------

ABD28324_Medicago_BCH -----------------------VVSA-----ERLKRYVFHK--------

ABR17649_Picea_BCH -----------------------VIGG-----ERLKKYICQK--------

XP_001754720_Physcomitrella_BC R-------------GVLWFDAAPAIDV-----EKLKVFVLHK--------

NP_001047687_Oryza_BCH ---------------------ARALGG--RAEAALRGYVRRR--------

ACG26273_Zea_BCH ---------------------ARALGG--RAEEALKAHLRRR--------

ACG34784_Zea_BCH ---------------------ARALGG--RAEEALRSYLRER--------

BAF15465_Oryza_BCH ---------------------ARELGGGGHAEAALKGYVRRR--------

EEF31637_Ricinus_BCH ---------------------ARIITV-----DALKSYLEEK--------

NP_195300_Arabidopsis_BCH ---------------------ARFLSL-----DVLKKYLEEK--------

ACJ85167_Medicago_BCH ---------------------ARLVSV-----EVLKKFLEER--------

ACU14441_Glycine_BCH ---------------------ARLVSV-----EVLKKYLEER--------

EEE75109_Populus_BCH ---------------------ARYLSV-----DALKNYLEEN--------

EEE93960_Populus_BCH GIFWDLFFSFCCKSNLFCGVLAREVSG-----EVLKKYLEEK--------

EEE81211_Populus_BCH ---------------------ARAVSG-----EVLKKYLEEK--------

EEF52804_Ricinus_BCH ---------------------ARLVSS-----EALHKYLEDK--------

CAN69115_Vitis_BCH ---------------------ARVISV-----EVLKKYLEEK--------

XP_001699293_Chlamydomonas_BCH ---------------------ARVLKT-----DRVFRYFAHT--------

XP_002168194_Cnidarian_NF1 -----------------------------ISDEVFLYHILLT--------

XP_001744997_Coano_NF1 -----------------------------LDAEHIMYHALLT--------

AAB59558_human_NF1 -----------------------------INGDLLIYHVLLT--------

XP_692937.3_Danio_NF1 -----------------------------INGDLLIYHVLLT--------

XP_002587546_lancelet_NF1 -----------------------------MNGDLLLYHILLS--------

AAB58976_Drosophila_NF1 -----------------------------TNGDLLIYHVILT--------

XP_001196417_Echinoderm_NF1 -----------------------------IDFEQLMYHILLT--------

XP_002576216_tremtoda_NF1 ---------------------------QRVEYPFIICLVSMT--------

XP_002115170_placozoa_NF1 -----------------------------MNEELVIYYIMSS--------

XP_002121778_ciona_NF1 -----------------------------IREDILMFYILQA--------

NP_013796_Saccharomyces_CRALTR ------------------------VTSEERMLKNLVWEYESVVQYRLPAC

NP_012832_Saccharomyces_CRALTR ------------------------ITTEKQMLRNLVKEYELFATYRVPAC

NP_195629_Arabidopsis_CRALTRIO ------------------------VTTIDRYVKYHVKEFEKTFNVKFPAC

NP_036561_Homo_CRALTRIO ------------------------SASKQDLLRTKMRECELLL-QECAHQ

NP_777635_Homo_CRALTRIO ------------------------SVTKQDLLKTKMRDCERIL-HECDLQ

NP_001091144_Xenopus_CRALTRIO ------------------------SASKQDLMKTKMRDCELMH-RACLMQ

NP_001093463_Danio_CRALTRIO ------------------------SASKQDLIKSKVRDCEILQ-KDCDRQ

NP_777637_Homo_CRALTRIO ------------------------SASKQDMIRKRIKVCELLL-HECELQ

NP_612042.3_Drosophila_CRALTRI ------------------------CVTRFEFQKYLVLLLERFM-KIAYDQ

NP_001087870_Xenopus_CRALTRIO ------------------------ALGEESLLRHVLSINEEGL-RRCEEN

NP_001085706_Xenopus_CRALTRIO ------------------------ALGEESLLRHVLSINEEGL-RRCEEN

NP_001137470_Homo_CRALTRIO ------------------------ALGEEALLRYVLSINEEGL-RRCEEN

NP_957392_Danio_CRALTRIO ------------------------ALGEETLLRHVLSINEEGL-RRCEEN

NP_055507_Homo_CRALTRIO ------------------------AVGEEALLRHVLSVNEEGQ-KRCEGS

NP_001040875_Caenorhabditis_CR ------------------------SCGVENLVKLTLSICEDGL-QRAAEA

NP_609028_Drosophila_CRALTRIO ------------------------SLGMDGLLRLALHICEEGI-QKINES

NP_506408_Caenorhabditis_CRALT ------------------------SYPTNEILRARVHDLESML-KAVMDL

NP_510554_Caenorhabditis_CRALT ------------------------TYSILEVMRARMVDLEQML-HHVMEL

NP_495168_Caenorhabditis_CRALT ------------------------SVHLSDFLIQRFKFQEKML-AAMNEM

NP_505746_Caenorhabditis_CRALT ------------------------SVQPTEYLHEMFRSFEEIQ-RRLMKM

CAB78040_Arabidopsis_CRALTRIO ------------------------TFSDEEKRERFLRWRIQFLEKSIRNL

NP_177360_Arabidopsis_CRALTRIO -------------------------FSDKEKLNKFLSWRIQLQEKCVRAI

NP_013484_Saccharomyces_CRALTR ------------------------DQTEQELEKFSLLVIEQSK-LFFKE-

XP_643391_Dict_CRALTRIO ------------------------NRDLNESMRYCVYVMENGK-SMLKD-

NP_728794_Drosophila_CRALTRIO ------------------------ERDIDELTRFIVYNLEEAC-KKCFE-

NP_014168_Saccharomyces_CRALTR -------------------------KTSHRQVQHLVFMLERVIDFMP---

NP_014135_Saccharomyces_CRALTR -------------------------ESSFRQVQELVYMMETATTVAP---

XP_629043_dict_CRALTRIO -------------------------KNHDNQIRLMVYNIERAISLMDKT-

NP_195382_Arabidopsis_CRALTRIO -------------------------TSQEGNIRHLVYLLENAIINLP---

BAH19871_Arabidopsis_CRALTRIO -------------------------KSYKGQIRILVYCMENAILNLP---

NP_001088147_Xenopus_CRALTRIO ------------------------GKTNDDKKKFVAFWLERYARREP---

NP_001088926_Xenopus_CRALTRIO ------------------------GKTNEDKKKFVAFWLERYARREP---

NP_689794_Homo_CRALTRIO ------------------------QKTILDKKKLIAFWLERYAKREN---

NP_001007294_Danio_CRALTRIO ------------------------PKTVLDKKRYVAFWLERYAKREP---

NP_729113_Drosophila_CRALTRIO -------------------------RNQEDLLRILVFWIERLQRDSN---

NP_000317_Homo_CRALTRIO ------------------------EITFDEILQAYCFILEKLLE-NEETQ

NP_001080386_Xenopus_CRALTRIO ------------------------EITFDEILRAYCIILENLLE-NEETQ

NP_956999_Danio_CRALTRIO ------------------------EVTFDETLRAYCVILEKLLE-NEETQ

NP_775790_Homo_CRALTRIO ------------------------RNSFTDILRAILLSLEVLIE-DPELQ

NP_001088883_Xenopus_CRALTRIO ------------------------RNSFVDILRAILLSLEVLIE-DQELQ

NP_001010852_Homo_CRALTRIO ------------------------RYTLVDILRAILLSLEAMIE-DPELQ

NP_001020716_Danio_CRALTRIO ------------------------RYTFVDILRAILLSLEAMIE-DPELQ

NP_001034288_Homo_CRALTRIO ------------------------NYPITENIRAIYLTLEKLIQ-SEETQ

NP_997755_Danio_CRALTRIO ------------------------DYPFVDNIRAIYLTLEKLIQ-PEETQ

NP_000361_Homo_CRALTRIO ------------------------VFTAYDVFRVSLITSELIVQ-EVETQ

NP_724195_Drosophila_CRALTRIO ------------------------QVTVDDIFRATIVLQELGSL-EPISQ

NP_609120_Drosophila_CRALTRIO ------------------------QVPLVDLFRGIQLTVLGSMV-EPYSQ

NP_001079459_Xenopus_CRALTRIO ------------------------VTTHKAVLQAIIYQLDKAIE-RVETQ

NP_001081203_Xenopus_CRALTRIO ------------------------VTTHKAVLQAIIYQLDKAIE-RVETQ

NP_572576_Drosophila_CRALTRIO ------------------------SVSHTTTLQGIVYQLDSALQ-DSETQ

NP_002824_Homo_CRALTRIO ------------------------KSVQHVVLQALFYLLDRAVD-SFETQ

NP_724813_Drosophila_CRALTRIO ------------------------EFKLLDLFRYQTMITEQSIREDDHSN

NP_610507_Drosophila_CRALTRIO ------------------------KHSVSDIFRFHAFRAEIEINTDDNWN

NP_001021496_caenorhabditis_Rh --------------------------VNQDNLRNVFLYLFEVT-------

XP_001891671_brugia_RhoGEF --------------------------INPDHIRNILIYLHAVT-------

NP_009049_Homo_RhoGEF --------------------------IRQEDLRRLISYLACIP-------

NP_001097996_danio_RhoGEF --------------------------IRHEDLRRLIAYLAGIP-------

NP_001019831_Homo_RhoGEF --------------------------IRQEDLRKLVTYLASVP-------

XP_002598613_branchiostoma_Rho --------------------------IRYDDLKRLMTYLASIP-------

NP_651960_drosophila_RhoGEF --------------------------LKPEDLRRLLSYLISIP-------

XP_002578747_schistosoma_RhoGE --------------------------IPIEDLYVLVRYLTYLP-------

XP_001635815_nematostella_RhoG --------------------------QDEESIGQIVSYLANLP-------

NP_835224.3_Homo_RhoGEF ----------------------------MDELSVTLDYLLSIP-------

NP_001085886_xenopus_RhoGEF ----------------------------MDELSVTLDYLLSIP-------

NP_878301_danio_RhoGEF ----------------------------MEELSTTLDYLLGIP-------

NP_001106203_Homo_RhoGEF --------------------------IPDKEFQNVMTYLTSIP-------

NP_055893_Homo_RhoGEF --------------------------IPDEDFLNVMTYLTSIP-------

NP_001093325_Homo_RhoGEF --------------------------IPEEVIAKVLTYLTSIA-------

NP_001033984_Drosophila_CRALTR -----------------------YTLQEVNRAAGLMQQIMLDE-------

NP_651174_Drosophila_CRALTRIO ------------------------KSNPREAFKLIFIMLELLA-------

NP_496771_Caenorhabditis_CRALT ------------------------LKMMEGVHQKVVAAERKTG-------

XP_002110939_trichoplax_RhoGEF --------------------------IESERLIKLINYLASIP-------

XP_640612_Dictyostelium_BCH ----------------------------EMDMERVLLYTISI--------

XP_001420825_Ostreococcus_luc_ --------------------------IKSGEEQRLLAHVAKELR------

NP_491993_Caenorhabditis_CRALT ------------------------LLRSVASLDYIKFSLAAIEKGMKLCE

EFA82125_poly_NF1 -----------------------------FDMEQLLYLILKT--------

XP_638573_Dictyostelium_BCH ----------------------------KDLYDQVLLYLIRV--------

NP_871646_Caenorhabditis_CRALT ----------------------------DDGFEKLFTFWIER--------

XP_645940_Dictyostelium_BCH -----------------------------SQIEPVLIGILKT--------

NP_171669_Arabidopsis_CRALTRIO -------------------------KGNPDEFKRFVVYTLEK--------

XP_646383_dict_CRALTRIO -------------------------VPSELKFKNLVYWLEQGFS------

XP_001347356_Plasmodium_fal_BC -----------------------------ADPEKTLRYAILT--------

XP_002140404_Cryptosporidium_B ----------------------------VSALDKAMRYAVSS--------

NP_569990_Drosophila_CRALTRIO --------------------------DVERETGSGADYVLDE--------

NP_199584_Arabidopsis_CRALTRIO -----------------------HIQINEYRDRVLLPSISKKN-------

NP_508618_Caenorhabditis_CRALT -----------------------KTLVKAGPTSQLFQLCISETEMSFKII

XP_001702344_Chlamydomonas_BCH --------------------------SAKVKKEDILQYVLQQ--------

XP_646478_dict_CRALTRIO -----------------------VDQFVRWKVQQMEIAIRDT--------

NP_012390_Saccharomyces_CRALTR -------------------------KELFQNVDKFVRYRIGLMEKG----

XP_642079_dict_CRALTRIO -------------------------DFTLKEYMTFLLWSQDQS-------

XP_645456_Dictyostelium_BCH --------------------------EVFDNVNPLIAHIFKV--------

XP_001548197_yeast_NF1 ------------------------------TDYDLLLYVYLK--------

XP_571310_yeast_NF1 -------------------------------YDLLAYHVFLT--------

NP_593281_Schizosaccharomyces_ ------------------------KYDDINLIKEAYNHVHKLIQ------

NP_001024391_Caenorhabditis_CR --------------------------QFEYMLRKVMEQEERTG-------

NP_872173_Caenorhabditis_CRALT ------------------------YFMLQENFSRAMAHTERKTG------

NP_497717_Caenorhabditis_CRALT ------------------------CFGYSEMLQQLILRREKKQ-------

XP_001173154_Pan_BCH ---LELLVAENYMIVYLNGATT----------------------------

XP_544705_Canis_BCH ---LELLVA-ENYMIVYLNGATT---------------------------

XP_001374074_Monodelphis_BCH ---LELLVA-ENYMIVYLNGATT---------------------------

NP_001008239_Mus_BCH ---LELLVA-ENYMIIYLNGATT---------------------------

EDL84204_Rattus_BCH ---LELLVA-ENYMIIYLNGATT---------------------------

XP_001100508_Macaca_BCH ---LELLVA-ENYMIVYLNGATT---------------------------

NP_004321_Homo_BCH ---LELLVA-ENYMIVYLNGATT---------------------------

XP_001510218_Ornithorhynchus_B ---LELLVA-ENYMIVYLNGATS---------------------------

XP_413765_Gallus_BCH ---LELLVA-ENYMIVYLNGATT---------------------------

XP_002195589_Taeniopygia_BCH ---LELLVA-ENYMIVYLNGATT---------------------------

NP_001088131_Xenopus_BCH ---LEMLVA-ENYMIVYLNGATT---------------------------

CAF98314_Tetraodon_BCH ---LELLVA-ENYMIVYLNGATS---------------------------

NP_957512_Danio_BCH ---LELLVA-ENYMIVYLNGATS---------------------------

XP_001366186_Monodelphis_BCH ---LELMVA-EDYMIVYLNGATP---------------------------

XP_001916938_Equus_BCH ---LELMVA-EDYMIVYLNGATP---------------------------

NP_056040_Homo_BCH ---LELMVA-EDYMIVYLNGATP---------------------------

XP_001146885_Pan_BCH ---LELMVA-EDYMIVYLNGATP---------------------------

NP_001126916_Pongo_abe_BCH ---LELMVA-EDYMIVYLNGATP---------------------------

NP_001098852_Bos_BCH ---LELMVA-EDYMIVYLNGATP---------------------------

NP_851993_Mus_BCH ---LELMVA-EDYMIVYLNGATP---------------------------

Q5BJR4_Rattus_BCH ---LELMVA-EDYMIVYLNGATP---------------------------

XP_002187337_Taeniopygia_BCH ---LELMVA-EDYMIVYLNGATP---------------------------

XP_001231886_Gallus_BCH ---LELMVA-EDYMIVYLNGATP---------------------------

XP_850604_Canis_BCH ---LELMVA-EDYMIVYLNGATP---------------------------

XP_001515884_Ornithorhynchus_B ---LELMVA-EDYMIVYLNGATP---------------------------

CAG08427_Tetraodon_BCH ---LELMVA-EDYMIVYLNGATP---------------------------

NP_001104624_Danio_BCH ---LELMVA-EDYMIVYLNGATP---------------------------

CAF97194_Tetraodon_BCH ---LELMVA-EDYMIVYLNGATP---------------------------

NP_001133314_Salmo_BCH ---LELMVA-EDYMIIYLNGATP---------------------------

XP_001374042_Monodelphis_BCH ---LELLVA-EDYMIVYLNGATP---------------------------

XP_001511480_Ornithorhynchus_B ---LELLVA-EDYMIVYLNGATP---------------------------

XP_542168_Canis_BCH ---LELLVA-EDYMIVYLNGATP---------------------------

NP_001125042_Pongo_abe_BCH ---LELLVA-EDYMIVYLNGATP---------------------------

NP_001075898_Bos_BCH ---LELLVA-EDYMIVYLNGATP---------------------------

NP_848777_Mus_BCH ---LELLVA-EDYMIVYLNGATP---------------------------

NP_001035280_Rattus_BCH ---LELLVA-EDYMIVYLNGATP---------------------------

XP_524457_Pan_BCH ---LELLVA-EDYMIVYLNGATP---------------------------

BAC03859_Homo_BCH ---LELLVA-EDYMIVYLNGATP---------------------------

XP_001493463_Equus_BCH ---LELLVA-EDYMIVYLNGATP---------------------------

XP_418214_Gallus_BCH ---LELLVA-EDYMIVYLNGATP---------------------------

XP_002195707_Taeniopygia_BCH ---LELLVA-EDYMIVYLNGATP---------------------------

CAF91924_Tetraodon_BCH ---LELLVA-EDYMIVYLNGATP---------------------------

XP_001343600_Danio_BCH ---LEALVA-EDYMIIYLNGATP---------------------------

CAG08009_Tetraodon_BCH ---LEMLVA-EDYLIVYMNGATP---------------------------

CAN87813_Danio_BCH ---LEMLVA-EDYLIIYMNGGTP---------------------------

XP_001170701_Pan_BCH ---LELLVA-ENYLLVHLSGGTS---------------------------

NP_612122_Homo_BCH ---LELLVA-ENYLLVHLSGGTS---------------------------

XP_001106978_Macaca_BCH ---LELLVA-ENYLLVHLSGGTS---------------------------

XP_001491310_Equus_BCH ---LELLVA-ENYLLVHLSGGTS---------------------------

XP_001928046_Sus_BCH ---LELLVA-ENYLLVHLSGGTN---------------------------

NP_001073089_Bos_BCH ---LELLVA-ENYLLVHLSGGTS---------------------------

EDL38792_Mus_BCH ---LELLVA-ENYLLVHLSGGTS---------------------------

NP_001121659_Rattus_BCH ---LELLVA-ENYLLVHLSGGTS---------------------------

XP_540308_Canis_BCH ---LELLVA-ENYLLVHLSGGTS---------------------------

XP_001371249_Monodelphis_BCH ---LELLVA-ENYLLVHLSGGTN---------------------------

NP_001086594_Xenopus_BCH ---LDLMVA-DDYMLVYLNGATP---------------------------

NP_001121866_Danio_BCH ---LDLMVS-ENYILVYLCGMAP---------------------------

XP_002127893_Ciona_BCH ---LEMLVA-EDYMIIFFNGGCR---------------------------

EEC09901_Ixodes_BCH ---LDQLVA-EDYVLIYLHGATE---------------------------

XP_001947520_Acyrthosiphon_BCH ---LDQLVT-EDYVLVYLQGGTS---------------------------

EEB18677_Pediculus_BCH ---LDQLVT-DDYMVIYLHGATA---------------------------

XP_315572_Anopheles_BCH ---LEQLVT-EDYVLVYLHGGSS---------------------------

XP_001648558_Aedes_BCH ---LEQLVT-EDYVLVYLHGGSS---------------------------

ABY20545_Drosophila_mel_BCH ---LEQLVT-DDYVLIYLHGGSN---------------------------

XP_974968_Tribolium_BCH ---LERLVT-DDYVLVYLHGGAT---------------------------

XP_783795_Strongylocentrotus_B ---LELLVA-QEYIIIYFHGSAS---------------------------

XP_001626140_Nematostella_BCH ---LELLATTEEFYIVYFNGGTT---------------------------

XP_002126187_Ciona_BCH ---VEMLVQGNEYVVVFFNNSVA---------------------------

XP_508398_Pan_BCH ---LDQYVE-SDYTLLYLHHGLT---------------------------

CAM14521_Mus_BCH ---LDQYVE-SDYTLLYLHHGLT---------------------------

NP_001124902_Pongo_abe_BCH ---LDQYVE-SDYTLLYLHHGLT---------------------------

XP_001101907_Macaca_BCH ---LDQYVE-SDYTLLYLHHGLT---------------------------

BAG60756_Homo_BCH ---LDQYVE-SDYTLLYLHHGLT---------------------------

NP_001101217_Rattus_BCH ---LDQYVE-SDYTLLYLHHGLT---------------------------

XP_001490021_Equus_BCH ---LDQYVE-SDYTLLYLHHGLT---------------------------

XP_871999_Bos_BCH ---LDQYVE-SDYTLLYLHHGLT---------------------------

XP_001369707_Monodelphis_BCH ---LDQYVE-SDYTLLYLHHGLT---------------------------

XP_426422_Gallus_BCH ---LDQYVE-SDYTLVYLHHGLT---------------------------

XP_002198819_Taeniopygia_BCH ---LDQYVE-SDYTLVYLHHGLT---------------------------

NP_001086509_Xenopus_BCH ---LDQYVE-SDYTLVYLHHGLT---------------------------

NP_001017781_Danio_BCH ---LDKYVE-SDYTLIYFHHGLT---------------------------

EDL04450_Mus_BCH ---LDQHVE-NDYTIVYFHYGLS---------------------------

NP_001004242_Rattus_BCH ---LDQHVE-NDYTIVYFHYGLS---------------------------

CAQ06715_Homo_BCH ---LDQYVE-NDYTIVYFHYGLN---------------------------

XP_416463_Gallus_BCH ---LDQYVE-NDYTVVYFHYGLK---------------------------

NP_001085153_Xenopus_BCH ---LDQYVE-NDYTLVYFHYGLN---------------------------

EEB11750_Pediculus_BCH ---LDKYVE-QDYSLVYLHYGLN---------------------------

XP_001950301_Acyrthosiphon_BCH ---LDKFVE-QDYSLVYFHYGLT---------------------------

XP_001604462_Nasonia_BCH ---LDTFVE-QDYSLVYFHHGLT---------------------------

XP_624226_Apis_BCH ---LDTFVE-QDYSLVYFHYGLT---------------------------

XP_002121397_Ciona_BCH ---LDKYVE-SDYSVVYLHYGLN---------------------------

XP_970580_Tribolium_BCH ---LDQYVE-QDYSLVYFHYGLS---------------------------

XP_002160831_Hydra_BCH ---LDCYVE-NDYTLVYFHYGLR---------------------------

NP_001022390_Caenorhabditis_el ---IDKIVD-QDYTIVYFHYGLR---------------------------

XP_001893352_Brugia_BCH ---LDKLVE-LDYTIVYFHYGLR---------------------------

XP_002117739_Trichoplax_BCH ---LDSYVE-SDYSLIYFHYGLN---------------------------

XP_002572184_Schistosoma_BCH ---LEQYVS-IDYVLIYFHFGLT---------------------------

EEC10256_Ixodes_BCH ---LDQFVE-NDYTLVYFHHGLN---------------------------

XP_001746114_Monosiga_BCH ---MDTIVD-ADYCIVYFHHGLSSQVAYPLTRTCPCSPIDLHSHTRTCSL

XP_001835505_Coprinopsis_BCH ---LNLYVE-ADYTVVFFAAGNR---------------------------

XP_001876797_Laccaria_BCH ---LNLYVE-ADYTVIFFAAGGR---------------------------

XP_760803_Ustilago_BCH ---LDLFVE-NDYTVVYFAAGGH---------------------------

XP_001843366_Culex_BCH ---MEEFVQ-NDYIIAYFHQGMK---------------------------

XP_001659702_Aedes_BCH ---MEEFVQ-NDYIIAYFHQGMK---------------------------

NP_648552_Drosophila_mel_BCH ---IEPFVE-NDYILVYFHQGLK---------------------------

XP_001182784_Strongylocentrotu ----LDSIVNQDYVVVYFHTMSS---------------------------

XP_001622394_Nematostella_BCH ----LDRIVNRDYVVVYFHTHST---------------------------

XP_396878_Apis_BCH ----LDPIVKGDYVIAYFHTLTT---------------------------

XP_001815509_Tribolium_BCH ----LDPIVKGDYVIAYFHTLTS---------------------------

NP_724599_Drosophila_mel_BCH ----LDPIVKGDYVISYFHTLTS---------------------------

NP_724597_Drosophila_mel_BCH ----LDPIVKGDYVISYFHTLTS---------------------------

XP_001867337_Culex_BCH ----LDPIVKGDYVIAYFHTLTG---------------------------

XP_001688494_Anopheles_BCH ----LDPIVKGDYVIAYFHTLTS---------------------------

XP_001660566_Aedes_BCH ----LDPIVKGDYVIAYFHTLTS---------------------------

EEB16863_Pediculus_BCH ----LDPLVKSDYVIIYFHTLTS---------------------------

ACO15636_Caligus_BCH ----VDPISSSGYVVVYFHSKTS---------------------------

XP_002112464_Trichoplax_BCH ----MDDMVDNDYIAIYFHTLTN---------------------------

XP_423602_Gallus_BCH ----MDHIAVKEYVLVYFHTLTN---------------------------

XP_002197809_Taeniopygia_BCH ----MDHIAVKEYVLVYFHTLTN---------------------------

XP_001144527_Pan_BCH ----MDHIAVKEYVLVYFHTLTS---------------------------

NP_060156_Homo_BCH ----MDHIAVKEYVLVYFHTLTS---------------------------

XP_001500874_Equus_BCH ----MDHIAVKEYVLVYFHTLTS---------------------------

NP_001039478_Bos_BCH ----MDHIAVKEYVLVYFHTLTS---------------------------

XP_533021_Canis_BCH ----MDHIAVKEYVLVYFHTLTS---------------------------

XP_001365992_Monodelphis_BCH ----MDHIAVKEYVLVYFHTLTS---------------------------

NP_001013219_Rattus_BCH ----MDHIAVKEYVLVYFHTLTS---------------------------

NP_034399_Mus_BCH ----MDHIAVKEYVLVYFHTLTS---------------------------

XP_001104476_Macaca_BCH ----MDHIAVKEYVLVYFHTLTS---------------------------

NP_001004563_Danio_BCH ----MDHITVKEYVMVYFHTLTG---------------------------

CAF93717_Tetraodon_BCH ----MDHITAKEYVMVYFHTLTA---------------------------

ACO13754_Esox_BCH ----MDHITVKEYVMVYFHTLTE---------------------------

NP_001088345_Xenopus_BCH ----MDHVTAKDYVLVYFHTLTG---------------------------

XP_001512063_Ornithorhynchus_B ----MDHIVVKEYVLVYFHTLTS---------------------------

XP_001776374_Physcomitrella_BC ---EMEPLINRPYSMVYFHSAAAL--------------------------

XP_001759372_physcomitrella_BC ---EMEPLINRPYSIVYFHSAASL--------------------------

EEF01185_Populus_BCH ---EFEPLIQKPYTIVYFHSAASL--------------------------

EEE89642_Populus_BCH ---EFEPLIQKPYTIVYFHSAASL--------------------------

EEF38953_Ricinus_BCH ---EFEPLIQKPYTIVYFHSAASL--------------------------

NP_564960_Arabidopsis_BCH ---EFEPVIQKPYSIVYFHSAASL--------------------------

XP_002467159_Sorghum_BCH ---EFEPLIQKPYTIVYFHSAASL--------------------------

ACG47767_Zea_BCH ---EFEPLIQKPYTIVYFHSAASL--------------------------

NP_001064706_Oryza_BCH ---EFEPLIQKPYSIVYFHSAASL--------------------------

XP_002465844_Sorghum_BCH ---EFEPLIQKPYSIVYFHSAASL--------------------------

ACG45534_Zea_BCH ---EFESLIQKPYTIVYFHSVASL--------------------------

ABK24296_Picea_BCH ---EFEPLIQKPYTIVYFHSAAAL--------------------------

EEE58332_Oryza_BCH SLQEFEPLIQKPYSIVYFHSAASL--------------------------

EEE91516_Populus_BCH --ICSE-LPEGPLCIVYMHSTVQK--------------------------

EEF30886_Ricinus_BCH --MCSE-LPEGPFCIVYMHSTVQK--------------------------

ACU17631_Glycine_BCH --ICSE-LPEGPFCIVYMHSTVQK--------------------------

ABD28324_Medicago_BCH --LFSE-LPDGPFCIVYLHSTVTN--------------------------

ABR17649_Picea_BCH --IFTT-VSEGPFCIVYIHTAVQR--------------------------

XP_001754720_Physcomitrella_BC --LHHE-LKPGPYVVVYFHTAVQR--------------------------

NP_001047687_Oryza_BCH --VLPE-IGEREFVVVYVHSLVDR--------------------------

ACG26273_Zea_BCH --VLPE-VGEREFVVVYMHSLVDR--------------------------

ACG34784_Zea_BCH --VLPE-IGDREFVVVYMHSRVDR--------------------------

BAF15465_Oryza_BCH --VTPA-IGDAEFVVVYMHSGVDR--------------------------

EEF31637_Ricinus_BCH --IYPR-LETKPFSVLYVHTGVQR--------------------------

NP_195300_Arabidopsis_BCH --IFPR-LGRKPFAVLYVHTGVQR--------------------------

ACJ85167_Medicago_BCH --IFPK-LGKKKFAVLYIHTGVQR--------------------------

ACU14441_Glycine_BCH --VFPKLMGKRKFAVLYAHTGVQR--------------------------

EEE75109_Populus_BCH --IFPR-LKKKPFSVLYLHTQVQK--------------------------

EEE93960_Populus_BCH --IYPK-LEEKPFSVVYMHTDVQR--------------------------

EEE81211_Populus_BCH --IYPK-LEEKAFSVVYVHTDVQR--------------------------

EEF52804_Ricinus_BCH --IYPK-LEGGPFSVVYLHTNVQR--------------------------

CAN69115_Vitis_BCH --IFSQ-LGEKPFSVVYVHTGVQR--------------------------

XP_001699293_Chlamydomonas_BCH --LDAL--VDEPYVVVWLHTGSSY--------------------------

XP_002168194_Cnidarian_NF1 ----LQPVRGENWELVADFSHTS---------------------------

XP_001744997_Coano_NF1 ----LKPVISGPFELVVDLTQFS---------------------------

AAB59558_human_NF1 ----LKPYYAKPYEIVVDLTHTG---------------------------

XP_692937.3_Danio_NF1 ----LKPYYAKPYELVVDLTHAG---------------------------

XP_002587546_lancelet_NF1 ----LKPFCGKTYEVVLDLTHCG---------------------------

AAB58976_Drosophila_NF1 ----LKPFCHSPFEVVIDFTHTC---------------------------

XP_001196417_Echinoderm_NF1 ----LKPFDKNPFEVVLDCTNFG---------------------------

XP_002576216_tremtoda_NF1 ----LDAYRNKPFEVVIDFTHTS---------------------------

XP_002115170_placozoa_NF1 ----LAPYMNKPWELVIDCTKFG---------------------------

XP_002121778_ciona_NF1 ----LKPYSARKFDIIIDLTHVG---------------------------

NP_013796_Saccharomyces_CRALTR SRAAGHLVETSCTIMDLKGISIS---------------------------

NP_012832_Saccharomyces_CRALTR SRRAGYLIETSCTVLDLKGISLS---------------------------

NP_195629_Arabidopsis_CRALTRIO SIAAKRHIDQSTTILDVQGVGLS---------------------------

NP_036561_Homo_CRALTRIO TTKLGRKVETITIIYDCEGLGLK---------------------------

NP_777635_Homo_CRALTRIO TERLGKKIETIVMIFDCEGLGLK---------------------------

NP_001091144_Xenopus_CRALTRIO SEKVGKRVEDVVMIYDVEGLGLK---------------------------

NP_001093463_Danio_CRALTRIO SERLGRNIESITMVYDCEGLGMK---------------------------

NP_777637_Homo_CRALTRIO TQKLGRKIEMALMVFDMEGLSLK---------------------------

NP_612042.3_Drosophila_CRALTRI SQKHGWRARQLVVFFDMQDVNLKQ--------------------------

NP_001087870_Xenopus_CRALTRIO TKIFGRPISSWTCLVDLEGLNMR---------------------------

NP_001085706_Xenopus_CRALTRIO TKIFGRPISSWTCLVDLEGLNMR---------------------------

NP_001137470_Homo_CRALTRIO TKVFGRPISSWTCLVDLEGLNMR---------------------------

NP_957392_Danio_CRALTRIO TKIFGKPISCWTCLVDLEGLNMR---------------------------

NP_055507_Homo_CRALTRIO TRQLGRPISSWTCLLDLEGLNMR---------------------------

NP_001040875_Caenorhabditis_CR TRKLGTPISSWSLVVDLDGLSMR---------------------------

NP_609028_Drosophila_CRALTRIO AERLEKPVLNWSLLVDLEGLSMR---------------------------

NP_506408_Caenorhabditis_CRALT EKKTN-QQCSVIYIMDLTGIKFDK--------------------------

NP_510554_Caenorhabditis_CRALT EAKTG-KQAWILYVMDITGLQYNK--------------------------

NP_495168_Caenorhabditis_CRALT ERKYG-TQCSVIYILDLEGLKFDP--------------------------

NP_505746_Caenorhabditis_CRALT EAETG-VQCYMHYIFDLEALNFDP--------------------------

CAB78040_Arabidopsis_CRALTRIO DFVAG-GVSTICQVNDLKNSPGP---------------------------

NP_177360_Arabidopsis_CRALTRIO DFSNPEAKSSFVFVSDFRNAPGL---------------------------

NP_013484_Saccharomyces_CRALTR -----NYPASTTILFDLNGFSMS---------------------------

XP_643391_Dict_CRALTRIO -----G-IETCTLIFDMSDFSSK---------------------------

NP_728794_Drosophila_CRALTRIO -----EVTDRLCIVFDLAEFSTS---------------------------

NP_014168_Saccharomyces_CRALTR -----AGQDSLALLIDFKDYPDVPKVPG----------------------

NP_014135_Saccharomyces_CRALTR -----QGVEKITVLVDFKSYKEPGIIT-----------------------

XP_629043_dict_CRALTRIO -----RGHEQIVLLIDFKNYSIR---------------------------

NP_195382_Arabidopsis_CRALTRIO -----KGQKQMSWLIDFTGWSMA---------------------------

BAH19871_Arabidopsis_CRALTRIO -----DNQEQMVWLIDFHGFNMS---------------------------

NP_001088147_Xenopus_CRALTRIO -------GKLLTVVFDMLDCGLS---------------------------

NP_001088926_Xenopus_CRALTRIO -------GKLLTVVFDMLDSGLS---------------------------

NP_689794_Homo_CRALTRIO -------GKPVTVMFDLSETGIN---------------------------

NP_001007294_Danio_CRALTRIO -------GMPLTVVFDMSESGLS---------------------------

NP_729113_Drosophila_CRALTRIO -------LDKITIFMDMTGAGLS---------------------------

NP_000317_Homo_CRALTRIO -------INGFCIIENFKGFTMQQ--------------------------

NP_001080386_Xenopus_CRALTRIO -------INGFCIIENFKGFTMQQ--------------------------

NP_956999_Danio_CRALTRIO -------INGFVLIENFKGFTMQH--------------------------

NP_775790_Homo_CRALTRIO -------INGFILIIDWSNFSFKQ--------------------------

NP_001088883_Xenopus_CRALTRIO -------INGFILIIDWSNFSFKQ--------------------------

NP_001010852_Homo_CRALTRIO -------VNGFVLIIDWSNFTFKQ--------------------------

NP_001020716_Danio_CRALTRIO -------VNGFVLIIDWSNFTFKQ--------------------------

NP_001034288_Homo_CRALTRIO -------VNGIVILADYKGVSLSK--------------------------

NP_997755_Danio_CRALTRIO -------VNGVVILVDYAGVGLSQ--------------------------

NP_000361_Homo_CRALTRIO -------RNGIKAIFDLEGWQFSH--------------------------

NP_724195_Drosophila_CRALTRIO -------IVGGVGIFDLKDLGLEH--------------------------

NP_609120_Drosophila_CRALTRIO -------ICGSVVIIDMEGLPLSH--------------------------

NP_001079459_Xenopus_CRALTRIO -------RNGLIFIYDMINSSYG---------------------------

NP_001081203_Xenopus_CRALTRIO -------RNGLIFIYDMINSTYG---------------------------

NP_572576_Drosophila_CRALTRIO -------RAGLVFIYDMSGSKYS---------------------------

NP_002824_Homo_CRALTRIO -------RNGLVFIYDMCGSNYA---------------------------

NP_724813_Drosophila_CRALTRIO -------ISGYVEIVDMAKMSLSF--------------------------

NP_610507_Drosophila_CRALTRIO -------IAGVVEIIDFTKIPYSL--------------------------

NP_001021496_caenorhabditis_Rh ----SKMAREKGFLVVIDMRGKQ---------------------------

XP_001891671_brugia_RhoGEF ----ADDYKDRGFTIIIDMRKGT---------------------------

NP_009049_Homo_RhoGEF ----SEEVCKRGFTVIVDMRGSK---------------------------

NP_001097996_danio_RhoGEF ----SEDVCKHGFTVIVDMRGSK---------------------------

NP_001019831_Homo_RhoGEF ----SEDVCKRGFTVIIDMRGSK---------------------------

XP_002598613_branchiostoma_Rho ----SEDTKKHGFSVVVDMRGST---------------------------

NP_651960_drosophila_RhoGEF ----SDAAKNLGFTVIIDMRGNGN--------------------------

XP_002578747_schistosoma_RhoGE ----DDKVKRLGFVVIIDMRSGTT--------------------------

XP_001635815_nematostella_RhoG ----SEDECRLGFSIILDMRSCT---------------------------

NP_835224.3_Homo_RhoGEF ----SEKCKARGFTVIVDGRKSQ---------------------------

NP_001085886_xenopus_RhoGEF ----SEKCKARGFTVIVDGRKSQ---------------------------

NP_878301_danio_RhoGEF ----SEKCKARGFTVIVDGRKSQ---------------------------

NP_001106203_Homo_RhoGEF ----SLQDAGIGFILVIDRRRDK---------------------------

NP_055893_Homo_RhoGEF ----SVEAASIGFIVVIDRRRDK---------------------------

NP_001093325_Homo_RhoGEF ----RQNGSDSRFTIILDRRLDT---------------------------

NP_001033984_Drosophila_CRALTR ---DDVAIVNGLISILDLSNVTTG--------------------------

NP_651174_Drosophila_CRALTRIO --LECDNAAISGLIWVVDARDVT---------------------------

NP_496771_Caenorhabditis_CRALT ------RQSGGLFIMDLDGISFSP--------------------------

XP_002110939_trichoplax_RhoGEF ----CQEVKVKGLTVIVDGRSVDWRG---------------------LKP

XP_640612_Dictyostelium_BCH ----MDPVVEEEYVLVYVHTNMN---------------------------

XP_001420825_Ostreococcus_luc_ ----GVTDNPRGYVIVYHHTGGVYG-------------------------

NP_491993_Caenorhabditis_CRALT EKAKESGRPFEQMTLVFDLENITS--------------------------

EFA82125_poly_NF1 ----CKDSMRTPFSMVIDCTLITG--------------------------

XP_638573_Dictyostelium_BCH ---LEQTIQRGNFSIIYFHSNMS---------------------------

NP_871646_Caenorhabditis_CRALT --HYMEYKGCQPLTVFIDMTGTG---------------------------

XP_645940_Dictyostelium_BCH ---LEQIVKGNRYTLLYSHALLK---------------------------

NP_171669_Arabidopsis_CRALTRIO ---ICARMPRGQEKFVAIGDLQG---------------------------

XP_646383_dict_CRALTRIO --RMDEPKGIEQFCFIVDYKDFG---------------------------

XP_001347356_Plasmodium_fal_BC ----LDPIVKENYVLILCETHTN---------------------------

XP_002140404_Cryptosporidium_B ----TKEYVQKDFVLIYCLTRTN---------------------------

NP_569990_Drosophila_CRALTRIO -----ADIAEGDVQIVDIGGYTLR--------------------------

NP_199584_Arabidopsis_CRALTRIO ----GRPITTCVKVLDMTGLKLS---------------------------

NP_508618_Caenorhabditis_CRALT RQTEQETERKMGVIIIMDLDGFSMD-------------------------

XP_001702344_Chlamydomonas_BCH ----LMPVVQQDYVLVVLSLALG---------------------------

XP_646478_dict_CRALTRIO --LIPSQWEIEDLIVIHDYKDCS---------------------------

NP_012390_Saccharomyces_CRALTR LSLLDFTSSDNNYMTQVHDYKGVS--------------------------

XP_642079_dict_CRALTRIO -AHDHSSVHRNGMTIIEDLHKISIFK------------------------

XP_645456_Dictyostelium_BCH ----MDICVNSPYTLVVDMSWAHISN------------------------

XP_001548197_yeast_NF1 ---IASRMWHKPFGILIDATCYN---------------------------

XP_571310_yeast_NF1 ---LERVTEYFDLIIDLTDFSHS---------------------------

NP_593281_Schizosaccharomyces_ --------KDERYTAVFFAHDST---------------------------

NP_001024391_Caenorhabditis_CR ------CLSSLRHIVNMDGYEINP--------------------------

NP_872173_Caenorhabditis_CRALT --KTSSVVCILDLKGLNLMDFMN---------------------------

NP_497717_Caenorhabditis_CRALT ----SADKGPVQFIVIFDLNTVN---------------------------

XP_001173154_Pan_BCH ---RRKMPSLGWLRKCYQQIDRRLRKNLKS----------LIIVHPSWFI

XP_544705_Canis_BCH ---RRKMPSLGWLRKCYQQIDRRLRKNLKS----------LIIVHPSWFI

XP_001374074_Monodelphis_BCH ---RRKMPSLGWLRKCYQQIDRRLRKNLKS----------LIIVHPSWFI

NP_001008239_Mus_BCH ---RRKMPSLGWLRRCYQQIDRRLRKNLKS----------LIIVHPSWFI

EDL84204_Rattus_BCH ---RRKMPSLGWLRRCYQQIDRRLRKNLKS----------LIIVHPSWFI

XP_001100508_Macaca_BCH ---RRKMPSLGWLRKCYQQIDRRLRKNLKS----------LIIVHPSWFI

NP_004321_Homo_BCH ---RRKMPSLGWLRKCYQQIDRRLRKNLKS----------LIIVHPSWFI

XP_001510218_Ornithorhynchus_B ---RRKMPSLGWLRKCYQQIDRRLRKNLKS----------LIIVHPSWFI

XP_413765_Gallus_BCH ---RRKMPSIGWLRKCYQQIDRRLRKNLKS----------LIIVHPSWFI

XP_002195589_Taeniopygia_BCH ---RRKMPSLGWLRKCYQQIDRRLRKNLKS----------LIIVHPSWFI

NP_001088131_Xenopus_BCH ---RRKMPSLGWLRKCYQQIDRRLRKNLKS----------LIIVHPSWFI

CAF98314_Tetraodon_BCH ---RKRMPTLSWLRKCYQQIDRRLRKNLKS----------LIIVHPSWFI

NP_957512_Danio_BCH ---RRKMPSVGWLRKCYQQIDRRLRKNLKS----------LIIVHPSWFI

XP_001366186_Monodelphis_BCH ---RRKMPGLGWMKKCYQMIDRRLRKNLKS----------FIIVHPSWFI

XP_001916938_Equus_BCH ---RRKMPGLGWMKKCYQMIDRRLRKNLKS----------FIIVHPSWFI

NP_056040_Homo_BCH ---RRRMPGLGWMKKCYQMIDRRLRKNLKS----------FIIVHPSWFI

XP_001146885_Pan_BCH ---RRRMPGLGWMKKCYQMIDRRLRKNLKS----------FIIVHPSWFI

NP_001126916_Pongo_abe_BCH ---RRRMPGLGWMKKCYQMIDRRLRKNLKS----------FIIVHPSWFI

NP_001098852_Bos_BCH ---RRKMPGLGWMKKCYQMIDRRLRKNLKS----------FIIVHPSWFI

NP_851993_Mus_BCH ---RRKMPGLGWMKKCYQMIDRRLRKNLKS----------FIIVHPSWFI

Q5BJR4_Rattus_BCH ---RRKMPGLGWMKKCYQMIDRRLRKNLKS----------FIIVHPSWFI

XP_002187337_Taeniopygia_BCH ---RRRMPGLGWMKKCYQMIDRRLRKNLKS----------FIIVHPSWFI

XP_001231886_Gallus_BCH ---RRRMPGLGWMKKCYQMIDRRLRKNLKS----------FIIVHPSWFI

XP_850604_Canis_BCH ---RRKMPGLGWMKKCYQMIDRRLRKNLKS----------FIIVHPSWFI

XP_001515884_Ornithorhynchus_B ---RRKMPGLGWMKKCYQMIDRRLRKNLKS----------FIIVHPSWFI

CAG08427_Tetraodon_BCH ---RRRLPGFTWMKKCYQMIDRRLKKNLKM----------FIIVHPSWFI

NP_001104624_Danio_BCH ---HRRMPGLNWLKRCYQMIDRRLRKNLKS----------FIIVHPSWFI

CAF97194_Tetraodon_BCH ---HRRMPGLGWLKKCYQMIDRRLRKNLKS----------FIILHPSWFI

NP_001133314_Salmo_BCH ---HRRMPGLGWLKKCYQMIDRRLRKNLKS----------FIIVHPSWFI

XP_001374042_Monodelphis_BCH ---RRRMPGIGWLKKCYQMIDRRLRKNLKS----------LIIVHPSWFI

XP_001511480_Ornithorhynchus_B ---RRRMPGIGWLKKCYQMIDRRLRKNLKS----------LIIVHPSWFI

XP_542168_Canis_BCH ---RRRMPGIGWLKKCYQMIDRRLRKNLKS----------LIIVHPSWFI

NP_001125042_Pongo_abe_BCH ---RRRMPGISWLKKCYQMIDRRLRKNLKS----------LIIVHPSWFI

NP_001075898_Bos_BCH ---RRRMPGIGWLKKCYQMIDRRLRKNLKS----------LIIVHPSWFI

NP_848777_Mus_BCH ---RRRMPGIGWLKKCYHMIDRRLRKNLKS----------LIIVHPSWFI

NP_001035280_Rattus_BCH ---RRRMPGIGWLKKCYHMIDRRLRKNLKS----------LIIVHPSWFI

XP_524457_Pan_BCH ---RRRMPGIGWLKKCYQMIDRRLRKNLKS----------LIIVHPSWFI

BAC03859_Homo_BCH ---RRRMPGIGWLKKCYQMIDRRLRKNLKS----------LIIVHPSWFI

XP_001493463_Equus_BCH ---RRRMPGIGWLKKCYQMIDRRLRKNLKS----------LIIVHPSWFI

XP_418214_Gallus_BCH ---RRSMPGLGWLKKCYQMIDRRLRKNLKA----------LIIVHPSWFI

XP_002195707_Taeniopygia_BCH ---RRRMPGLGWLKKCYQMIDRRLRKNLKA----------LIIVHPSWFI

CAF91924_Tetraodon_BCH ---RRKMPGIVWLKRCYQMIDRKLRKNLKC----------LIIVHPTWFI

XP_001343600_Danio_BCH ---RRRMPGISWLKRCYQMIERRLRKNLKC----------LIIAHPTWFI

CAG08009_Tetraodon_BCH ---RSKMPGISWLKKCYQMIDRRLRKNLKS----------LVIAHPTWFI

CAN87813_Danio_BCH ---RSKMPGISWLKKCYQMIDRRLRKNLKS----------LIITHPSWFI

XP_001170701_Pan_BCH ---RAQVPPLSWIRQCYRTLDRRLRKNLRA----------LVVVHATWYV

NP_612122_Homo_BCH ---RAQVPPLSWIRQCYRTLDRRLRKNLRA----------LVVVHATWYV

XP_001106978_Macaca_BCH ---RAQVPPLSWIRQCYRTLDRRLRKNLRA----------LVVVHATWYV

XP_001491310_Equus_BCH ---RAQVPPLSWMRQCYLTLDRRLRKNLRA----------LVVVHATWYV

XP_001928046_Sus_BCH ---RAQVPPLSWLRQCYHTLDGRLRKNLRA----------LVVVHATWYV

NP_001073089_Bos_BCH ---RAQVPPLSWIRQCYHTLDRRLRKNLRA----------LVVVHATWYM

EDL38792_Mus_BCH ---RAQVPPLSWIRQCYRTLDRRLRKNLRA----------LVVVHATWYV

NP_001121659_Rattus_BCH ---RAQVPPLSWIRQCYHTLDRRLRKNLRA----------LVVVHATWYV

XP_540308_Canis_BCH ---RAQVPPLGWMRQCYHTLDRRLRKNLRA----------LVVVHATWYV

XP_001371249_Monodelphis_BCH ---RAQVPPLSWIRQCYRTLDRRLRKNLRA----------LVVVHATWYV

NP_001086594_Xenopus_BCH ---RCKIPPISWIKRCYQATGRRLKKNLKS----------VLILHPTWYV

NP_001121866_Danio_BCH ---RNKMPGIKWLRQCYMSIDRRLRKDLKG----------LFVVHPAWYV

XP_002127893_Ciona_BCH ---RKNLPPLNWLKRCYQMIHRRLRKNLKC----------LVVVHPSWYI

EEC09901_Ixodes_BCH ---RSIMPSFGWLKRCYQMIDRRLRKNLKG----------LYLVHPTFWV

XP_001947520_Acyrthosiphon_BCH ---KDCVPGFSWLKRCYKMIDRKLRKNLKF----------LYLVHPTFWL

EEB18677_Pediculus_BCH ---RSCMPKFKWLKMCYQMIDRKLKKNLKG----------LYLVHPTFWL

XP_315572_Anopheles_BCH ---RGNVPPFPWLKKCYQLLDRRLRKSLRN----------LYMVHPTFWL

XP_001648558_Aedes_BCH ---RGNVPPFPWLKKCYQLLDRRLRKSLKN----------LYMVHPTFWL

ABY20545_Drosophila_mel_BCH ---RRNVPPFPWLKRCYQLLDRRLRKSLKH----------MYLVHPTFWI

XP_974968_Tribolium_BCH ---K--LPAFSWLKKCYQMVGRKLRKNLSH----------LYLVHPTLWI

XP_783795_Strongylocentrotus_B ---SDKIPSLGWMRKCYQMIDRKLRKSLKG----------LYLVHPTTWL

XP_001626140_Nematostella_BCH ---QSNMPALTWMKRFYQHVEGGLKKNMIN----------MFIVHPNLWL

XP_002126187_Ciona_BCH ---KSFYPSTAWLRKSHQQLSHRLRKNIKG----------FYVVQPSFYF

XP_508398_Pan_BCH ---SDNKPSLSWLRDAYREFDRKYKKNIKA----------LYIVHPTMFI

CAM14521_Mus_BCH ---SDNKPSLSWLRDAYREFDRKYKKNIKA----------LYIVHPTMFI

NP_001124902_Pongo_abe_BCH ---SDNKPSLSWLRDAYREFDRKYKKNIKA----------LYIVHPTMFI

XP_001101907_Macaca_BCH ---SDNKPSLSWLRDAYREFDRKYKKNIKA----------LYIVHPTMFI

BAG60756_Homo_BCH ---SDNKPSLSWLRDAYREFDRKYKKNIKA----------LYIVHPTMFI

NP_001101217_Rattus_BCH ---SDNKPSLSWLRDAYREFDRKYKKNIKA----------LYIVHPTMFI

XP_001490021_Equus_BCH ---SDNKPSLSWLRDAYREFDRKYKKNIKA----------LYIVHPTMFI

XP_871999_Bos_BCH ---SDNKPSFSWLRDAYREFDRKYKKNIKA----------LYIVHPTMFI

XP_001369707_Monodelphis_BCH ---SENKPSLSWLRDAYREFDRKYKKNIKA----------LYVVHPTMFI

XP_426422_Gallus_BCH ---SENKPSLSWLRDAYREFDRKYKKNIKA----------LYIVHPTMFI

XP_002198819_Taeniopygia_BCH ---SENKPSLSWLRDAYREFDRKYKKNIKA----------LYIVHPTMFI

NP_001086509_Xenopus_BCH ---SDNKPSLGWLRDAYREFDRKYKKNIKA----------LYIVHPTMFI

NP_001017781_Danio_BCH ---SENKPSLSWLRDAYREFDRKYKKNIKA----------LYIVHPTMFI

EDL04450_Mus_BCH ---SQNKPSLGWLQNTYKEFDRKYKKNLKA----------LYVVHPTSLI

NP_001004242_Rattus_BCH ---SQNKPSLGWLQNAYKEFDRKYKKNLKA----------LYVVHPTSLI

CAQ06715_Homo_BCH ---SRNKPSLGWLQSAYKEFDRKYKKNLKA----------LYVVHPTSFI

XP_416463_Gallus_BCH ---SLNKPSLKWLQTAYKEFDRKYKKNLKA----------LYVVHPTNFI

NP_001085153_Xenopus_BCH ---SRNKPSLSWLQSAYKEFDRKYKKNLKA----------LYVVHPTNFI

EEB11750_Pediculus_BCH ---SRNKPTLSWLWQAYRAFDRKYKKNLKA----------LYLVHPTNFI

XP_001950301_Acyrthosiphon_BCH ---SKNKPTLSWLWQAYRAFDRKYKKNLKA----------LYLVHPTGFI

XP_001604462_Nasonia_BCH ---SKNKPPLSWLWQAYKAFDRKYKKNLKA----------LYLVHPTNFI

XP_624226_Apis_BCH ---SKNKPPLSWLWQAYKAFDRKYKKNLKA----------LYLVHPTNFI

XP_002121397_Ciona_BCH ---SSNKPSFSWLREAYKEFDRKYKKNLKS----------LYLVHPTTFI

XP_970580_Tribolium_BCH ---SKNKPSLRWLVQAYKAFDRKYKKNLKA----------LYLVHPTGFL

XP_002160831_Hydra_BCH ---SINKPSFKWLLQVYKELDRKYKKNLKA----------FYIVHPSNFI

NP_001022390_Caenorhabditis_el ---SHNKPPVRWLFQAYKQLDRRFKKNLKA----------LYVVHPTRFI

XP_001893352_Brugia_BCH ---SNNKPSLKWLLQAYSILDRKYKKNLKA----------LYLVHPTRFI

XP_002117739_Trichoplax_BCH ---SKNKPSYTWLIQAYRAFDRKYKKNLKA----------LYIVHASNFI

XP_002572184_Schistosoma_BCH ---NKNRPKFKWLVQAYRTFGRNFRKNLKT----------LYIVHPTTGI

EEC10256_Ixodes_BCH ---SKNKPSLGWLWTAFRTFDRRYKKNLKA----------LYLVHPTSFL

XP_001746114_Monosiga_BCH TPLAQNKPALSWIRRVYSSFDRRYKKNLKA----------LYVVHATTFV

XP_001835505_Coprinopsis_BCH -----HSPGWNWIWKAYRSLSRKYRKNLKQ----------LYIVHSSFFS

XP_001876797_Laccaria_BCH -----HTPGWNWVWKAYRSLSRKYRKNLKQ----------LYIVHSSFFS

XP_760803_Ustilago_BCH -----HRPGWSWIWRAYRRLDRRFRKNLKK----------LYIVHPTLFT

XP_001843366_Culex_BCH ---DNNKPSLQFLWNSYKELDRSFKKNLKK----------LYVVHPTTFI

XP_001659702_Aedes_BCH ---DNSKPSLQFLWNSYKELDRSFKKNLKK----------LYVVHPTTFI

NP_648552_Drosophila_mel_BCH ---EDNKPSAQFLWNSYKELDRNFRKNLKT----------LYVVHPTWFI

XP_001182784_Strongylocentrotu ---ADNQPELSWLKDVYHMVDNRYRKNLRA----------MYIVHPTFWS

XP_001622394_Nematostella_BCH ---EENQPPMSFLKELYHIVDNKYRRNLKA----------FYIVHPTVWA

XP_396878_Apis_BCH ---SNNYPSLHWLREVYNVLPYKYKKNLKH----------FYIIHPTFWT

XP_001815509_Tribolium_BCH ---SSNYPSFSWLREVYNVLPYKYKKNLKA----------FYIVHPTFWT

NP_724599_Drosophila_mel_BCH ---TNNYPSLHWLREVYSVLPYKYKKNLKA----------FYIVHPTFWT

NP_724597_Drosophila_mel_BCH ---TNNYPSLHWLREVYSVLPYKYKKNLKA----------FYIVHPTFWT

XP_001867337_Culex_BCH ---SNNYPSLQWLKDVYSILPYKYKKNLKA----------FYIVHPTFWT

XP_001688494_Anopheles_BCH ---SNNYPSLQWLKDVYSILPYKYKKNLKA----------FYIVHPTFWT

XP_001660566_Aedes_BCH ---SNNYPSLQWLKDVYSILPYKYKKNLKA----------FYIVHPTFWT

EEB16863_Pediculus_BCH ---AANHPSFSWLREVYNILPYKYKKNLKA----------FYIVHPTFWT

ACO15636_Caligus_BCH ---RDNIPSYGWIKEIYNTLSYRYKKNLKA----------FYIVRPTLWT

XP_002112464_Trichoplax_BCH ---AEQRPPANFLKLVYQTLDPKYHKNLKA----------FYVVHPSWWL

XP_423602_Gallus_BCH ---DYNQLDSNFLKKLYDVVDAKYKRNLKA----------LYFVHPTFRS

XP_002197809_Taeniopygia_BCH ---DYNQLDSNFLKKLYDVVDAKYKRNLKA----------LYFVHPTFRS

XP_001144527_Pan_BCH ---EYNHLDSDFLKKLYDVVDVKYKRNLKA----------VYFVHPTFRS

NP_060156_Homo_BCH ---EYNHLDSDFLKKLYDVVDVKYKRNLKA----------VYFVHPTFRS

XP_001500874_Equus_BCH ---EYNHLDSDFLKKLYDVVDVKYKRNLKA----------VYFVHPTFRS

NP_001039478_Bos_BCH ---EYNHLDSDFLKKLYDVVDVKYKRNLKA----------VYFVHPTFRS

XP_533021_Canis_BCH ---EYNHLDSDFLKKLYDVVDVKYKRNLKA----------VYFVHPTFRS

XP_001365992_Monodelphis_BCH ---EYNHLDSDFLKKLYDVVDIKYKRNLKA----------VYFVHPTFRS

NP_001013219_Rattus_BCH ---EYNHLDSDFLKKLYDVVDIKYKRNLKA----------VYFVHPTFRS

NP_034399_Mus_BCH ---DYNHLDSDFLKKLYDVVDIKYKRNLKA----------VYFVHPTFRS

XP_001104476_Macaca_BCH ---EYNHLDSDFLKKLYDVVDVKYKRNLKA----------VYFVHPTFRS

NP_001004563_Danio_BCH ---EHNHLDTDFLKKLYDIVDAKFKKNLRA----------FYFVHPTFRS

CAF93717_Tetraodon_BCH ---EHNHLHSDFLRNLHDIVDYKFKKNLKA----------FYFVHPTFRS

ACO13754_Esox_BCH ---ERNHLDSEFLKKLYEIVDAKFKNNLKA----------FYFVHPSFRS

NP_001088345_Xenopus_BCH ---EHNHLDSDFLKNMYDIIDVKYKKNLKA----------LYFVHPTFRS

XP_001512063_Ornithorhynchus_B ---DYNRLDSDFLKKLYDVVDAKYKRNLKA----------VYFVHPTFRS

XP_001776374_Physcomitrella_BC ---SIQ-PDLGWVKRLHQILGRRHKHNLHA----------IYILHPTIGL

XP_001759372_physcomitrella_BC ---SIQ-PDFGWVKRLHQILGRRHKHNLHA----------IYILHPTLGL

EEF01185_Populus_BCH ---QFQ-PNMGWMRRLQQILGRKHQRNLHA----------IYVLHPNFHL

EEE89642_Populus_BCH ---QVQ-PDLGWIRRLQQILTRKHQRNLHA----------IYVLHPNFHL

EEF38953_Ricinus_BCH ---QLQ-PDLGWMRRLQQILGRKHQRNLHA----------IYVLHPTFHL

NP_564960_Arabidopsis_BCH ---QVQ-PDLGWMKRLEQILGRKHQRNLQA----------IYVLHPTFHL

XP_002467159_Sorghum_BCH ---QPQ-PDLGFMKRLQQILGRKHQRNLHA----------IYVLHPTLGL

ACG47767_Zea_BCH ---QPQ-PDLGFMKRLQQILGRKHQRNLHA----------IYVLHPTLGL

NP_001064706_Oryza_BCH ---QPQ-PDLGFMKRLQQILGRKHQRNLHA----------IYVLHPTLGL

XP_002465844_Sorghum_BCH ---QPQ-PDLGFMKRLQQILGRKHQKNLHT----------IYILHPTLGL

ACG45534_Zea_BCH ---QPQ-PDLGFMKRLQQILGRKHKKNLHT----------IYILHPTLGL

ABK24296_Picea_BCH ---EMQ-PDLGWMKRLQQILGRKHKRNLHA----------IYVLHPTLGL

EEE58332_Oryza_BCH ---QVR-PDLGFMKRLQQILGRKHQRNLHVGISYDH--TAIYVLHPTLGL

EEE91516_Populus_BCH ---EDNSPGVTILRWIYEELPAGIKDRLQT----------VYFIHPGLRS

EEF30886_Ricinus_BCH ---EDNSPGITILRWIYEELPADYKNRLQV----------VYFIHPGLRS

ACU17631_Glycine_BCH ---EDNSPGITILSWIYEELPADFKDRLQT----------VYFIHPGFRS

ABD28324_Medicago_BCH ---EDNSPGMTILRWIYEDLPDEFKDRLQT----------LYFIHPGLRS

ABR17649_Picea_BCH ---EENSPGLSIIRWIYEDLPTDYKQRLQL----------VYFLHPGILS

XP_001754720_Physcomitrella_BC ---NDNSPGLWALRDLYEILPKQLKHGLQA----------VYVVHPGLRF

NP_001047687_Oryza_BCH ---GDNFPGVAAIRAAYEALPAAAKERLRA----------VYFVHPGFQA

ACG26273_Zea_BCH ---GDNFPGLGAIRAAYECMPAAAKEKLRA----------VYFVHPGIQA

ACG34784_Zea_BCH ---GHNFPGVGAIRGAYETLPAAAKERLRA----------VYFVHPALQS

BAF15465_Oryza_BCH ---RENFPGVGAVRTAYESMPAAVRERLHA----------VYFLHPGLQS

EEF31637_Ricinus_BCH ---SENFPGISALRSVYDSIPIKVKENLQA----------LYFLHPGLQA

NP_195300_Arabidopsis_BCH ---SENFPGISALRAIYDAIPVNVRDNLQE----------VYFLHPGLQS

ACJ85167_Medicago_BCH ---SENFAGISSLRSVYDAIPANVKENLEA----------VYFIHPGLQA

ACU14441_Glycine_BCH ---SENFPGISGLRWIYDAIPANVKENLEA----------VYFIHPGLQA

EEE75109_Populus_BCH ---SEDFPGISALRSIYDVIPINARDNLQA----------IYFVHPSLQA

EEE93960_Populus_BCH ---SENLPGISTLRSIYEDIPINVKNHLES----------IYFLHPGLQA

EEE81211_Populus_BCH ---SENFPGISTLRSIYDDIPMNVKSHLES----------VYFLHPGLQA

EEF52804_Ricinus_BCH ---SENFPGISALRSIYDAIPINVKNNLEA----------VYFVHPAIQD

CAN69115_Vitis_BCH ---SDNFPGISVLRSIYEAIPINVKDHIEA----------VYFLHPGLQA

XP_001699293_Chlamydomonas_BCH ---WNNCPSLAWLWRTYERLPCKYRTNLAR----------LFVVHCDLPL

XP_002168194_Cnidarian_NF1 ---LVNRFKADTLIKAFTIIPESQLASLQL----------IYLYNINHSL

XP_001744997_Coano_NF1 ---RANEPSGPALEQFAGLIPPSLTQNLRR----------VYVLNAPR--

AAB59558_human_NF1 ---PSNRFKTDFLSKWFVVFPGFAYDNVSA----------VYIYNCNSWV

XP_692937.3_Danio_NF1 ---PSNRFKTDFLSKWFVVFPGFAYENVAA----------IYIYNCNTWV

XP_002587546_lancelet_NF1 ---PDNRFKTDFLSKCFYVFPEVAYHNILA----------VYLYNCNTWV

AAB58976_Drosophila_NF1 ---SDNRFRTEFLQKWFYVLPTVAYENVHA----------VYIYNCNSWV

XP_001196417_Echinoderm_NF1 ---PDNRFKPQVLSRG-VRFPEHVTQNLSA----------VYVYNCNSRL

XP_002576216_tremtoda_NF1 ---VENRFKNDLLNKWANIIGPVLREYLVA----------AYIYNCNSWV

XP_002115170_placozoa_NF1 ---PPNQLKGNFISKWFHVFPNQATDNLQA----------VYIYNCNKWV

XP_002121778_ciona_NF1 ---AKNRFRGQYLPKWLVLYPQQIYKNLDT----------VYVYNPNTWV

NP_013796_Saccharomyces_CRALTR ---SAYSVMS-YVREASYISQNYYPERMGK----------FYIINAPFGF

NP_012832_Saccharomyces_CRALTR ---NAYHVLS-YIKDVADISQNYYPERMGK----------FYIIHSPFGF

NP_195629_Arabidopsis_CRALTRIO ---NFNKAAKDLLQSIQKIDNDNYPETLNR----------MFIINAGCGF

NP_036561_Homo_CRALTRIO ---HLWKPAVEAYGEFLCMFEENYPETLKR----------LFVVKAPKLF

NP_777635_Homo_CRALTRIO ---HFWKPLVEVYQEFFGLLEENYPETLKF----------MLIVKATKLF

NP_001091144_Xenopus_CRALTRIO ---HLWKPAVELYGEILQMFEDNYPEALKR----------LFVIKAPKLF

NP_001093463_Danio_CRALTRIO ---HLYKPAIETYGEVLTMFEDNYPEGLKR----------LFVIKAPKLF

NP_777637_Homo_CRALTRIO ---HLWKPAVEVYQQFFSILEANYPETLKN----------LIVIRAPKLF

NP_612042.3_Drosophila_CRALTRI ---YAWRPAAECVISTVKQYEANFPELLKM----------CYIINAPKLF

NP_001087870_Xenopus_CRALTRIO ---HLWRPGVKALLRIIEVVEANYPETLGR----------LLILRAPRVF

NP_001085706_Xenopus_CRALTRIO ---HLWRPGVKALLRIIEVVEANYPETLGR----------LLILRAPRVF

NP_001137470_Homo_CRALTRIO ---HLWRPGVKALLRIIEVVEANYPETLGR----------LLILRAPRVF

NP_957392_Danio_CRALTRIO ---HLWRPGIKALLRMIEVVGANYPETLGR----------LLILRAPRVF

NP_055507_Homo_CRALTRIO ---HLWRPGVKALLRMIEVVEDNYPETLGR----------LLIVRAPRVF

NP_001040875_Caenorhabditis_CR ---HLWRPGVQCLLKIIEIVEANYPETMGQ----------VLVVRAPRVF

NP_609028_Drosophila_CRALTRIO ---HLWRPGIKALLNIIETVERNYPETMGR----------VLVVRAPRVF

NP_506408_Caenorhabditis_CRALT ---RTITLLTGGLSAISAFMAEHYVELVHS----------FVLVNVPAFI

NP_510554_Caenorhabditis_CRALT ---KLYDLVTGSMKSLADFMADHYVEMIKY----------FVPVCVPSFA

NP_495168_Caenorhabditis_CRALT ---ALISIVTGPYRILWASVYTAYPEWINT----------LFLINAPSFM

NP_505746_Caenorhabditis_CRALT ---TLLGVVNGPFRVSWQLVGQHYREFIDK----------FIVINSPSYI

CAB78040_Arabidopsis_CRALTRIO ----GKTELRLATKQALHLLQDNYPEFVSK----------QIFINVPWWY

NP_177360_Arabidopsis_CRALTRIO ----GKRALWQFIRRAVKQFEDNYPEFAAK----------ELFINVPWWY

NP_013484_Saccharomyces_CRALTR ------NMDYAPVKFLITCFEAHYPESLGH----------LLIHKAPWIF

XP_643391_Dict_CRALTRIO ------NMDYPLVKFMVELFQKFYPESLQK----------CLILNAPWIF

NP_728794_Drosophila_CRALTRIO ------CMDYQLVQNLIWLLGKHFPERLGV----------CLIINSPGLF

NP_014168_Saccharomyces_CRALTR ---NSKIPPIGVGKEVLHILQTHYPERLGK----------ALLTNIPWLA

NP_014135_Saccharomyces_CRALTR ----DKAPPISIARMCLNVMQDHYPERLAK----------CVLINIPWFA

XP_629043_dict_CRALTRIO -----NSPPMSVSKYVLQILSDHYPERLGN----------AFLVETPFIF

NP_195382_Arabidopsis_CRALTRIO -----VNPPMKTTREIIHILQNYYPERLGI----------AFLYNPPRLF

BAH19871_Arabidopsis_CRALTRIO -----HIS-LEVSRETAHVLQEHYPERLGL----------AIVYNPPKIF

NP_001088147_Xenopus_CRALTRIO ------NVDMDFVRFIINSFKTYYPRYLSK----------IVVYEMPWIL

NP_001088926_Xenopus_CRALTRIO ------NIDMDFVRFVINSFKTYYPRYLSK----------MVVFEMPWIL

NP_689794_Homo_CRALTRIO ------SIDMDFVRFIINCFKVYYPKYLSK----------IVIFDMPWLM

NP_001007294_Danio_CRALTRIO ------NIDMDFVKYIISCFKVYYPKFLSK----------MIMYEMPWIM

NP_729113_Drosophila_CRALTRIO ------NLDMGFIKSIIGVFETKYPYVPNY----------ILVHDLPFLL

NP_000317_Homo_CRALTRIO ----AASLRTSDLRKMVDMLQDSFPARFKA----------IHFIHQPWYF

NP_001080386_Xenopus_CRALTRIO ----ASGIKPSELKKMVDMLQDSFPARFKA----------VHFIHQPWYF

NP_956999_Danio_CRALTRIO ----ASGIKHTELKKMVDMLQDSFPARFKA----------VHVIHQPWYF

NP_775790_Homo_CRALTRIO ----ASKLTPSILKLAIEGLQDSFPARFGG----------VHFVNQPWYI

NP_001088883_Xenopus_CRALTRIO ----ASKLTPSILRLAIEGLQDSFPARFGG----------VHFVNQPWYI

NP_001010852_Homo_CRALTRIO ----ASKLTPSMLRLAIEGLQDSFPARFGG----------IHFVNQPWYI

NP_001020716_Danio_CRALTRIO ----ASKLTPSMLRLAIEGLQDSFPARFGG----------IHFVNQPWYI

NP_001034288_Homo_CRALTRIO ----ASHFGPFIAKKVIGILQDGFPIRIKA----------VHVVNEPRIF

NP_997755_Danio_CRALTRIO ----ASNPGPLLAKKVVGILQDGFPIRIKA----------VNIINEPRIF

NP_000361_Homo_CRALTRIO ----AFQITPSVAKKIAAVLTDSFPLKVRG----------IHLINEPVIF

NP_724195_Drosophila_CRALTRIO ----ILHLSPSVAQKMIALLVTSMPIRTSA----------LHIVNQNWVF

NP_609120_Drosophila_CRALTRIO ----ITQFTPSFAAMLLDYIQECICMRLKA----------VHIVNNSYIF

NP_001079459_Xenopus_CRALTRIO ------NFDYELCVKILNLLKGAFPARLKC----------VFIVSSPLWF

NP_001081203_Xenopus_CRALTRIO ------NFDYELCVKILNLLKGAFPARLKC----------VFIVSSPLWF

NP_572576_Drosophila_CRALTRIO ------NFDYDLSQKILTLLKGGYPARLKK----------VLIVTAPLWF

NP_002824_Homo_CRALTRIO ------NFELDLGKKVLNLLKGAFPARLKK----------VLIVGAPIWF

NP_724813_Drosophila_CRALTRIO ----LAQLDFTLIKRMGIFAEKAQPTRLKG----------VHLINCPKEG

NP_610507_Drosophila_CRALTRIO ----LLQFDPGMFKRMNAFLEHGIPANLVA----------THIVNASRET

NP_001021496_caenorhabditis_Rh --------TWTNVRHILKALSSIESSSTVQ----------VFIIKPEKFW

XP_001891671_brugia_RhoGEF --------TWNNVKPILKCIQEHFPAEVYA----------VLIIKPDKFW

NP_009049_Homo_RhoGEF ---------WDSIKPLLKILQESFPCCIHV----------ALIIKPDNFW

NP_001097996_danio_RhoGEF ---------WDSIKPLLKILQESFPCCIHI----------ALIIKPDNFW

NP_001019831_Homo_RhoGEF ---------WDLIKPLLKTLQEAFPAEIHV----------ALIIKPDNFW

XP_002598613_branchiostoma_Rho ---------WSSVKPLLKALQDCFPGNIHM----------AHIIKPDNFW

NP_651960_drosophila_RhoGEF --------CSTNVKTILKVLQEHFSANIHN----------VVIIKPDNFW

XP_002578747_schistosoma_RhoGE ---------WHSVKPILKVLEECLGQDVTL----------TFIIKPDKFL

XP_001635815_nematostella_RhoG ---------WSTAKPVLRSLQECFPEKIHS----------VYIIKPEGFW

NP_835224.3_Homo_RhoGEF ---------WNVVKTVVVMLQNVVPAEVSL----------VCVVKPDEFW

NP_001085886_xenopus_RhoGEF ---------WNVVKTVVLMLQNVVPAEVSL----------VCVVKPDEFW

NP_878301_danio_RhoGEF ---------WNIVKTVVLMLQNVIPAEVSL----------VCVVKPDEFW

NP_001106203_Homo_RhoGEF ---------WTSVKASVLRIAASFPANLQL----------VLVLRPTGFF

NP_055893_Homo_RhoGEF ---------WSSVKASLTRIAVAFPGNLQL----------IFILRPSRFI

NP_001093325_Homo_RhoGEF ---------WSSLKISLQKISASFPGNLHL----------VLVLRPTSFL

NP_001033984_Drosophila_CRALTR ---HFLQMSPSFAKKMTVFQEEALPLRPQG----------IHFINTPNGF

NP_651174_Drosophila_CRALTRIO -MEQMMQYDPFLLKKAFALVDQCIPLRFVE----------IHMINMRKEG

NP_496771_Caenorhabditis_CRALT ---KLISVLTGPYRIMWGTLFDHYPQLLQK----------IIIVNAPSFV

XP_002110939_trichoplax_RhoGEF LLNILEEAFLDTIAKVFVIKSDSFWKRGKAMITQMKTELNMQVVSISNLV

XP_640612_Dictyostelium_BCH ---NSNKPSMAWMKKVYTIFNRKYKKNLKG----------LYIVHPTTWI

XP_001420825_Ostreococcus_luc_ -----APPSLDFIRKLHVALGPQHRDTLKT----------VFVVHATAIL

NP_491993_Caenorhabditis_CRALT -AHFSCKQFASSFTTLVSLFQDHYPLFLRK----------ILIIRAPEMA

EFA82125_poly_NF1 ----EHNIPMSWCSTWMRHFPQQLTDNLKT----------IYIVSPNHIL

XP_638573_Dictyostelium_BCH ---SQQSPDLSWLKKLLEIFELKYNNYLKD----------FNIVHPTFLL

NP_871646_Caenorhabditis_CRALT ----LKNMSFDAMKFIIHSSKYYYPNSIES----------ILIFENPAIL

XP_645940_Dictyostelium_BCH ---QESTPDKSWLNSFYQMLPRNYKKNLKN----------LYILHPSGWL

NP_171669_Arabidopsis_CRALTRIO --WGYSNCDIRGYLAALSTLQDCYPERLGK----------LYIVHAPYIF

XP_646383_dict_CRALTRIO ----SGNMDMKTNLEAMHFLLDHCPERMGQS---------LFLDPPALFW

XP_001347356_Plasmodium_fal_BC ---WLTDAVYAYAKQWYDTLPRKYKKNLKN----------LYLVHSGFLS

XP_002140404_Cryptosporidium_B ---VLSDKSGGFLQAFYGLLPKDFKKNLKK----------VIMFHYGISN

NP_569990_Drosophila_CRALTRIO ---HLAYVSIFVLRVYMKFLQEAYPSRLQA----------MHVINCPTYL

NP_199584_Arabidopsis_CRALTRIO -----ALSQIKLVTIISTIDDLNYPEKTNT----------YYVVNAPYIF

NP_508618_Caenorhabditis_CRALT ---LLYTPTLKVYMSLLTMLQNIFPDFARR----------IYIINCPAMM

XP_001702344_Chlamydomonas_BCH --VKASTVSAAWAIGAYKSLAKPYRKNVKH----------IVLVQPSAWA

XP_646478_dict_CRALTRIO -FFRMDPRIKQASNQTIQTLQNNYPEFLAR----------KFFINIPWLM

NP_012390_Saccharomyces_CRALTR -VWRMDSDIKNCSKTVIGIFQKYYPELLYA----------KYFVNVPTVF

XP_642079_dict_CRALTRIO --HFDSRLQDFLKKNKLNDMQDVFIGRIQK----------IYILNPPWVL

XP_645456_Dictyostelium_BCH ---DLKKAIFTHLPKLAEIFSRKYKKNIDK----------IFIVHPSAYT

XP_001548197_yeast_NF1 ---GQNEPADALFRRLDLLTPTELSKQLSR----------VYVYNMNSAF

XP_571310_yeast_NF1 -----TELPMVWLKKSIQLCPSGILSCLNT----------LVFYNPNSYA

NP_593281_Schizosaccharomyces_ -----VFSYLGLSLKAYYGMDYYLHKNVKA----------VYVIHTDWMS

NP_001024391_Caenorhabditis_CR --FTMVFVTSGTLAYYSQLFHFENYPELVTP---------VDMVNIAKWI

NP_872173_Caenorhabditis_CRALT ----PLSGPAQLARLVVQVWAEYFSEHLCK----------LLLINPPGII

NP_497717_Caenorhabditis_CRALT --ITDYVNPMSGYMKLWQIRSELWQDWFPEMVQR------IYLTNPPRLL

XP_001173154_Pan_BCH RTL-------------LAVTRPFI--------------------------

XP_544705_Canis_BCH RTL-------------LAVTRPFI--------------------------

XP_001374074_Monodelphis_BCH RTL-------------LAITRPFI--------------------------

NP_001008239_Mus_BCH RTL-------------LAVTRPFI--------------------------

EDL84204_Rattus_BCH RTL-------------LAVTRPFI--------------------------

XP_001100508_Macaca_BCH RTL-------------LAVTRPFI--------------------------

NP_004321_Homo_BCH RTL-------------LAVTRPFI--------------------------

XP_001510218_Ornithorhynchus_B RTL-------------LAITRPFI--------------------------

XP_413765_Gallus_BCH RTL-------------LAITKPFI--------------------------

XP_002195589_Taeniopygia_BCH RTL-------------LAITKPFI--------------------------

NP_001088131_Xenopus_BCH RTL-------------LAITKPFI--------------------------

CAF98314_Tetraodon_BCH RTL-------------LALTKPFIRSEVALPTRQSENAWEAWPDSDPHSS

NP_957512_Danio_BCH RTL-------------LALTKPFI--------------------------

XP_001366186_Monodelphis_BCH RTI-------------LAVTRPFI--------------------------

XP_001916938_Equus_BCH RTI-------------LAVTRPFI--------------------------

NP_056040_Homo_BCH RTI-------------LAVTRPFI--------------------------

XP_001146885_Pan_BCH RTI-------------LAVTRPFI--------------------------

NP_001126916_Pongo_abe_BCH RTI-------------LAATRPFI--------------------------

NP_001098852_Bos_BCH RTI-------------LAVTRPFI--------------------------

NP_851993_Mus_BCH RTI-------------LAVTRPFI--------------------------

Q5BJR4_Rattus_BCH RTI-------------LAVTRPFI--------------------------

XP_002187337_Taeniopygia_BCH RTI-------------LAVTRPFI--------------------------

XP_001231886_Gallus_BCH RTI-------------LAVTRPFI--------------------------

XP_850604_Canis_BCH RTI-------------LAVTRPFI--------------------------

XP_001515884_Ornithorhynchus_B RTI-------------LAVTRPFI--------------------------

CAG08427_Tetraodon_BCH RTL-------------LGITRPFI--------------------------

NP_001104624_Danio_BCH RTI-------------QAITKPFI--------------------------

CAF97194_Tetraodon_BCH RTV-------------LAITKPFI--------------------------

NP_001133314_Salmo_BCH RTI-------------LALTRPFI--------------------------

XP_001374042_Monodelphis_BCH RTV-------------LAISRPFI--------------------------

XP_001511480_Ornithorhynchus_B RTV-------------LAISRPFI--------------------------

XP_542168_Canis_BCH RTV-------------LAISRPFI--------------------------

NP_001125042_Pongo_abe_BCH RTV-------------LAISRPFI--------------------------

NP_001075898_Bos_BCH RTV-------------LAISRPFI--------------------------

NP_848777_Mus_BCH RTV-------------LAISRPFI--------------------------

NP_001035280_Rattus_BCH RTV-------------LAISRPFI--------------------------

XP_524457_Pan_BCH RTV-------------LAISRPFI--------------------------

BAC03859_Homo_BCH RTV-------------LAISRPFI--------------------------

XP_001493463_Equus_BCH RTV-------------LAISRPFI--------------------------

XP_418214_Gallus_BCH RTV-------------LAISRPFI--------------------------

XP_002195707_Taeniopygia_BCH RTV-------------LAISRPFI--------------------------

CAF91924_Tetraodon_BCH RTV-------------LAISRPFI--------------------------

XP_001343600_Danio_BCH RTV-------------LAISRPFV--------------------------

CAG08009_Tetraodon_BCH RTV-------------LAISRPFI--------------------------

CAN87813_Danio_BCH RTV-------------IAISKPFI--------------------------

XP_001170701_Pan_BCH KAF-------------LALLRPFI--------------------------

NP_612122_Homo_BCH KAF-------------LALLRPFI--------------------------

XP_001106978_Macaca_BCH KAF-------------LALLRPFI--------------------------

XP_001491310_Equus_BCH KAF-------------LALLRPFI--------------------------

XP_001928046_Sus_BCH KAF-------------LALLRPFI--------------------------

NP_001073089_Bos_BCH KAF-------------LALLRPFI--------------------------

EDL38792_Mus_BCH KAF-------------LALVRPFI--------------------------

NP_001121659_Rattus_BCH KAF-------------LALVRPFI--------------------------

XP_540308_Canis_BCH KAF-------------LALLRPFI--------------------------

XP_001371249_Monodelphis_BCH KAF-------------LVMLRPFI--------------------------

NP_001086594_Xenopus_BCH RAL-------------LAITRPFI--------------------------

NP_001121866_Danio_BCH RAL-------------ITVIKPFI--------------------------

XP_002127893_Ciona_BCH RFL-------------IGFFRPFI--------------------------

EEC09901_Ixodes_BCH KTI-------------VIMTRPFV--------------------------

XP_001947520_Acyrthosiphon_BCH KTL-------------VLMIKPFV--------------------------

EEB18677_Pediculus_BCH KTL-------------VLMTKPFI--------------------------

XP_315572_Anopheles_BCH KSV-------------VWMARPFI--------------------------

XP_001648558_Aedes_BCH KSV-------------VWMARPFI--------------------------

ABY20545_Drosophila_mel_BCH KSL-------------VWMARPFV--------------------------

XP_974968_Tribolium_BCH KTM-------------LFMAKPFI--------------------------

XP_783795_Strongylocentrotus_B KAI-------------VKLTKPFI--------------------------

XP_001626140_Nematostella_BCH KTV-------------VRFAKAFV--------------------------

XP_002126187_Ciona_BCH KTL-------------INFTRMFI--------------------------

XP_508398_Pan_BCH KTL-------------LILFKP----------------------------

CAM14521_Mus_BCH KTL-------------LILFKP----------------------------

NP_001124902_Pongo_abe_BCH KTL-------------LILFKP----------------------------

XP_001101907_Macaca_BCH KTL-------------LILFKP----------------------------

BAG60756_Homo_BCH KTL-------------LILFKP----------------------------

NP_001101217_Rattus_BCH KTL-------------LILFKP----------------------------

XP_001490021_Equus_BCH KTL-------------LILFKP----------------------------

XP_871999_Bos_BCH KTL-------------LILFKP----------------------------

XP_001369707_Monodelphis_BCH KTL-------------LILFKP----------------------------

XP_426422_Gallus_BCH KTL-------------LILFKP----------------------------

XP_002198819_Taeniopygia_BCH KTL-------------LILFKP----------------------------

NP_001086509_Xenopus_BCH KTL-------------LILFKP----------------------------

NP_001017781_Danio_BCH RTI-------------LILFKP----------------------------

EDL04450_Mus_BCH KAL-------------WNIFKP----------------------------

NP_001004242_Rattus_BCH KAL-------------WNIFKP----------------------------

CAQ06715_Homo_BCH KVL-------------WNILKP----------------------------

XP_416463_Gallus_BCH KIL-------------WNIFKP----------------------------

NP_001085153_Xenopus_BCH KVL-------------WNIFKP----------------------------

EEB11750_Pediculus_BCH RLV-------------WQVFRA----------------------------

XP_001950301_Acyrthosiphon_BCH KVV-------------WQLFRA----------------------------

XP_001604462_Nasonia_BCH RIV-------------WQLFKP----------------------------

XP_624226_Apis_BCH RIV-------------WQIFKP----------------------------

XP_002121397_Ciona_BCH RIL-------------MNVFKP----------------------------

XP_970580_Tribolium_BCH KFV-------------SQIFRP----------------------------

XP_002160831_Hydra_BCH KAA-------------FNIFYP----------------------------

NP_001022390_Caenorhabditis_el RII-------------FSLFKG----------------------------

XP_001893352_Brugia_BCH RIV-------------WSIFKP----------------------------

XP_002117739_Trichoplax_BCH KVM-------------FTLLRP----------------------------

XP_002572184_Schistosoma_BCH KIL-------------WTLFRP----------------------------

EEC10256_Ixodes_BCH KIL-------------YQLFRP----------------------------

XP_001746114_Monosiga_BCH KTI-------------LTLCRP----------------------------

XP_001835505_Coprinopsis_BCH KML-------------FSLAGA----------------------------

XP_001876797_Laccaria_BCH KML-------------FSLAGA----------------------------

XP_760803_Ustilago_BCH KSL-------------MRVVTTGSY-------------------------

XP_001843366_Culex_BCH KMV-------------WFFFKPVI--------------------------

XP_001659702_Aedes_BCH KMV-------------WFFFKPVI--------------------------

NP_648552_Drosophila_mel_BCH RVI-------------WNFFSPFI--------------------------

XP_001182784_Strongylocentrotu KLV-------------TWYFTTFTAS------------------------

XP_001622394_Nematostella_BCH RIV-------------TWFFTTFTAS------------------------

XP_396878_Apis_BCH KMM-------------TWWFTTFMAP------------------------

XP_001815509_Tribolium_BCH KMM-------------TWWFTTFMAP------------------------

NP_724599_Drosophila_mel_BCH KMM-------------TWWFTTFMAP------------------------

NP_724597_Drosophila_mel_BCH KMM-------------TWWFTTFMAP------------------------

XP_001867337_Culex_BCH KMM-------------TWWFTTFMAP------------------------

XP_001688494_Anopheles_BCH KMM-------------TWWFTTFMAP------------------------

XP_001660566_Aedes_BCH KMM-------------TWWFTTFMAP------------------------

EEB16863_Pediculus_BCH KMV-------------SWWFTTFMAP------------------------

ACO15636_Caligus_BCH KLT-------------CWWFSTFMAP------------------------

XP_002112464_Trichoplax_BCH KWS-------------FWSFCTFTAP------------------------

XP_423602_Gallus_BCH KVS-------------TWFFTTFTV-------------------------

XP_002197809_Taeniopygia_BCH KVS-------------AWFFTTFTV-------------------------

XP_001144527_Pan_BCH KVS-------------TWFFTTFSV-------------------------

NP_060156_Homo_BCH KVS-------------TWFFTTFSV-------------------------

XP_001500874_Equus_BCH KVS-------------TWFFTTFSV-------------------------

NP_001039478_Bos_BCH KVS-------------TWFFTTFSV-------------------------

XP_533021_Canis_BCH KVS-------------TWFFTTFSV-------------------------

XP_001365992_Monodelphis_BCH KVS-------------TWFFTTFSV-------------------------

NP_001013219_Rattus_BCH KVS-------------TWFFTTFSV-------------------------

NP_034399_Mus_BCH KVS-------------TWFFTTFSV-------------------------

XP_001104476_Macaca_BCH KVS-------------TWFFTTFSV-------------------------

NP_001004563_Danio_BCH KVS-------------TWFFTTFSV-------------------------

CAF93717_Tetraodon_BCH KVS-------------TWFFTTFSV-------------------------

ACO13754_Esox_BCH KVS-------------TWFFTTFSV-------------------------

NP_001088345_Xenopus_BCH KVS-------------TWFFTTFTV-------------------------

XP_001512063_Ornithorhynchus_B KVECAPYRPGCSGPGLCWLCGSPTVREAGWA-------------------

XP_001776374_Physcomitrella_BC KTT-------------VMALS-LLVEP-----------------------

XP_001759372_physcomitrella_BC KAT-------------VMALN-LLVEP-----------------------

EEF01185_Populus_BCH KTT-------------IFALQ-VFVDN-----------------------

EEE89642_Populus_BCH KAT-------------IFALQ-VFVDK-----------------------

EEF38953_Ricinus_BCH KAT-------------ILALQ-LLVDN-----------------------

NP_564960_Arabidopsis_BCH KAT-------------ILTMQ-FFVDN-----------------------

XP_002467159_Sorghum_BCH RTA-------------VLAMQ-MFVDG-----------------------

ACG47767_Zea_BCH RTA-------------VLAMQ-MFVDG-----------------------

NP_001064706_Oryza_BCH RTA-------------ILAMQ-MFVDG-----------------------

XP_002465844_Sorghum_BCH RTA-------------VMAMQ-LFVDG-----------------------

ACG45534_Zea_BCH RTA-------------VMAMQ-LFVDG-----------------------

ABK24296_Picea_BCH KAT-------------IFALQ-LLVDA-----------------------

EEE58332_Oryza_BCH RTA-------------ILALQ-LFVDG-----------------------

EEE91516_Populus_BCH RLV-------------FATLGRFFLSG-----------------------

EEF30886_Ricinus_BCH RLV-------------FATLGRFFLSG-----------------------

ACU17631_Glycine_BCH RLV-------------IATLGRIFLSG-----------------------

ABD28324_Medicago_BCH RLV-------------MATLGRFFLSG-----------------------

ABR17649_Picea_BCH RLL-------------LATLGRYFLSE-----------------------

XP_001754720_Physcomitrella_BC RLF-------------LGTLGRFFLSE-----------------------

NP_001047687_Oryza_BCH RLF-------------FATLGRFLFSS-----------------------

ACG26273_Zea_BCH RLF-------------FATFGRFLFSS-----------------------

ACG34784_Zea_BCH RIF-------------FATFGRFLFSS-----------------------

BAF15465_Oryza_BCH RLF-------------FSTLGRFLFSS-----------------------

EEF31637_Ricinus_BCH RLF-------------LATFGRFLFSG-----------------------

NP_195300_Arabidopsis_BCH RLF-------------LATCGRFLFSG-----------------------

ACJ85167_Medicago_BCH RLF-------------LATFGRFFFNA-----------------------

ACU14441_Glycine_BCH RLF-------------LATFGRFLFNA-----------------------

EEE75109_Populus_BCH KLF-------------LATFGRLHFGS-----------------------

EEE93960_Populus_BCH RLF-------------LATLGRFLFSG-----------------------

EEE81211_Populus_BCH RLF-------------LATFGRFLFSG-----------------------

EEF52804_Ricinus_BCH RLF-------------FATFGRLLFSG-----------------------

CAN69115_Vitis_BCH RLF-------------FATFGRFLFNG-----------------------

XP_001699293_Chlamydomonas_BCH WGA-------------LATLG-PLLSA-----------------------

XP_002168194_Cnidarian_NF1 IQYMELN---------ERVMKPGIAKN-----------------------

XP_001744997_Coano_NF1 -AFLGLA---------KRLNRYVLGSK-----------------------

AAB59558_human_NF1 REYTKYH---------ERLLTGLKGS------------------------

XP_692937.3_Danio_NF1 REYTKYH---------ERLLTGLKGS------------------------

XP_002587546_lancelet_NF1 REYTKYH---------ERILANLKGN------------------------

AAB58976_Drosophila_NF1 REYTKFH---------DRILAPLKGN------------------------

XP_001196417_Echinoderm_NF1 KEYIKRN---------ERVFVLLKGT------------------------

XP_002576216_tremtoda_NF1 REYTKIH---------DRFFSPIKGS------------------------

XP_002115170_placozoa_NF1 RNYVKYH---------ERVFQRVKGN------------------------

XP_002121778_ciona_NF1 RDYVSYN---------EGSFNQVEGH------------------------

NP_013796_Saccharomyces_CRALTR STA-------------FRLFKPFLDP------------------------

NP_012832_Saccharomyces_CRALTR STM-------------FKMVKPFLDP------------------------

NP_195629_Arabidopsis_CRALTRIO RLL-------------WNTVKSFLDP------------------------

NP_036561_Homo_CRALTRIO PVA-------------YNLIKPFLSE------------------------

NP_777635_Homo_CRALTRIO PVG-------------YNLMKPFLSE------------------------

NP_001091144_Xenopus_CRALTRIO PVA-------------YNLIKHFLSE------------------------

NP_001093463_Danio_CRALTRIO PVA-------------YNLVKHFLSE------------------------

NP_777637_Homo_CRALTRIO PVA-------------FNLVKSFMSE------------------------

NP_612042.3_Drosophila_CRALTRI SVA-------------FNIVKKFLDE------------------------

NP_001087870_Xenopus_CRALTRIO PVL-------------WTLVSPFIDE------------------------

NP_001085706_Xenopus_CRALTRIO PVL-------------WTLVSPFIDE------------------------

NP_001137470_Homo_CRALTRIO PVL-------------WTLVSPFIDD------------------------

NP_957392_Danio_CRALTRIO PVL-------------WTLVSPFIDE------------------------

NP_055507_Homo_CRALTRIO PVL-------------WTLISPFINE------------------------

NP_001040875_Caenorhabditis_CR PVL-------------WTLISPFIDE------------------------

NP_609028_Drosophila_CRALTRIO PIA-------------WTIVSAFIDE------------------------

NP_506408_Caenorhabditis_CRALT SAI-------------WTIAKPLLPE------------------------

NP_510554_Caenorhabditis_CRALT TAL-------------YVVVRPLLPE------------------------

NP_495168_Caenorhabditis_CRALT TLL-------------WKAIGPLLPE------------------------

NP_505746_Caenorhabditis_CRALT NVL-------------WSALSPFIPE------------------------

CAB78040_Arabidopsis_CRALTRIO LAF-------------YRIISPFMSQ------------------------

NP_177360_Arabidopsis_CRALTRIO IPY-------------YKTFGSIITS------------------------

NP_013484_Saccharomyces_CRALTR NPI-------------WNIIKNWLDP------------------------

XP_643391_Dict_CRALTRIO MGI-------------WHIIKHWLDP------------------------

NP_728794_Drosophila_CRALTRIO STI-------------WPAIRVLLDD------------------------

NP_014168_Saccharomyces_CRALTR WTF-------------LKLIHPFIDP------------------------

NP_014135_Saccharomyces_CRALTR WAF-------------LKMMYPFLDP------------------------

XP_629043_dict_CRALTRIO NVF-------------WTTISPFINK------------------------

NP_195382_Arabidopsis_CRALTRIO QAV-------------YRAAKYFLDP------------------------

BAH19871_Arabidopsis_CRALTRIO ESF-------------YKMVKPFLEP------------------------

NP_001088147_Xenopus_CRALTRIO NAA-------------FKIVKSWLGP------------------------

NP_001088926_Xenopus_CRALTRIO NAA-------------FKIVKSWLGP------------------------

NP_689794_Homo_CRALTRIO NAA-------------FKIVKTWLGP------------------------

NP_001007294_Danio_CRALTRIO NAA-------------WKIVKTWLGP------------------------

NP_729113_Drosophila_CRALTRIO DAA-------------FKLVKTFLPP------------------------

NP_000317_Homo_CRALTRIO TTT-------------YNVVKPFLKS------------------------

NP_001080386_Xenopus_CRALTRIO TTT-------------YNVVKPFLKS------------------------

NP_956999_Danio_CRALTRIO TTT-------------YNVVKPFMKS------------------------

NP_775790_Homo_CRALTRIO HAL-------------YTLIKPFLKD------------------------

NP_001088883_Xenopus_CRALTRIO HAL-------------YTIIKPFLKD------------------------

NP_001010852_Homo_CRALTRIO HAL-------------YTVIRPFLKE------------------------

NP_001020716_Danio_CRALTRIO HAL-------------YTVIRPFLKD------------------------

NP_001034288_Homo_CRALTRIO KGI-------------FAIIKPFLKE------------------------

NP_997755_Danio_CRALTRIO KGI-------------FAIIKPFLKE------------------------

NP_000361_Homo_CRALTRIO HAV-------------FSMIKPFLTE------------------------

NP_724195_Drosophila_CRALTRIO NAA-------------FKIFKPFLNA------------------------

NP_609120_Drosophila_CRALTRIO NML-------------FAVFKPFIRE------------------------

NP_001079459_Xenopus_CRALTRIO RAP-------------FAVLRLFVRE------------------------

NP_001081203_Xenopus_CRALTRIO RAP-------------FAVLRLFVRE------------------------

NP_572576_Drosophila_CRALTRIO KAP-------------FKILRLFVRE------------------------

NP_002824_Homo_CRALTRIO RVP-------------YSIISLLLKD------------------------

NP_724813_Drosophila_CRALTRIO VAL-------------LNLAKSLMPS------------------------

NP_610507_Drosophila_CRALTRIO QFV-------------LGLVRNVM--------------------------

NP_001021496_caenorhabditis_Rh EKQKAQMSLGT-----WDFEVEMISFE-----------------------

XP_001891671_brugia_RhoGEF EKHKTSVSSGK-----YKFDIQMISVE-----------------------

NP_009049_Homo_RhoGEF QKQRTNFGSSK-----FEFETNMVSLE-----------------------

NP_001097996_danio_RhoGEF QKQRTNFGSSK-----FEFETTMVSLE-----------------------

NP_001019831_Homo_RhoGEF QKQKTNFGSSK-----FIFETSMVSVE-----------------------

XP_002598613_branchiostoma_Rho QKHRTSLGSSK-----FSFETSMIALD-----------------------

NP_651960_drosophila_RhoGEF QKQRASISSHK-----YKFETTTVSIE-----------------------

XP_002578747_schistosoma_RhoGE EKHKAQLASGK-----FSFEIQLVSVE-----------------------

XP_001635815_nematostella_RhoG ERHKTNLKTSK-----LSFETTLTSLD-----------------------

NP_835224.3_Homo_RhoGEF DKKVTHFCFWKEKDR-LGFEVILVSAN-----------------------

NP_001085886_xenopus_RhoGEF DKKVTHFCFWKEKDR-LGFEVILVSGN-----------------------

NP_878301_danio_RhoGEF DKKVTHFCFWKEKDR-LGFEVILVSAN-----------------------

NP_001106203_Homo_RhoGEF QRTLSDIAFKFNRDD-FKMKVPVIMLSSV---------------------

NP_055893_Homo_RhoGEF QRTFTDIGIKYYRNE-FKTKVPIIMVNSV---------------------

NP_001093325_Homo_RhoGEF QRTFTDIGFWFSQED-FMLKLPVVMLSSV---------------------

NP_001033984_Drosophila_CRALTR DTI-------------FNMIKPMMS-------------------------

NP_651174_Drosophila_CRALTRIO QTI-------------FNFVTKFLPS------------------------

NP_496771_Caenorhabditis_CRALT NVL-------------HQACSPFLP-------------------------

XP_002110939_trichoplax_RhoGEF KQID-----------ITDLTEELQGILP----------------------

XP_640612_Dictyostelium_BCH KFT-------------LGIFKHFLSS------------------------

XP_001420825_Ostreococcus_luc_ KAA-------------IWTMDMLQLES-----------------------

NP_491993_Caenorhabditis_CRALT RIA-------------YASITAILQDP-----------------------

EFA82125_poly_NF1 KKF-------------MKRVSKLIG-------------------------

XP_638573_Dictyostelium_BCH KTT-------------LFISKSILGDK-----------------------

NP_871646_Caenorhabditis_CRALT NAS-------------WKVIGSWLESS-----------------------

XP_645940_Dictyostelium_BCH KIL-------------LLAMSPFLS-------------------------

NP_171669_Arabidopsis_CRALTRIO MTA-------------WKVIYPFIDAN-----------------------

XP_646383_dict_CRALTRIO FA--------------WKIISPFLN-------------------------

XP_001347356_Plasmodium_fal_BC KTL-------------LTIVTPFISP------------------------

XP_002140404_Cryptosporidium_B RAF-------------MSVISSYMSP------------------------

NP_569990_Drosophila_CRALTRIO DKL-------------ISMMSPFLR-------------------------

NP_199584_Arabidopsis_CRALTRIO SAC-------------WKVVKPLLQER-----------------------

NP_508618_Caenorhabditis_CRALT SAV-------------YAMVSPVLSS------------------------

XP_001702344_Chlamydomonas_BCH RAL-------------LALAQPFVSR------------------------

XP_646478_dict_CRALTRIO EKL-------------FSIFTVFTSER-----------------------

NP_012390_Saccharomyces_CRALTR GWV-------------YDLIKKFVDET-----------------------

XP_642079_dict_CRALTRIO KPL-------------LSLAKTFMKN------------------------

XP_645456_Dictyostelium_BCH RAV-------------IYFMSAFTSR------------------------

XP_001548197_yeast_NF1 RKC-----------------------------------------------

XP_571310_yeast_NF1 RKR------------LRHLISELLTIS-----------------------

NP_593281_Schizosaccharomyces_ KVA-------------IRTLLSIASP------------------------

NP_001024391_Caenorhabditis_CR HVP-------------YKIAKAMMPTG-----------------------

NP_872173_Caenorhabditis_CRALT SVM-------------WQVTKRLVDP------------------------

NP_497717_Caenorhabditis_CRALT GLL-------------WKVARVFLSE------------------------

XP_001173154_Pan_BCH --SSKFSQKIRYVFNLAE------------LAELVPME-YVGIPECIKQ

XP_544705_Canis_BCH --SSKFSQKIRYVFNLAE------------LAELVPME-YVGIPECIKQ

XP_001374074_Monodelphis_BCH --SSKFSQKIKYVFNLAE------------LAELVPME-YVGIPECIKQ

NP_001008239_Mus_BCH --SSKFSQKIRYVFNLAE------------LAELVPME-YVGIPECIKQ

EDL84204_Rattus_BCH --SSKFSQKIRYVFNLAE------------LAELVPME-YVGIPECIKQ

XP_001100508_Macaca_BCH --SSKFSQKIRYVFNLAE------------LAELVPME-YVGIPECIKQ

NP_004321_Homo_BCH --SSKFSQKIRYVFNLAE------------LAELVPME-YVGIPECIKQ

XP_001510218_Ornithorhynchus_B --SSKFSQKIRYVFNLAE------------LAELVPME-YVGIPECIKQ

XP_413765_Gallus_BCH --SSKFSQKIRYVFTLAE------------LAELIPME-YVGIPECIKQ

XP_002195589_Taeniopygia_BCH --SSKFSQKIRYVFTLAE------------LAELIPME-YVGIPECIKQ

NP_001088131_Xenopus_BCH --SSKFCQKIKYVFSLVE------------LAELIPME-YVGIPECIKE

CAF98314_Tetraodon_BCH SHSSKFSQKIQFVYSLAD------------LAELVPME-YVSIPDCIKQ

NP_957512_Danio_BCH --SSKFSQKIKFVFSLTD------------LAELVPME-YVSIPDCIKQ

XP_001366186_Monodelphis_BCH --SSKFSSKIQYVSTLSE------------LSELIPME-CVHIPESIIK

XP_001916938_Equus_BCH --SSKFSSKIKYVSSLSE------------LSGLIPMD-CIHIPESIIK

NP_056040_Homo_BCH --SSKFSSKIKYVNSLSE------------LSGLIPMD-CIHIPESIIK

XP_001146885_Pan_BCH --SSKFSSKIKYVNSLSE------------LSGLIPMD-CIHIPESIIK

NP_001126916_Pongo_abe_BCH --SSKFSSKIKYVNSLSE------------LSGLIPMD-CIHIPESIIK

NP_001098852_Bos_BCH --SSKFSSKIKYVSSLAE------------LSGLIPMD-CIHIPESIIK

NP_851993_Mus_BCH --SSKFSSKIKYVTSLSE------------LSGLIPMD-CIHIPESIIK

Q5BJR4_Rattus_BCH --SSKFSSKIKYVSSLSE------------LSGLIPMD-CIHIPESIIK

XP_002187337_Taeniopygia_BCH --SSKFSSKIQYVNTLAE------------LHEMIPME-YVHIPDSIVK

XP_001231886_Gallus_BCH --SSKFSSKIQYVNTLAE------------LREMIPME-YVHIPDSIVK

XP_850604_Canis_BCH --SSKFSSKIKYVNSLSE------------LSGLIPMD-CIHIPESIIK

XP_001515884_Ornithorhynchus_B --SSKFSSKIQYVSSLSE------------LSELIPME-YVPIPESIIK

CAG08427_Tetraodon_BCH --STKFSSKIKYVNSLQE------------LGQIIPLE-YVNIPASIVR

NP_001104624_Danio_BCH --SSKFSSKIKYVNSLAE------------LEELIPME-YVHIPECIVR

CAF97194_Tetraodon_BCH --SAKFSSKIKYVSSLDE------------LEKLIPME-SIQIPECIIR

NP_001133314_Salmo_BCH --STKFSNKIKYVNSLAE------------LQELIPME-HVHIPECIIS

XP_001374042_Monodelphis_BCH --SVKFISKIQYVHSLEE------------LEQLIPME-HVQIPDCIQQ

XP_001511480_Ornithorhynchus_B --SVKFINKIQYVHSLEE------------LEQIIPME-HVQIPDCVQL

XP_542168_Canis_BCH --SVKFINKIQYVHSLED------------LEQLIPME-HVQIPDCVLQ

NP_001125042_Pongo_abe_BCH --SVKFINKIQYVHSLED------------LEQLIPME-HVQIPDCVLQ

NP_001075898_Bos_BCH --SVKFINKIQYVHSLED------------LEQLIPME-HVQIPDCVLQ

NP_848777_Mus_BCH --SVKFISKIQYVHSLEE------------LERLIPME-HVQLPDCVLQ

NP_001035280_Rattus_BCH --SVKFISKIQYVHSLEE------------LEQLIPME-HVQLPACVLQ

XP_524457_Pan_BCH --SVKFINKIQYVHSLED------------LEQLIPME-HVQIPDCVLQ

BAC03859_Homo_BCH --SVKFINKIQYVHSLED------------LEQLIPME-HVQIPDCVLQ

XP_001493463_Equus_BCH --SVKFINKIQYVHSLEG------------LEQLIPME-HVQIPDCVLQ

XP_418214_Gallus_BCH --SVKFINKIQYVHSLEE------------LEQLIPME-HVQIPDCVSQ

XP_002195707_Taeniopygia_BCH --SVKFINKIQYVHSLEE------------LEQLIPME-HVQIPDCVLQ

CAF91924_Tetraodon_BCH --SVKFMDKIRHVHTLEE------------LSRFIPME-HVQIPECVQQ

XP_001343600_Danio_BCH --NVKFMDKIRYVQSLQE------------LAQIVPME-HVQIPECVLQ

CAG08009_Tetraodon_BCH --SMKFLNKIQYVHSLDE------------LAEMVPME-HVHVPDCVLQ

CAN87813_Danio_BCH --SVKFMNKIRYVHSLEE------------LEKFVPMD-HIHIPECILQ

XP_001170701_Pan_BCH --SSKFTRKIRFLDSLGE------------LAQLISLD-QVHIPEAVRQ

NP_612122_Homo_BCH --SSKFTRKIRFLDSLGE------------LAQLISLD-QVHIPEAVRQ

XP_001106978_Macaca_BCH --SSKFTRKIRFLDSLGE------------LAQLVSLD-QVHIPEAVRQ

XP_001491310_Equus_BCH --SSKFTRKIRFLNSLGE------------LAQLISLD-QVHIPEAVRQ

XP_001928046_Sus_BCH --SSKFTRKIRFLNSLGE------------LAQLISMD-QVHIPEAVRQ

NP_001073089_Bos_BCH --SSKFTRKIRFLNSLGE------------LAQLISMD-QVHIPEVVRQ

EDL38792_Mus_BCH --SSKFTRKIRFLDSLGE------------LAQLISLE-QVHIPEVVRQ

NP_001121659_Rattus_BCH --SSKFTRKIRFLDSLGE------------LAQLISLE-QVHIPEAVRQ

XP_540308_Canis_BCH --SSKFTRKIRFLNSLGE------------LAQLISLD-QVHIPEAVRQ

XP_001371249_Monodelphis_BCH --SSKFSRKVRFLDSLEE------------LAQLVSLE-HIHIPDAVRQ

NP_001086594_Xenopus_BCH --SSKFWKKVKFISSLED------------LSMVVSME-QIHIPDCIRQ

NP_001121866_Danio_BCH --SEKFSRKMRFIHSLQE------------LAEFVPVE-QLQIPDCIRE

XP_002127893_Ciona_BCH --SSKFSKKLKLVSTLHR------------LADVVTLD-NVVIPDMVQQ

EEC09901_Ixodes_BCH --SSKFSRKLRFVNSIEE------------LSGLVPLD-HVSIPDKVKQ

XP_001947520_Acyrthosiphon_BCH --SSKFGNKIHFINSLDE------------LYDCVPVE-RASIPDRVKQ

EEB18677_Pediculus_BCH --SSKFSKKLFFINSLSE------------LYKIIPIE-ESCIPDRVKK

XP_315572_Anopheles_BCH --SSKFWRKLVYVTSLEE------------LYKLVPVE-KAAVPDKVKN

XP_001648558_Aedes_BCH --SSKFWRKLVYVKTLED------------LYKLVPVE-RAAVPDKVKN

ABY20545_Drosophila_mel_BCH --STKFWRKLVYVKSLEE------------LGMHVVVE-KAAIPEKVKQ

XP_974968_Tribolium_BCH --SSKFYRKISYVSSLKE------------LMVRVPLE-AAAIPDKVKA

XP_783795_Strongylocentrotus_B --SAKFSNKLKFVKSLVE------------LKSLVSME-YVYIPEEVKR

XP_001626140_Nematostella_BCH --STNFWCKLQFVKSLSD------------LSELIPIE-YIYIPDEVIR

XP_002126187_Ciona_BCH --SRKAFKKLRHIKGMDE------------LKKYIPID-FLYIPNSREK

XP_508398_Pan_BCH LISFKFGQKIFYVNYLSE------------LSEHVKLE-QLGIPRQVLK

CAM14521_Mus_BCH LISFKFGRKIFYVNYLSE------------LSEHVKLE-QLGIPRQVLK

NP_001124902_Pongo_abe_BCH LISFKFGQKIFYVNYLSE------------LSEHVKLE-QLGIPRQVLK

XP_001101907_Macaca_BCH LISFKFGQKIFYVNYLSE------------LSEHVKLE-QLGIPRQVLK

BAG60756_Homo_BCH LISFKFGQKIFYVNYLSE------------LSEHVKLE-QLGIPRQVLK

NP_001101217_Rattus_BCH LISFKFGRKIFYVNYLSE------------LSEHVKLE-QLGIPRQVLK

XP_001490021_Equus_BCH IISFKFGQKIFYVNYLSE------------LSEHVKLE-QLGIPRQVLK

XP_871999_Bos_BCH IISFKFGQKIFYVNYLSE------------LSEHVKLE-QLGIPRQVLK

XP_001369707_Monodelphis_BCH LISFQFGRKIFYVNYLSE------------LCEHVKLE-HLGIPRQVLK

XP_426422_Gallus_BCH LISFKFGRKIFYVNFLSE------------LEEYVKLE-QLGIPSQVLK

XP_002198819_Taeniopygia_BCH LISFKFGRKIFYVNYLSE------------LEEYVKLE-QLGIPSQVLK

NP_001086509_Xenopus_BCH IISFKFGRKIFYANYLSD------------LEEHTKLE-QLGIPKQVLK

NP_001017781_Danio_BCH IISFKFGRKINYINYLSE------------LEEIVKCD-QLVIPNRVRE

EDL04450_Mus_BCH LI-------------------------------HLQCD-QLLIPPEVVR

NP_001004242_Rattus_BCH LISHKFGKKVTYCSNLRE------------LREHLQCD-QLLIPPEVVR

CAQ06715_Homo_BCH LISHKFGKKVIYFNYLSE------------LHEHLKYD-QLVIPPEVLR

XP_416463_Gallus_BCH LISHKFGKKVTYLNYLSD------------LREHLKYD-QLNIPQEVVR

NP_001085153_Xenopus_BCH VISHKFGKKVLYMKDLND------------LHEHLKFN-RLIIPPEVLQ

EEB11750_Pediculus_BCH VISAKFGRKVMYVNSLQE------------LHNLVELPYQLSIPQPVIE

XP_001950301_Acyrthosiphon_BCH VISAKFGRKIMYVNHLQE------------LKLFMDLD-QLIIPAPVLD

XP_001604462_Nasonia_BCH AISAKFGRKMMYVNYLDE------------LAQYINLD-QLIIPQQVIE

XP_624226_Apis_BCH AISAKFGRKMMYVNYLEE------------LAQYINLD-QLIIPPQVIE

XP_002121397_Ciona_BCH LISVKFGRKMSYVNYLHE------------IADVVHLD-QLPIPQDVLD

XP_970580_Tribolium_BCH LISAKFGKKINYINTLKE------------LNEHISLE-KLDIPEEVIE

XP_002160831_Hydra_BCH FISKKFGKKINNISKLDE------------LTPHLRID-QIDIPEEVKQ

NP_001022390_Caenorhabditis_el FISSKFENKFHYVMCIDE------------LENALSVA-RLNLPSPIRD

XP_001893352_Brugia_BCH FISIKFEQKIHYVNYLHE------------LDSILRVE-QLSLPQPIKE

XP_002117739_Trichoplax_BCH IISRKFGRKVQYINRLEE------------LKEFTHYD-QLDIPSEVDE

XP_002572184_Schistosoma_BCH FISSKMSNKVVYAETLSE------------LEETLFVD-QLPIPQRVLN

EEC10256_Ixodes_BCH YIRNVLAFSLETVYTLRT------------LSTLCRQTVLWTRVKTAGR

XP_001746114_Monosiga_BCH FIRSARTRPHIFDVCACV------------SRSNLAPCVVPANPSSKFG

XP_001835505_Coprinopsis_BCH IISPKFFRKIEYVSTLSE------------LAHYVPLT-QIDIPPAVYQ

XP_001876797_Laccaria_BCH IISPKFFRKLSYIATLSE------------LAQRVPLT-QIDIPPTVYQ

XP_760803_Ustilago_BCH IVSPKFSKKVSQLDTLSK------------LAECVPLT-QIDIPPEVLQ

XP_001843366_Culex_BCH --SEKFKSKLIYTSSLDE------------LKQSLGLN-TLKVPDTVRE

XP_001659702_Aedes_BCH --SEKFKSKLIYTSSLDE------------LKQSLGLN-TLKVPDTVRE

NP_648552_Drosophila_mel_BCH --SDKFRKKLVYISSLDE------------LRQALGLN-KLKLPDNICD

XP_001182784_Strongylocentrotu ----SIKSKVHSTGAVHY------------LYKTIH-PDQLDIPPFVIE

XP_001622394_Nematostella_BCH ----SVKEKVHFLSGVQY------------LYDWIN-PDQLDIPAYVLE

XP_396878_Apis_BCH ----AIKQKVHNLPGVEY------------LYEVMS-PEQLEIPAYITE

XP_001815509_Tribolium_BCH ----AIKQKVHSLPGVEY------------LYAVMS-PDQLEIPAFITE

NP_724599_Drosophila_mel_BCH ----AIKAKVHSLPGVEH------------LYSAIT-KDQLEIPAYITE

NP_724597_Drosophila_mel_BCH ----AIKAKVHSLPGVEH------------LYSAIT-KDQLEIPAYITE

XP_001867337_Culex_BCH ----AIKTKVHSLPGVEH------------LYSAIA-KDQLEIPAYITE

XP_001688494_Anopheles_BCH ----AIKTKVHSLPGVEH------------LYSAIA-KDQLEIPAYITE

XP_001660566_Aedes_BCH ----AIKTKVHSLPGVEH------------LYSAIA-KDQLEIPAYITE

EEB16863_Pediculus_BCH ----AIKEKVFNIDGIEY------------LYSIII-PNQLEIPAYITE

ACO15636_Caligus_BCH ----AIKNKILNIHALSE------------LTPHLN-TKELNLPMFITE

XP_002112464_Trichoplax_BCH ----ELKSKLQYIDDLKD------------LLKFIP-RDQFSIPQYLFD

XP_423602_Gallus_BCH -SG--LKDKIHYVESLQQ------------LFTAIP-PEQIDLPPFVLE

XP_002197809_Taeniopygia_BCH -SG--LKDKIHYVESLQQ------------LFTAIP-PEQIDLPPFVLE

XP_001144527_Pan_BCH -SG--LKDKIHHVDSLHQ------------LFSAIS-PEQIDFPPFVLE

NP_060156_Homo_BCH -SG--LKDKIHHVDSLHQ------------LFSAIS-PEQIDFPPFVLE

XP_001500874_Equus_BCH -SG--LKDKIHHVDSLHQ------------LFSAIS-PEQIDFPPFVLE

NP_001039478_Bos_BCH -SG--LKDKIHHVDSLHQ------------LFSAIS-PEQIDFPPFVLE

XP_533021_Canis_BCH -SG--LKDKIHHVDSLHQ------------LFSAIS-PEQIDFPPFVLE

XP_001365992_Monodelphis_BCH -SG--LKDKIHHVDSLHQ------------LFAAIS-PEQIDFPPFVLE

NP_001013219_Rattus_BCH -SG--LKDKIHHVDSLQQ------------LFSAIS-PEQIDFPPFVLE

NP_034399_Mus_BCH -SG--LKDKIHHVDSLQQ------------LFSAIS-PEQIDFPPFVLE

XP_001104476_Macaca_BCH -SG--LKDKIHHVDSLHQ------------LFSAIS-PEQIDFPPFVLE

NP_001004563_Danio_BCH -SG--LKDKVHHIENLQQ------------LFTCVL-PEQIDIPPFVLE

CAF93717_Tetraodon_BCH -SG--LKEKVRYLDSLHQ------------LFTCIR-PEQIDIPPFVLE

ACO13754_Esox_BCH -SG--LKEKVHHIENLRQ------------LFSCIL-PEQIDIPPFVLD

NP_001088345_Xenopus_BCH -SG--LKDKVHQVESLHQ------------LFTAIP-PEQIEIPPFVLD

XP_001512063_Ornithorhynchus_B -SGPLLRLRRDLVEEEEEEEGGEGEPESVLSFSTLRYPHKATWAAWWQD

XP_001776374_Physcomitrella_BC ----EVWKKVVYVDKLAD-----------LFRYVPRE--QLTIPDFVFQ

XP_001759372_physcomitrella_BC ----EVWKKVVYVEKLSD-----------LFRYVPRE--QLTIPDFVFQ

EEF01185_Populus_BCH ----VTWKKVVYVDRLLQ-----------LFRYVPRE--QLTIPDFVFQ

EEE89642_Populus_BCH ----VTWKKVVYVDRLVQ-----------LFRYVPRE--QLTIPDFVFQ

EEF38953_Ricinus_BCH ----VTWKKVVYVDRLLQ-----------LFRHVPRE--QLTIPDFVFQ

NP_564960_Arabidopsis_BCH ----VVWKKVVYADRLLQ-----------LFKYVPRE--QLTIPDFVFQ

XP_002467159_Sorghum_BCH ----EVWKKVVYVDRLVQ-----------LFRYVPRE--QLTIPDFVFQ

ACG47767_Zea_BCH ----EVWKKVVYVDRLVQ-----------LFRYVPRE--QLTIPDFVFQ

NP_001064706_Oryza_BCH ----EVWKKVVYVDRLVH-----------LFRYVPRE--QLTIPDFVFQ

XP_002465844_Sorghum_BCH ----EVWKKVVYVDRLVQ-----------LFRYVPRE--QLTIPDFVFQ

ACG45534_Zea_BCH ----EVWKKVVYVDRLVQ-----------LFRYVPRE--QLTIPDFVFQ

ABK24296_Picea_BCH ----EVWRKVVYVERLLQ-----------LFRYVPRE--QLTIPDFVFQ

EEE58332_Oryza_BCH ----EVWKKVIYVDRLVQ-----------LFRYVPRE--QLTIPDFVFQ

EEE91516_Populus_BCH ----GLYWKIKYVSRLQY-----------LWEDIKKG--EIEIPEFVQN

EEF30886_Ricinus_BCH ----GLYWKIKYVSRLQY-----------LWEDIKKG--EVEIPEFVQS

ACU17631_Glycine_BCH ----GLYWKIKYVSRLQY-----------LWDDIKKG--EIEIPDFVKS

ABD28324_Medicago_BCH ----GLYWKIKYVSRLQY-----------LWDDIKKG--EIEIPEFVQK

ABR17649_Picea_BCH ----GLYWKLKYINRLEF-----------LWSDIKKG--QIEIPEFVCE

XP_001754720_Physcomitrella_BC ----GFYSKLVYISRLEF-----------LTEHVRES--QVEIPEFVID

NP_001047687_Oryza_BCH ----GLYEKLRYMSRLEY-----------LWEHVSKG--EMEVPECARR

ACG26273_Zea_BCH ----GLYEKLRYMSRLEY-----------LWAHIDKG--ELEVPEFARR

ACG34784_Zea_BCH ----GLYEKLRYMSRLEY-----------VWAHIDKE--QLEVPECVRE

BAF15465_Oryza_BCH ----GLYGKLRYVSRLEY-----------LWAHVRKG--ELDVPEAVRR

EEF31637_Ricinus_BCH ----GLYGKLRYISRIDY-----------LWDHVRRN--EVEIPEFVHD

NP_195300_Arabidopsis_BCH ----GLYGKLRYISRVDY-----------LWEHVRRN--EIEMPEFVYD

ACJ85167_Medicago_BCH ----GLYRKLRYISRIGY-----------LWENVRRT--EVEIPEFVYD

ACU14441_Glycine_BCH ----GLYGKLRYVSRVDY-----------LWESVRRN--EVEIPEFVFD

EEE75109_Populus_BCH ----RLYGKLRYINRIDY-----------LWDPIRRN--EVKIPEFVCD

EEE93960_Populus_BCH ----GLYSKLRYVTRLEF-----------LWDHVRRS--EIEIPEFAYD

EEE81211_Populus_BCH ----GLYSKLKYVTRMEF-----------LWDHVRRN--EIGIPEFAYD

EEF52804_Ricinus_BCH ----GLYGKLRYVSRLGF-----------LWDHVRRN--EFEIPQFAYD

CAN69115_Vitis_BCH ----GLYQKLQYVNRLEF-----------LWSHVRRN--GLEIPEFVLD

XP_001699293_Chlamydomonas_BCH ----DFWRKVEWVSRVEF-----------LWDHIPKKQLLSALPAYVAE

XP_002168194_Cnidarian_NF1 ------IQKIVIIDKLSK------------LYEYIEN---VKLPSSTLT

XP_001744997_Coano_NF1 ------FHKKVAFVNLPE------------LQAQIPK---LQLPASTLR

AAB59558_human_NF1 -------KRLVFIDCPGK------------LAEHIEHE-QQKLPAATLA

XP_692937.3_Danio_NF1 -------KKLLFIDSPAR------------LAEHVEPE-QQKLPAATLA

XP_002587546_lancelet_NF1 -------RKLIFIDSANK------------FSDYIHPD-QQHLPGATTA

AAB58976_Drosophila_NF1 -------RKLLFLESPNK------------LTDFIDAE-QQKLPGATLS

XP_001196417_Echinoderm_NF1 -------RRIIFIESVAK------------LGEYIEPD-QQRLHKATLG

XP_002576216_tremtoda_NF1 -------RKLIFIDHPSR------------LNEYIEPD-QQRLPAGTLV

XP_002115170_placozoa_NF1 -------EKIIVIDELTK------------IYEYIEEN-ELKLPSATTS

XP_002121778_ciona_NF1 -------RRLVGVD-LLE------------LKKFIELD-HQKVPSATRG

NP_013796_Saccharomyces_CRALTR ----VTVSKIFILG---SS----------YQKELLKQIPAENLPVKFGG

NP_012832_Saccharomyces_CRALTR ----VTVSKIFILG---SS----------YKKELLKQIPIENLPVKYGG

NP_195629_Arabidopsis_CRALTRIO ----KTTAKIHVLG---NK----------YQTKLLEIIDANELPEFLGG

NP_036561_Homo_CRALTRIO ----DTRKKIMVLG---AN----------WKEVLLKHISPDQVPVEYGG

NP_777635_Homo_CRALTRIO ----DTRRKIIVLG---NN----------WKEGLLKLISPEELPAQFGG

NP_001091144_Xenopus_CRALTRIO ----DTRKKIMVLG---DN----------WQEVLKKYIAPEELPQYYGG

NP_001093463_Danio_CRALTRIO ----DTRRKVIVLG---SN----------WQEVLQKYIDPEELPAYYGG

NP_777637_Homo_CRALTRIO ----ETRRKIVILG---DN----------WKQELTKFISPDQLPVEFGG

NP_612042.3_Drosophila_CRALTRI ----NTTSKIVIYKSGVDR----------WQEQLFSHVNRKAFPKAWGG

NP_001087870_Xenopus_CRALTRIO ----NTRKKFLIYAGNDYQ----------GPGGLIDYIDKEVIPDFLGG

NP_001085706_Xenopus_CRALTRIO ----NTRKKFLIYAGNDYQ----------GPGGLIDYIDKEVIPDFLGG

NP_001137470_Homo_CRALTRIO ----NTRRKFLIYAGNDYQ----------GPGGLLDYIDKEIIPDFLSG

NP_957392_Danio_CRALTRIO ----NTRKKFLIYAGNDYQ----------GPGGLVDYINKDCIPDFLGG

NP_055507_Homo_CRALTRIO ----NTRRKFLIYSGSNYQ----------GPGGLVDYLDREVIPDFLGG

NP_001040875_Caenorhabditis_CR ----KTRKKFMVSGGSGGD----------LKEELRKHIEEKFIPDFLGG

NP_609028_Drosophila_CRALTRIO ----HTRSKFLFYGPDCAH----------MKDGLAQYLDEEIVPDFLGG

NP_506408_Caenorhabditis_CRALT ----RTRNKCNIL---NSE----------WRVEVLKMAEGSCLPSYWND

NP_510554_Caenorhabditis_CRALT ----KTREKVRLIG--ETN----------WRDDVLQYAIHSSLPSIWNN

NP_495168_Caenorhabditis_CRALT ----RTRNKVRICSG-NSD----------WKTSVQKHAHIDNIPKHWGG

NP_505746_Caenorhabditis_CRALT ----QS--KQRIVFA-GSN----------WKEELLDIVDKECLPERYGG

CAB78040_Arabidopsis_CRALTRIO -----RSKSKLVFAG-PSR----------SAETLLKYISPEHVPVQYGG

NP_177360_Arabidopsis_CRALTRIO ----PRTRSKMVLAG-PSK----------SADTIFKYIAPEQVPVKYGG

NP_013484_Saccharomyces_CRALTR ----VVASKIVFTKN---------------IDELHKFIQPQYIPRYLGG

XP_643391_Dict_CRALTRIO ----NTASKVSFVK----------------TKQLVDYIPKDQLESSYGG

NP_728794_Drosophila_CRALTRIO ----NTAKKVKFVAD---------------EAELCQYLIPDILPTDM--

NP_014168_Saccharomyces_CRALTR ----LTREKLVFDEP------------------FVKYVPKNELDSLYGG

NP_014135_Saccharomyces_CRALTR ----ATKAKAIFDEP------------------FENHIEPSQLDALYNG

XP_629043_dict_CRALTRIO ----VTYKKIVFANGE-KQ----------KIKVFSQFFEPNDLEKEFTG

NP_195382_Arabidopsis_CRALTRIO ----RTAEKVKFVYPKDKA----------SDELMTTHFDVENLPKEFGG

BAH19871_Arabidopsis_CRALTRIO ----KTSNKVKFVYSDDNL----------SNKLLEDLFDMEQLEVAFGG

NP_001088147_Xenopus_CRALTRIO ----EAINLLKFVNK----------------SQVQDYISAEYLPPLMGG

NP_001088926_Xenopus_CRALTRIO ----EAINVLKFVNK----------------NQVQDYISIEYLPPHMGG

NP_689794_Homo_CRALTRIO ----EAVSLLKFTSK----------------NEVQDYVSVEYLPPHMGG

NP_001007294_Danio_CRALTRIO ----DAISKLKFVSK----------------SDIQTFVGPEHLPPYMGG

NP_729113_Drosophila_CRALTRIO ----EALKILKVTTK----------------KDIDQYVDKDNCLKIWGG

NP_000317_Homo_CRALTRIO ----KLLERVFVHGD--------------DLSGFYQEIDENILPSDFGG

NP_001080386_Xenopus_CRALTRIO ----KLLERVFVHGD--------------GLEGFFKEIDADILPADFGG

NP_956999_Danio_CRALTRIO ----KLLERVFVHGD--------------ELDGYLRDFGAEILPPDFDG

NP_775790_Homo_CRALTRIO ----KTRKRIFLHGN--------------NLNSLHQLIHPEFLPSEFGG

NP_001088883_Xenopus_CRALTRIO ----KTRKRIFLHGN--------------NLNSLHQLIHPDCLPSEFGG

NP_001010852_Homo_CRALTRIO ----KTRKRIFLHGN--------------NLNSLHQLIHPEILPSEFGG

NP_001020716_Danio_CRALTRIO ----KTRKRIFMHGN--------------NLNSLHQLILPEILPSELGG

NP_001034288_Homo_CRALTRIO ----KIANRFFLHGS--------------DLNSLHTNLPRSILPKEYGG

NP_997755_Danio_CRALTRIO ----KMAERYVLHGS--------------DLASLHRVIPQSVLPQEYGG

NP_000361_Homo_CRALTRIO ----KIKERIHMHGN--------------NYKQSLLQHFPDILPLEYGG

NP_724195_Drosophila_CRALTRIO ----AMREKLYIHGS--------------DMTSLHKHINPEHLPKRYGG

NP_609120_Drosophila_CRALTRIO ----KLRKRIFFHGK--------------DYKSLISHIEAKALPPKYGG

NP_001079459_Xenopus_CRALTRIO ----KLRERVRTVK----------------AHELVNHIPKESLPEHLGG

NP_001081203_Xenopus_CRALTRIO ----KLRERVRTVK----------------AHELVNHIPKESLPEHLGG

NP_572576_Drosophila_CRALTRIO ----KLRERVFTVS----------------VPQLALHVPRKALPIHLGG

NP_002824_Homo_CRALTRIO ----KVRERIQILK----------------TSEVTQHLPRECLPENLGG

NP_724813_Drosophila_CRALTRIO ----KLQQRFHVYKN---------------LEQLNEVIPREYLPEEYGG

NP_610507_Drosophila_CRALTRIO ----KQKELLHIHST---------------VASLRKAIGLEYLPVEMGG

NP_001021496_caenorhabditis_Rh -SLIKIIDSSHLPKTVGGSY---------PYDHDEWLELRLDLEKWIWN

XP_001891671_brugia_RhoGEF -GLTRYIDSSQLTRDLGGSL---------FYDHDEWLETRLELERLIWQ

NP_009049_Homo_RhoGEF -GLTKVVDPSQLTPEFDGCL---------EYNHEEWIEIRVAFEDYISN

NP_001097996_danio_RhoGEF -GLSKVVDPSQLTADFEGSL---------DYNHEEWIEIRVAFEDFTGN

NP_001019831_Homo_RhoGEF -GLTKLVDPSQLTEEFDGSL---------DYNHEEWIELRLSLEEFFNS

XP_002598613_branchiostoma_Rho -GLTKCIDQSQLTSEFDGTL---------VYDHEEWIELRLALEEFVYS

NP_651960_drosophila_RhoGEF -SLNKIVESHQLTGDFEGQQ---------LYDHQQWTDARLAIEDFFWQ

XP_002578747_schistosoma_RhoGE -SLFREVDPSQLTAEMEGSL---------PYHHEEWIKIRCCLEEFFLF

XP_001635815_nematostella_RhoG -GLAKFIEPSQLTASLGGTL---------KYDHTDWLNMRLALEKFLYE

NP_835224.3_Homo_RhoGEF -KLTRYIEPCQLTEDFGGSL---------TYDHMDWLNKRLVFEKFTKE

NP_001085886_xenopus_RhoGEF -KLTRYIEPNQLTEEFGGSL---------TYDHMDWLNKRLVFEKFTKE

NP_878301_danio_RhoGEF -KLTRYIEPCQLTDEFGGSL---------LYDHLDWVNKRLVFEKFTKE

NP_001106203_Homo_RhoGEF PDLHGYIDKSQLTEDLGGTL---------DYCHSRWLCQRTAIESFALM

NP_055893_Homo_RhoGEF SDLHGYIDKSQLTRELGGTL---------EYRHGQWVNHRTAIENFALT

NP_001093325_Homo_RhoGEF SDLLTYIDDKQLTPELGGTL---------QYCHSEWIIFRNAIENFALT

NP_001033984_Drosophila_CRALTR ----KKQQGRLYVHGSKW-------------EALYNQIPKQYLPVEYGG

NP_651174_Drosophila_CRALTRIO ----KLPFKFVVHKKSED------------LYQHLPRDVMTIEYGGTNG

NP_496771_Caenorhabditis_CRALT ---EDYKEKIVITSEPAIG-------------AIQKHADKCFLPSDLGG

XP_002110939_trichoplax_RhoGEF YCHEEWIELRLMLEEFSRKSLG----VIHQMDKISDSVGNLNLPKELEA

XP_640612_Dictyostelium_BCH ----KFWKKLTYIDDLGE------------LFKTFPRE-QLALPNAIMM

XP_001420825_Ostreococcus_luc_ ----RLCNKVEYVETVCDLR---------DYVRLEGINEKLEIPAHVEE

NP_491993_Caenorhabditis_CRALT ------ITRLVEMPSESD-----------WKWSLAQIVNLDAWPMYWGG

EFA82125_poly_NF1 ----RAAKKVVFISNPVR-------------LFEHVVEAEHGLPATTLA

XP_638573_Dictyostelium_BCH ----GVLSKIIYHEN---------------MNKISKLISKCNIPKSIFS

NP_871646_Caenorhabditis_CRALT ----AASQRHDLLTFVTK-------------LSVTHYVPKSHLLEHQGG

XP_645940_Dictyostelium_BCH ---EKFWSKVEYLDYIQEIP-----------GVLDRSNIISKLPQSIKD

NP_171669_Arabidopsis_CRALTRIO -----TKKKIVFVENKKLT------------PTLLEDIDESQLPDIYGG

XP_646383_dict_CRALTRIO ---EVTLSKVRFINSKKVDG----------KRTFAELLEYVDIENLEQN

XP_001347356_Plasmodium_fal_BC ----KFWKKVEYIEKLED----------LFLKLNIKASKYLKYFPYIVQ

XP_002140404_Cryptosporidium_B ----KFMKKLEYADTIKDLCR--------FLPNIPTEEVCSRLPYVVQH

NP_569990_Drosophila_CRALTRIO ---EEVRNMIRYHTEG--------------MDSLYKEVPRDMLPNEYGG

NP_199584_Arabidopsis_CRALTRIO -----TRKKVHVLSGCGR-------------DELLKIMDFTSLPHFCRS

NP_508618_Caenorhabditis_CRALT ----QTREKVRFLDKDWK-------------NHLIEEIGEENIFMHWGG

XP_001702344_Chlamydomonas_BCH ----KAAHKIKKIDNLAQ------------IAEATNNEVKLESLGARFI

XP_646478_dict_CRALTRIO -----TKSKFIICSGNYR-------------EKLLKYIEADSIAPKLSG

NP_012390_Saccharomyces_CRALTR -----TRKKFVVLTDGSK------------LGQYLKDCPYEGYGGKDKK

XP_642079_dict_CRALTRIO ----KLISRIEICKN-------------------DQIFTTIDQSKVLFE

XP_645456_Dictyostelium_BCH ----KLKRKIHDIYNWKDLS---------QYIDIENIALPETSKDFITK

XP_001548197_yeast_NF1 -----FRRILRLSAKSEN-------------SAFHPKNVDYHLIGSLQD

XP_571310_yeast_NF1 ---APVGKNVVAASSPSE------------LVDHIPFS-SLALPEHTMA

NP_593281_Schizosaccharomyces_ ----KFTRKFRYLNSISD------------LNKYIPLS-HLKLPPIVYE

NP_001024391_Caenorhabditis_CR -----FSEKFRLHDRHFI-------------ETLTEDINIDDIPVSLGG

NP_872173_Caenorhabditis_CRALT ----NTVEKLAFLSNVED---------------LKKYLEPEAIPVEYGG

NP_497717_Caenorhabditis_CRALT ----ENLKRIEIISDKSD--------------LAGKFLPPWLVPKEYGG
