# Supplementary material for: Cross-Species Analyses Identify the BNIP-2 and Cdc42GAP Homology (BCH) Domain as a Distinct Functional Subclass of the CRAL_TRIO/Sec14 Superfamily
Source: PLoS One. 2012 Mar 27;7(3):e33863. doi: 10.1371/journal.pone.0033863 (PMC3313917; doi:10.1371/journal.pone.0033863)
Supplement: Supplementary file 4 [file pone.0033863.s002.doc]

Group-I

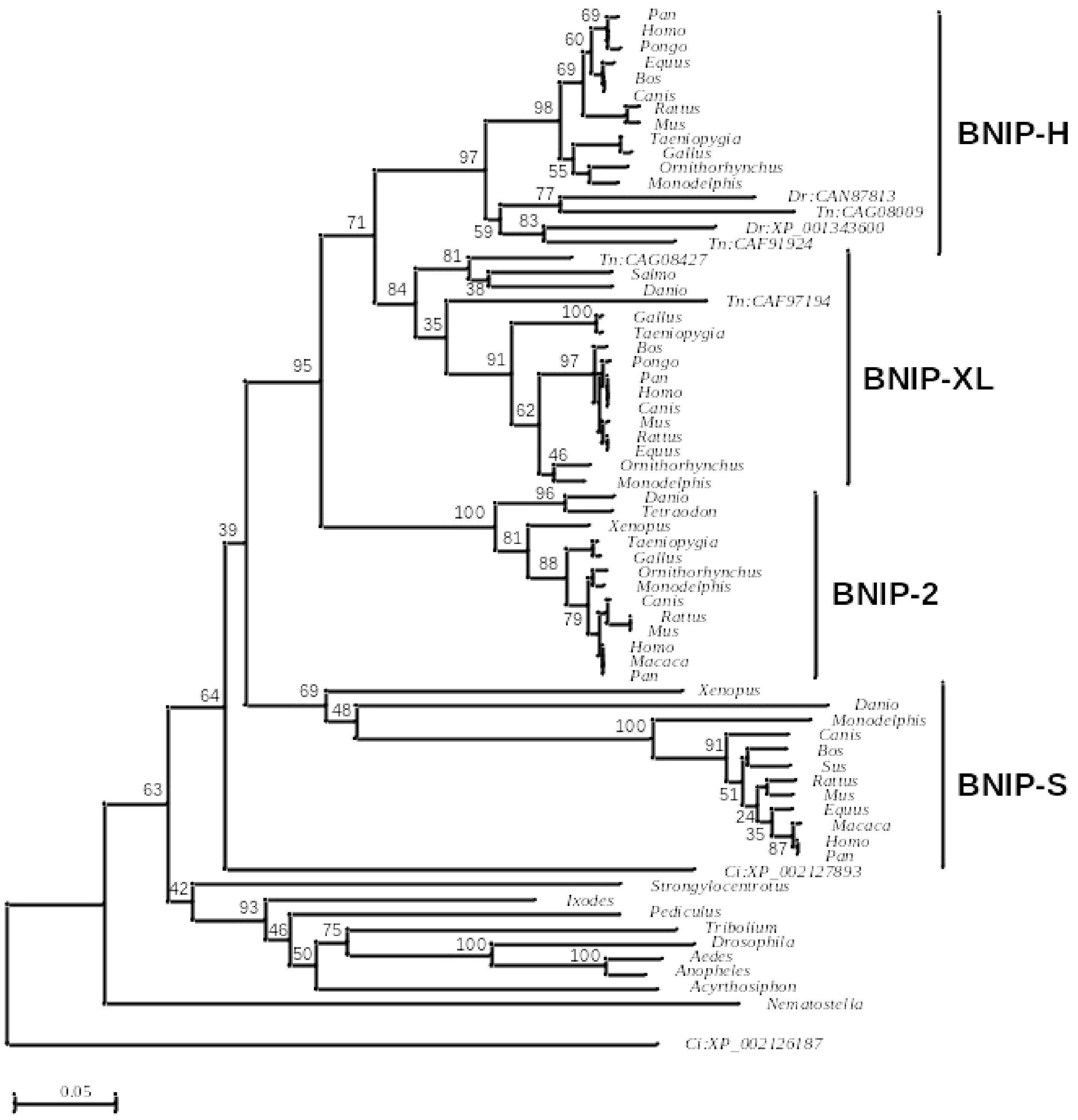

Pan
Homo
Pongo
Equus
Bos
Canis
Rattus
Mus
Taeniopygia
Gallus
Ornithorhynchus
Monodelphis
Dr:CAN87813
Tn:CAG08009
Dr:XP_001343600
Tn:CAF91924
BNIP-H
Tn:CAG08427
Salmo
Danio
Tn:CAF97194
Gallus
Taeniopygia
Bos
Pongo
Pan
Homo
Canis
Mus
Rattus
Equus
Ornithorhynchus
Monodelphis
BNIP-XL
Danio
Tetraodon
Xenopus
Taeniopygia
Gallus
Ornithorhynchus
Monodelphis
Canis
Rattus
Mus
Homo
Macaca
Pan
BNIP-2
Xenopus
Danio
Monodelphis
Canis
Bos
Sus
Rattus
Mus
Equus
Macaca
Homo
Pan
BNIP-S
Ci:XP_002127893
Strongylocentrotus
Ixodes
Pediculus
Tribolium
Drosophila
Aedes
Anopheles
Acyrthosiphon
Nematostella
Ci:XP_002126187
0.05

Group-IIA

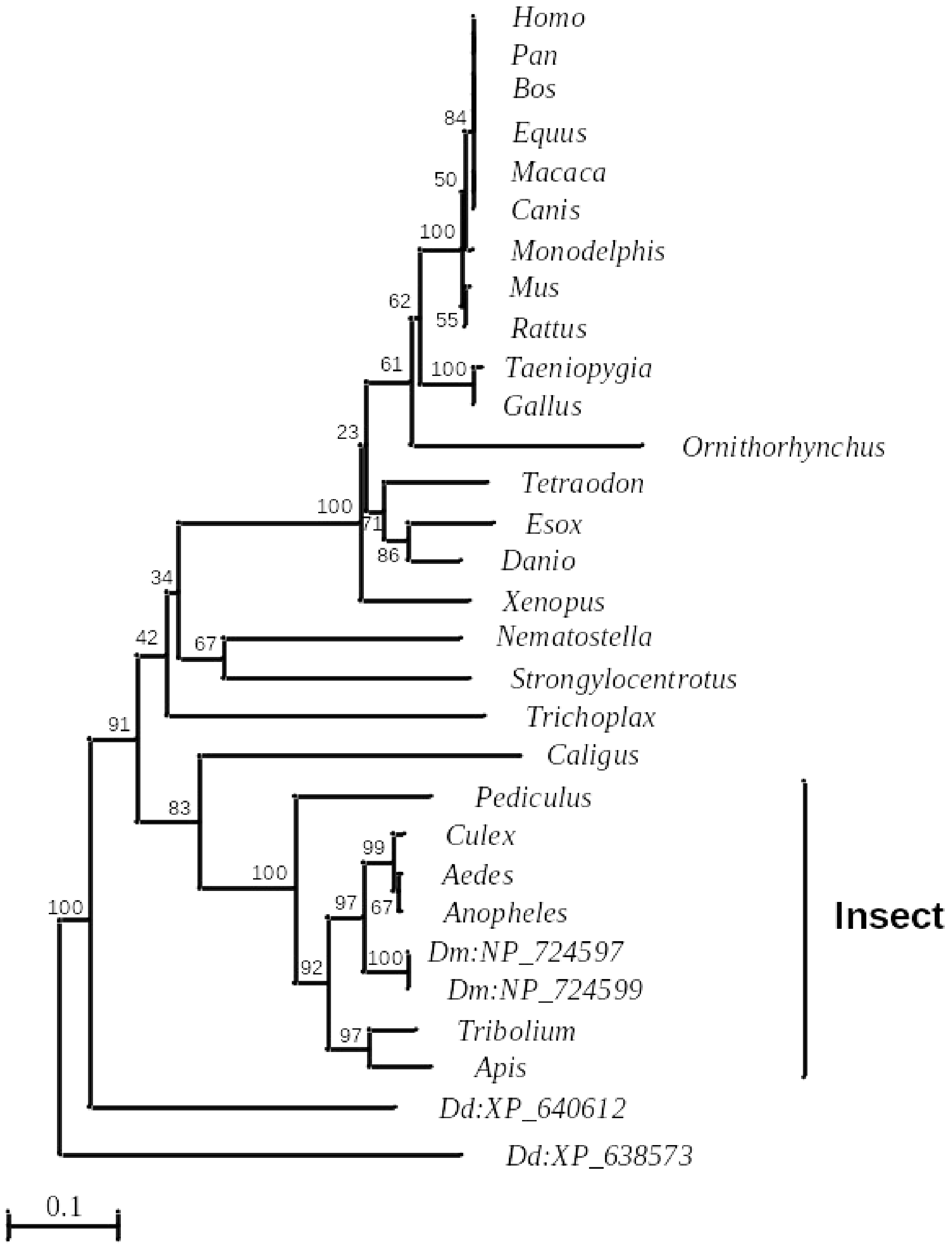
Homo
Pan
Bos
Equus
Macaca
Canis
Monodelphis
Mus
Rattus
Taeniopygia
Gallus
Ornithorhynchus
Tetraodon
Esox
Danio
Xenopus
Nematostella
Strongylocentrotus
Trichoplax
Caligus
Pediculus
Culex
Aedes
Anopheles
Dm:NP_724597
Dm:NP_724599
Tribolium
Apis
Dd:XP_640612
Dd:XP_638573
Insect
84
50
100
62
55
61
100
23
100
71
86
34
42
67
91
83
100
99
97
67
100
100
92
97
0.1

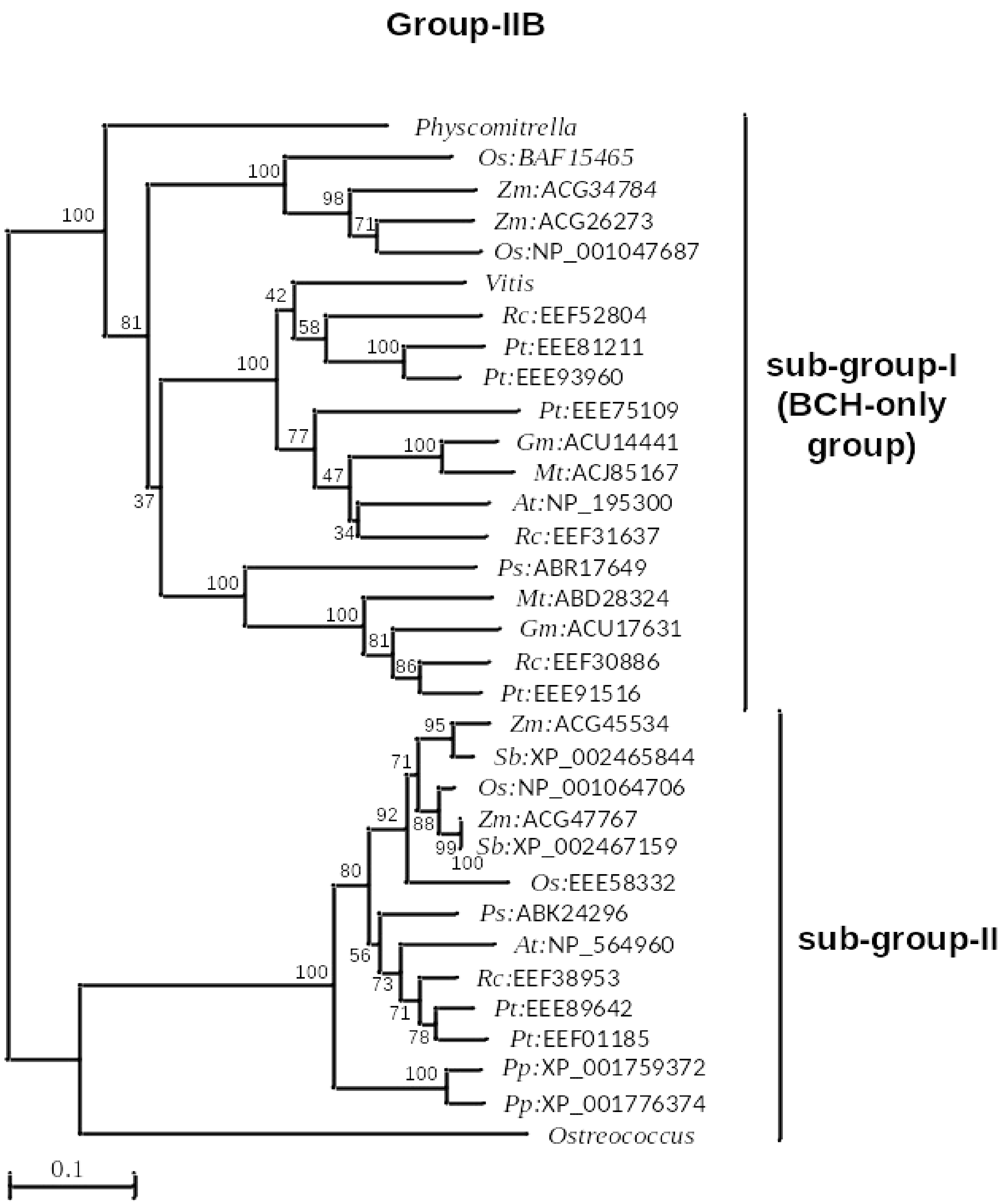
Group-IIB
Physcomitrella
Os:BAF15465
Zm:ACG34784
Zm:ACG26273
Os:NP_001047687
Vitis
Rc:EEF52804
Pt:EEE81211
Pt:EEE93960
Pt:EEE75109
Gm:ACU14441
Mt:ACJ85167
At:NP_195300
Rc:EEF31637
Ps:ABR17649
Mt:ABD28324
Gm:ACU17631
Rc:EEF30886
Pt:EEE91516
Zm:ACG45534
Sb:XP_002465844
Os:NP_001064706
Zm:ACG47767
Sb:XP_002467159
Os:EEE58332
Ps:ABK24296
At:NP_564960
Rc:EEF38953
Pt:EEE89642
Pt:EEF01185
Pp:XP_001759372
Pp:XP_001776374
Ostreococcus
sub-group-I (BCH-only group)
sub-group-II
0.1

Group-III

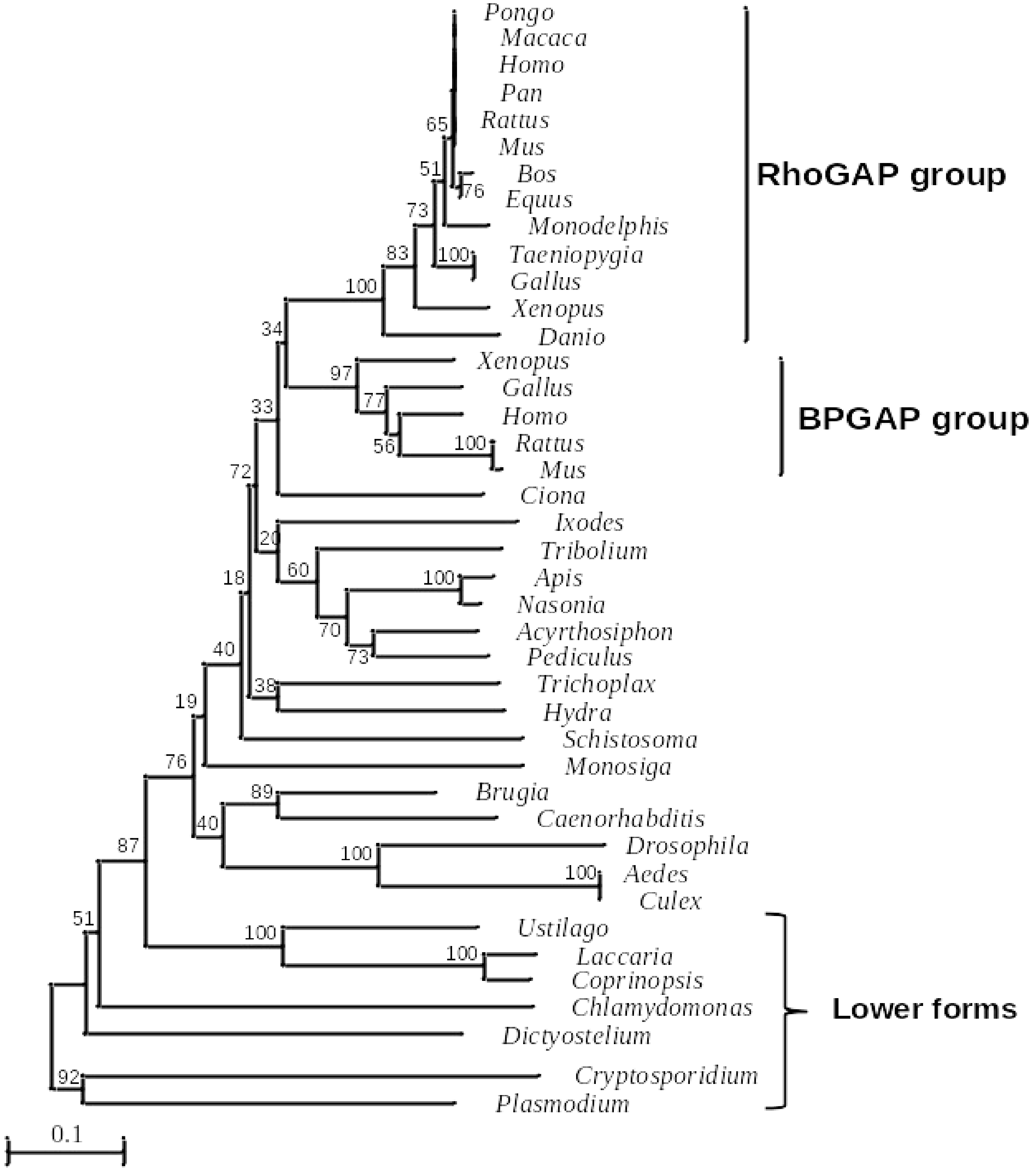

Pongo
Macaca
Homo
Pan
Rattus
Mus
Bos
Equus
Monodelphis
Taeniopygia
Gallus
Xenopus
Danio
Xenopus
Gallus
Homo
Rattus
Mus
Ciona
Ixodes
Tribolium
Apis
Nasonia
Acyrthosiphon
Pediculus
Trichoplax
Hydra
Schistosoma
Monosiga
Brugia
Caenorhabditis
Drosophila
Aedes
Culex
Ustilago
Laccaria
Coprinopsis
Chlamydomonas
Dictyostelium
Cryptosporidium
Plasmodium
RhoGAP group
BPGAP group
Lower forms
0.1
