# Supplementary material for: Cross-Species Analyses Identify the BNIP-2 and Cdc42GAP Homology (BCH) Domain as a Distinct Functional Subclass of the CRAL_TRIO/Sec14 Superfamily
Source: PLoS One. 2012 Mar 27;7(3):e33863. doi: 10.1371/journal.pone.0033863 (PMC3313917; doi:10.1371/journal.pone.0033863)

Alternative splicing in Group-I (BNIP2 clade) BCH domain

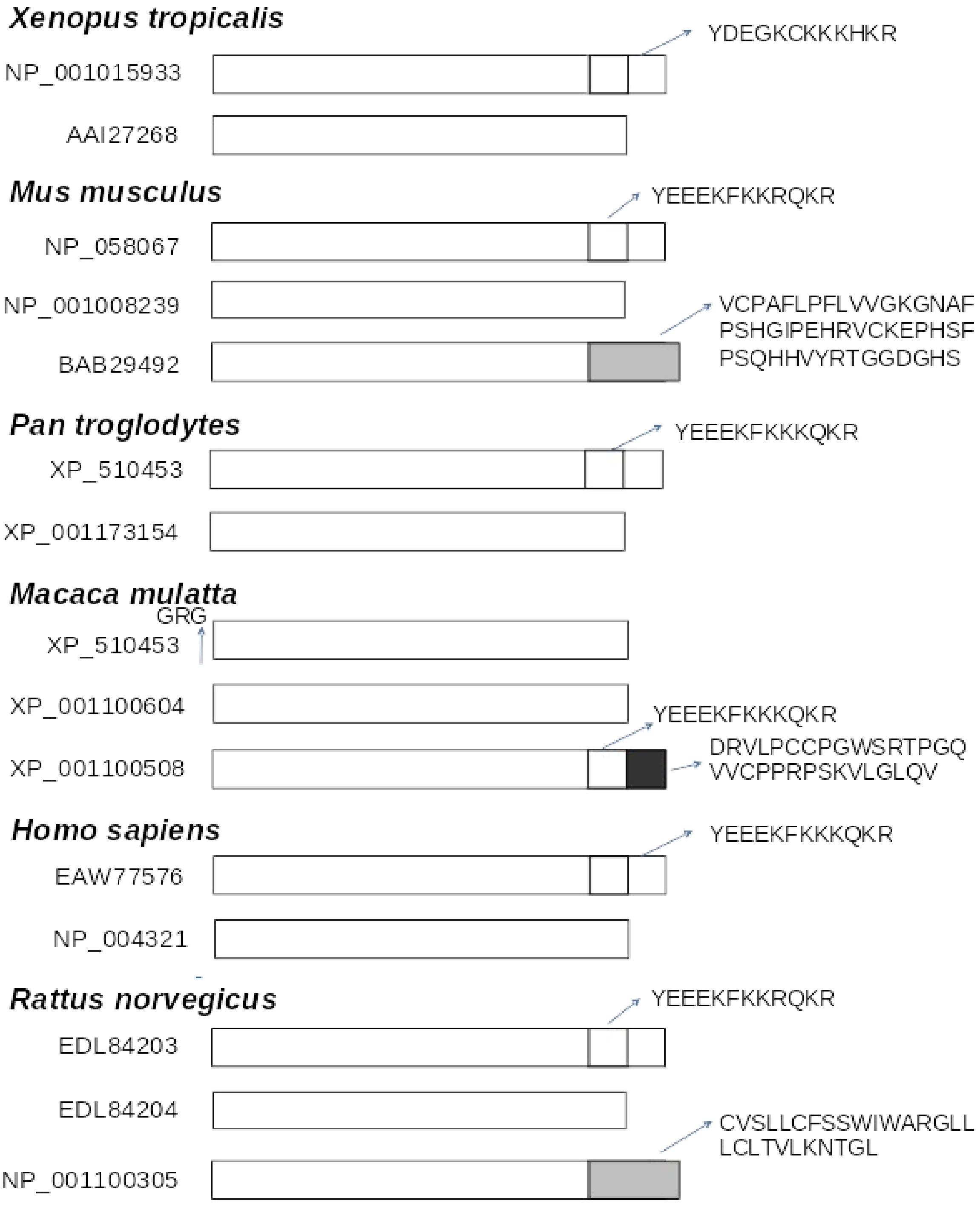

Alternative splicing in Group-I (BNIPS clade) BCH domain

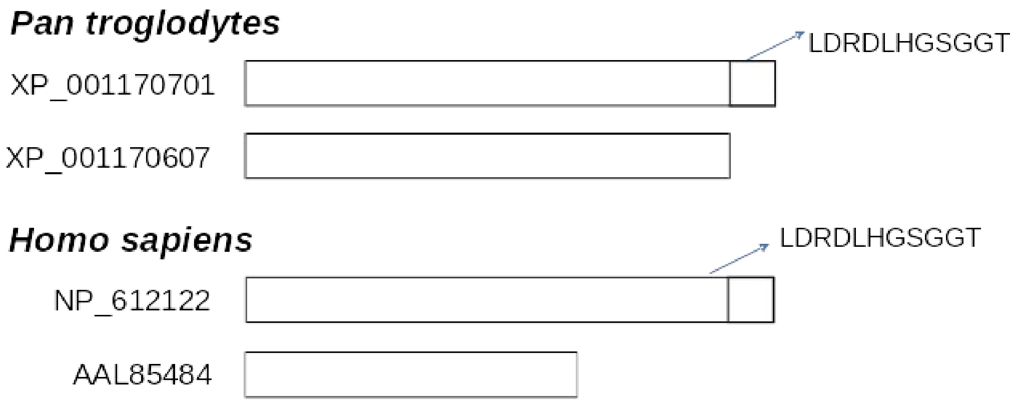

Alternative splicing in Group-I (BNIPXL clade) BCH domain

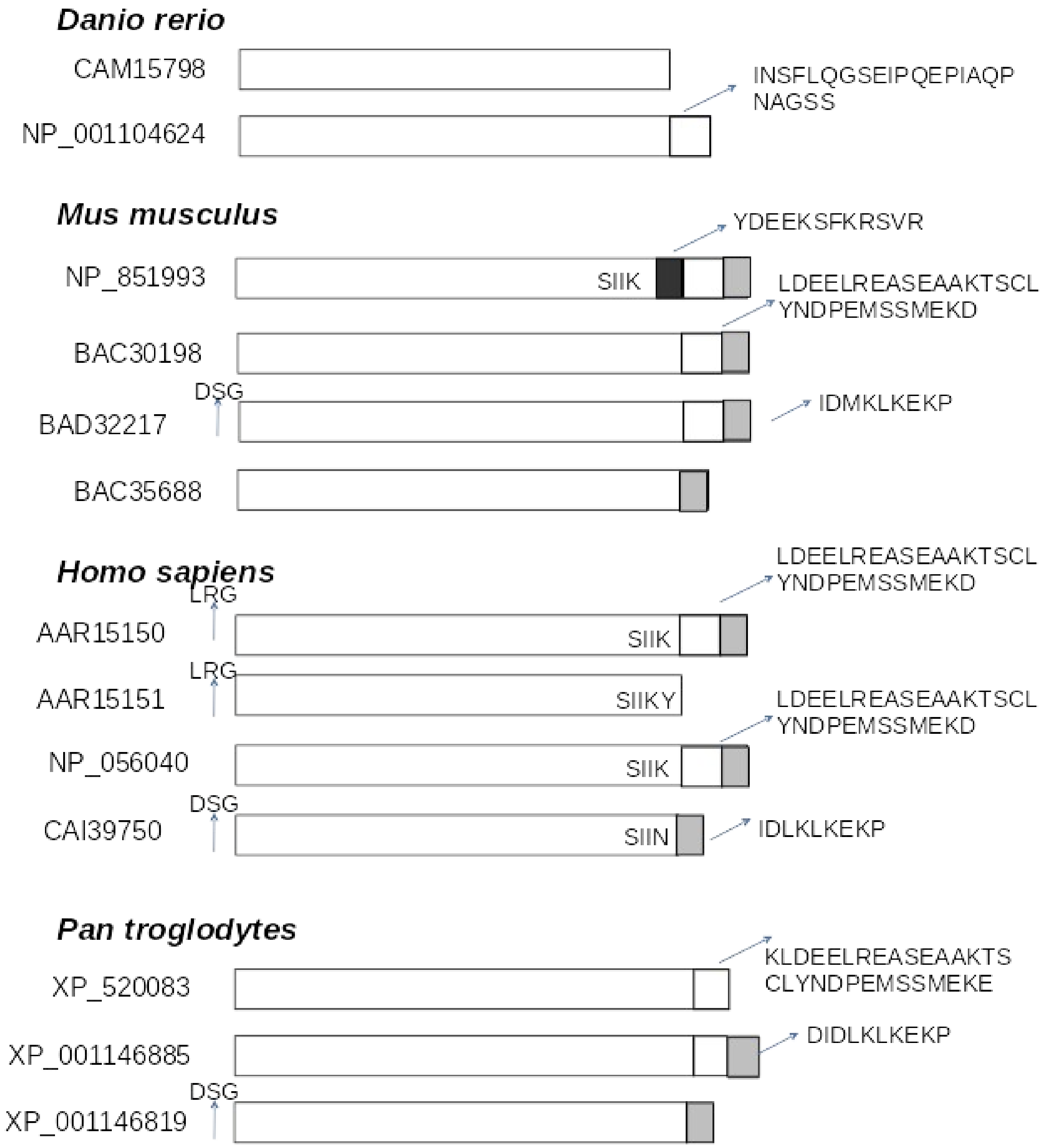

Alternative splicing in Group-II BCH domain

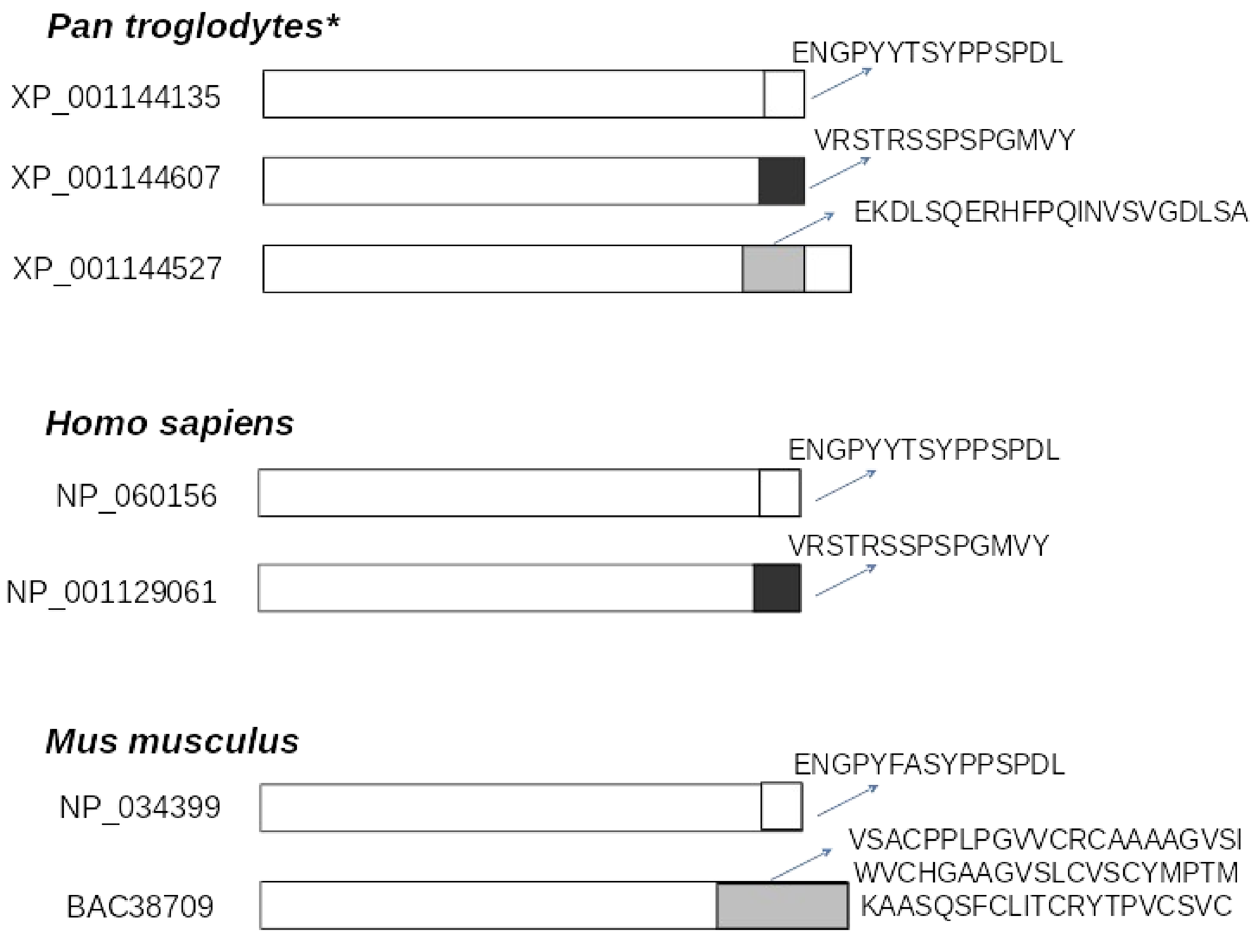

* More isoforms are defined in corresponding genomic entry NW_001229571.

Alternative splicing in Group-III BCH domain

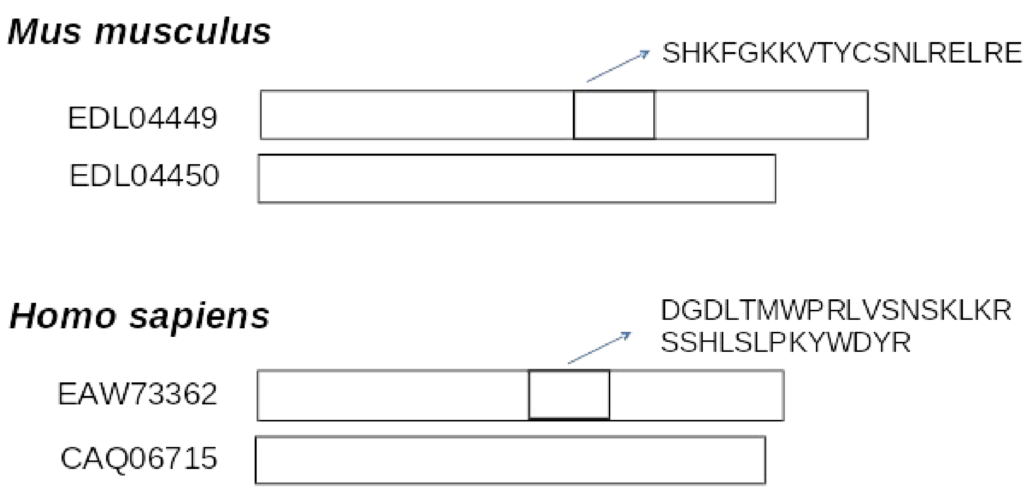

Supplement: Supplementary file 5 [file pone.0033863.s003.doc]
